# Physical exercise improves quality of life, depressive symptoms, and cognition across chronic brain disorders: a transdiagnostic systematic review and meta-analysis of randomized controlled trials

**DOI:** 10.1007/s00415-019-09493-9

**Published:** 2019-08-14

**Authors:** Meenakshi Dauwan, Marieke J. H. Begemann, Margot I. E. Slot, Edwin H. M. Lee, Philip Scheltens, Iris E. C. Sommer

**Affiliations:** 1 Department of Psychiatry, Brain Center Rudolf Magnus, University Medical Center Utrecht, Postbus 85500, 3508 GA Utrecht, The Netherlands; 2 Department of Clinical Neurophysiology and MEG Center, Amsterdam UMC, VU University Medical Center Amsterdam, Neuroscience Campus, Postbus 7057, 1007 MB Amsterdam, The Netherlands; 3 Department of Psychiatry, 2/F, New Clinical Building, University of Hong Kong, Queen Mary Hospital, Hong Kong, China; 4 Alzheimer Center and Department of Neurology, Neuroscience Campus Amsterdam, VU University Medical Center, Postbus 7057, 1007 MB Amsterdam, The Netherlands; 5 Department of Biomedical Sciences of Cells and Systems Section, University of Groningen, University Medical Center Groningen, Neuroimaging Center 3111, Deusinglaan 2, 9713 AW Groningen, The Netherlands; 6 Department of Biological and Medical Psychology, Faculty of Psychology, University of Bergen, Bergen, Norway

**Keywords:** Alzheimer’s disease, Multiple sclerosis, Parkinson’s disease, Depression, Schizophrenia, Physical exercise

## Abstract

We performed a meta-analysis to synthesize evidence on the efficacy and safety of physical exercise as an add-on therapeutic intervention for quality of life (QoL), depressive symptoms and cognition across six chronic brain disorders: Alzheimer’s disease, Huntington’s disease, multiple sclerosis, Parkinson’s disease, schizophrenia and unipolar depression. 122 studies ( = *k*) (*n* = 7231) were included. Exercise was superior to treatment as usual in improving QoL (*k* = 64, *n* = 4334, ES = 0.40, *p* < 0.0001), depressive symptoms (*k* = 60, *n* = 2909, ES = 0.78, *p* < 0.0001), the cognitive domains attention and working memory (*k* = 21, *n* = 1313, ES = 0.24, *p* < 0.009), executive functioning (*k* = 14, *n* = 977, ES = 0.15, *p* = 0.013), memory (*k* = 12, *n* = 994, ES = 0.12, *p* = 0.038) and psychomotor speed (*k* = 16, *n* = 896, ES = 0.23, *p* = 0.003). Meta-regression showed a dose–response effect for exercise time (min/week) on depressive symptoms (*β* = 0.007, *p* = 0.012). 69% of the studies that reported on safety, found no complications. Exercise is an efficacious and safe add-on therapeutic intervention showing a medium-sized effect on QoL and a large effect on mood in patients with chronic brain disorders, with a positive dose–response correlation. Exercise also improved several cognitive domains with small but significant effects.

**Electronic supplementary material:**

The online version of this article (10.1007/s00415-019-09493-9) contains supplementary material, which is available to authorized users.

## Introduction

Chronic brain disorders are associated with reduced quality of life (QoL) [1–4], high prevalence of low mood and depression, stress sensitivity and cognitive dysfunction [5, 6]. These sequelae are interdependent, as depressive mood and cognitive impairment are two main factors influencing QoL [1, 2, 4-8], while cognition is negatively influenced by depression [9]. Moreover, these general sequelae are associated with various adverse consequences such as poor treatment compliance, loss of independence and even mortality [10]. In treatment of brain disorders, current clinical practice tends to focus on improving disease-specific symptoms (e.g., tremor and rigidity in Parkinson’s disease, psychosis in schizophrenia). Notably, however, patients with brain disorders regard QoL and depressive mood as more important for their health status than disease-specific physical and mental symptoms [11]. Therefore, improvement of these common features should become an important target in treatment of chronic brain disorders.

Exercise therapy may positively affect QoL, depression and cognition across disorders. A leading example is stroke, in which physical exercise has shown favorable effects in improving a wide range of symptoms, such that it has now been incorporated and recommended in guidelines as part of the standard treatment [12–16]. In contrast, research on the efficacy of physical exercise in treatment of other brain disorders is still in its infancy and therefore not part of the standard care. Although several studies have investigated the effect of physical exercise in different chronic brain disorders such as Alzheimer’s disease (AD) [17, 18], multiple sclerosis (MS) [19–21], Parkinson’s disease (PD) [22, 23], Schizophrenia (Sz) [24, 25] and unipolar depression (UD) [26–28], results and mainly recommendations for clinical practice have been highly diverse [29]. As a consequence, current evidence for efficacy of exercise therapy is still disputed and exercise is not part of the regular care offer for patients with aforementioned disorders in most countries.

Of note, the above-mentioned chronic brain disorders share underlying pathophysiological mechanisms. As such, neuroinflammation [30–33], imbalance in same neurotransmitter (e.g., dopamine in Sz and PD [34, 35], serotonin in Sz and UD [36]) and growth factors (e.g., brain-derived neurotrophic factor; BDNF) [37, 38], and disturbed connectivity (e.g., in default-mode network) [39–42] have been implicated in the pathophysiology of many of these brain disorders. Furthermore, a recent genome-wide association study (GWAS) showed high degree of genetic overlap among many psychiatric disorders stating that the different psychiatric disorders do not reflect independent diseases but rather represent different overlapping phenotypes of the same clinical spectra [43].

The aforementioned shows how disease-specific research has de-emphasized and limited our understanding of substantial commonalities that exist across disorders. Considering the overlap in pathophysiology and clinical picture across chronic brain disorders, commonalities across disorders outweigh the differences indicating that transdiagnostic and disease-specific treatments might be at least equally effective. Therefore, by targeting the common functional relationships across disorders with transdiagnostic treatments, both disease-specific and common shared factors can be targeted during treatment. Physical exercise can be such a transdiagnostic treatment for chronic brain disorders.

The objective of this study is to quantitatively review the effect of additional physical exercise on QoL, depressive symptoms and cognition across the above-mentioned disorders. In addition, we aim to estimate the safety of exercise in aforementioned groups. There are of course more chronic brain disorders in which exercise therapy may be effective, but for reasons of feasibility we restricted this review to six different brain disorders of various origins.

## Method

### Literature search

This meta-analysis was performed according to the Preferred Reporting for Systematic Reviews and Meta-analysis (PRISMA) Statement [44]. A systematic search was performed in Pubmed (Medline), Embase, PsychInfo and Cochrane Database of Systematic Reviews (independently by MD, MS, and EL), using combinations of the following search terms: ‘Alzheimer’, ‘AD’, ‘Huntington’, ‘HD’, ‘multiple sclerosis’, ‘MS’, ‘Parkinson’, ‘PD’, ‘PDD’, ‘schizophrenia’, ‘psychosis’, ‘psychotic’, ‘depression’, ‘depressive’, ‘mood’, ‘affective’, ‘exercise’, ‘physical’, ‘training’, ‘endurance’, ‘aerobic’, ‘anaerobic’, ‘resistance’, ‘sport’ and ‘yoga’ (Online Resource 1), with no year or language limits. Additionally, the Web of Sciences databases and review articles were examined for cross-references. The search cutoff date was 15th of September 2018. When necessary, corresponding authors were contacted to provide full text details of the study outcome measures.

### Inclusion criteria

By consensus (between MD, MS, EL, and IS), the following studies were included:

1. Randomized controlled trials (RCTs) investigating the effect of any type of physical exercise as an add-on intervention on QoL, depressive symptoms and/or cognition
2. Studies investigating whole-body, or upper- or lower-body exercise (i.e., organ-specific exercise such as respiration muscle or pelvic muscle training were excluded)
3. Studies including patients with a diagnosis of AD, HD, MS (idiopathic) PD, Sz [24] and UD (according to a diagnostic interview) in both the intervention and control group (i.e., mixed study populations were excluded)
4. RCTs with a cross-over design providing data for the first study period
5. Studies investigating combined interventions when the control group received the same non-exercise component of the intervention (e.g., exercise + medication versus medication only)
6. Studies investigating rehabilitation programs, provided that physical exercise constituted a main part of the program
7. Studies reported sufficient information to compute common effect size (ES) statistics [i.e., mean and standard deviations (SDs), exact *F*, *p*, *t*, or *z* values] or corresponding authors could provide these data upon request
8. If multiple publications were retrieved that described the same cohort, only the sample with largest overall sample size and/or original data was included

### Exclusion criteria

1. Studies investigating same type of physical exercise in both the intervention and control group
2. Abstracts of studies (without full-text available) with insufficient information about the physical exercise intervention and/or outcome measures to calculate ES and untraceable corresponding information of the authors

### Outcome measures

The outcome measures included pre- and post-intervention assessments (i.e., measured directly after finishing the intervention and thus does not include follow-up measurements) of QoL, depressive symptom severity and/or cognition. For measurements of depressive symptoms, observer-rated scales were preferred over self-rated questionnaires because of its higher validity [45]. The scales used to measure depression comprised Hamilton Depression Rating Scale (HDRS) [46], Beck Depression Inventory (BDI) [47], Montgomery Asberg Depression Rating Scale (MADRS) [48], Geriatric Depression Scale (GDS) [49], Patient Health Questionnaire-9 (PHQ-9) [50], and Profile of Mood States (POMS) [51].

Based on the cognitive domains and/or cognitive tests investigated across studies and disorders, the following six cognitive domains were classified: attention and working memory (A&WM), executive functioning (EF), memory (M), psychomotor speed (PS), verbal fluency (VF) and global cognition (GC) (Online Resource 2). To combine studies across disorders, the most stringent control group per disorder [i.e., treatment as usual (TAU) allowing treatments such as disease-specific medication, reading newspapers, educational sessions but no active treatments such as occupational therapy] was used as a reference group.

### Assessment of risk of bias

According to the *Cochrane Handbook of Systematic Reviews of Interventions* [52], risk of bias was assessed for all eligible studies regarding selection bias, detection bias, attrition bias and reporting bias. Attrition bias was divided into assessment of incomplete outcome data (i.e., drop-out and exclusions) and intention-to-treat (ITT) analysis as ITT is considered the least biased method to measure intervention effects in RCTs [52]. Performance bias was not assessed, as it is usually not possible to blind study participants to whether or not exercise intervention is performed.

### Data analysis

All analyses were performed using Comprehensive Meta-Analysis Version 2.0. Per outcome measure, the effect of additional exercise (versus control group) was quantified for each study using Hedges’ *g* based on change scores (end of treatment minus baseline). When these were not reported, pre- and post-treatment mean values and SDs, or exact *F*, *p*, *t*, or *z* values were used. For studies that did not report exact SDs, these were calculated using the 95% confidence intervals (SD = sqrt(*N*) × [upper limit-lower limit]/[2 × 1.96]) or standard error (SE) (SD = SE × sqrt(*N*)).

To achieve a single pair-wise comparison between exercise and TAU, if a study investigated two or more types of exercise intervention, groups were combined for the main analysis [53] but studied separately in the moderator analysis (see further). The ES of the individual intervention groups were combined to calculate a composite ES by incorporating the ES and variance of each individual intervention while taking into account the correlation among the different interventions [54]. Likewise, when a study used more than one questionnaire to measure QoL or depressive symptoms, or multiple neuropsychological tests to measure a cognitive domain, a composite ES was calculated. As the correlation among interventions or test measures was mostly not reported, a correlation of 0.5 was taken for all the computations to avoid under- and overestimation of the overall ES [54].

Studies were combined in meta-analysis to calculate a mean weighted ES for each outcome measure (see Online Resource 3 for formulas). A random-effects model was considered appropriate given the heterogeneity across studies and diagnoses. Moreover, a random-effects model allows generalization of the results on population level [55]. ES were interpreted according to Cohen [56], with an ES of 0.2 indicating a small effect, 0.5 a medium and ≥ 0.8 a large effect. First, analyses were performed including all suitable studies per outcome measure. Subsequently, analyses were repeated by excluding outlier studies, defined as studies with standardized residual *z* scores of ES exceeding ± 1.96 (*p* < 0.05, two-tailed; shown in Figs. 2, 3, 4), studies with small total sample sizes (*n* < 20) because of high risk of sampling error in effect estimates [57] and studies with high risk of bias (i.e., considering the aim of the meta-analysis to study RCTs, studies classified as having high risk of bias on randomization and allocation concealment were excluded). ES with *p* < 0.05 were considered significant. Heterogeneity of results across studies was assessed by calculating the *Q*-statistic and *I*^2^-statistic. *Q*-Statistic tests the existence of heterogeneity and displays a Chi-square distribution with *k*−1 degrees of freedom (*k* = number of studies). *Q* values higher than the degrees of freedom indicate significant between-studies variability. *I*^2^ describes the percentage of total variation across studies due to heterogeneity rather than chance. *I*^2^ values of 25%, 50%, and 75% are considered as low, moderate, and high heterogeneity, respectively [58].

Potential publication bias was investigated by visual inspection of the funnel plots, with asymmetrical funnel plots indicating publication bias. When appropriate, the funnel plot asymmetry was tested with Egger’s test (*p* < 0.05, two-tailed) [59]. Additionally, Rosenthal’s fail-safe number (*N*_R_) was calculated for significant ES, estimating the number of unpublished studies with non-significant results needed to bring the observed result to non-significance [60].

#### Moderator analyses

Subgroup analyses were performed for ‘type of exercise’ classified as aerobic, resistance, or neuromotor exercise (e.g., yoga) according to the American College of Sports Medicine (ACSM) Guideline [61].

Since an insufficient number of studies examined the effect of flexibility exercise only, analysis was not feasible for this type of exercise.

Random effects meta-regression analyses were conducted to evaluate the effect of the following continuous moderator variables using the unrestricted maximum likelihood model:

- Exercise time (min/week)
- Total length of the intervention period (weeks)
- Age (overall mean age across study groups per study)

If a study reported a range for any of these variables, the mean value of the variable was calculated from the upper and lower bounds. To include each pair-wise comparison separately in these sensitivity analyses, for studies with multiple intervention groups but one shared control group, the total number of participants in the control group were evenly divided up among the comparisons [53].

Since a large number of the included studies did not provide sufficient information about the intensity and safety of the exercise intervention and most of the included studies (80%) investigated supervised exercise intervention, a sub- or meta-regression analysis was not possible to investigate the effect of these parameters. The intensity and safety of the exercise interventions were assessed qualitatively.

## Results

**Fig. 1:**
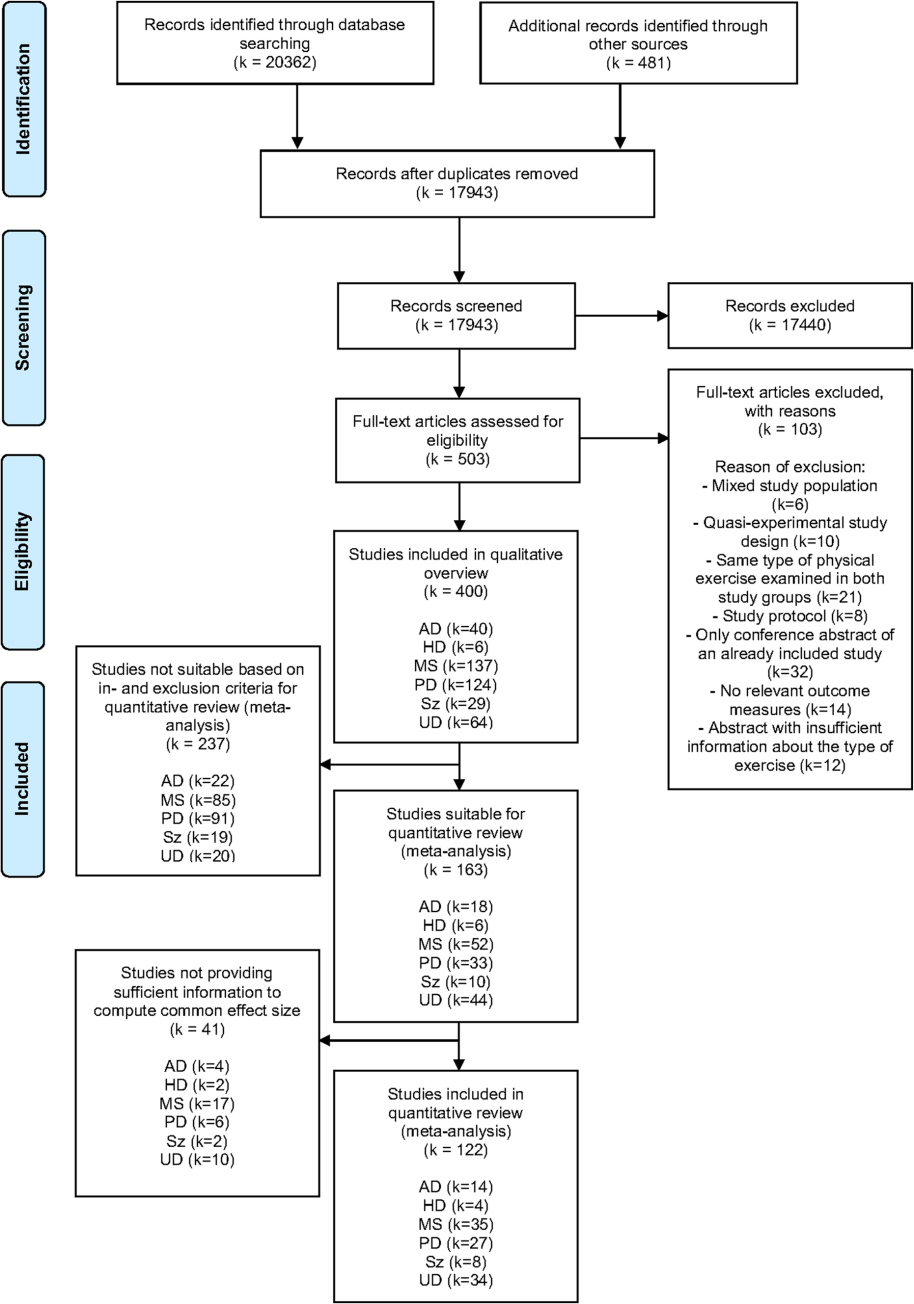
PRISMA flow chart of the literature search. *AD* Alzheimer’s disease, *HD* Huntington’s disease, *MS* multiple sclerosis, *PD* Parkinson’s disease, *Sz* schizophrenia, *UD* unipolar depression

A total of 400 articles investigating the effect of any type of exercise intervention for patients with chronic brain disorders were retrieved from the literature search (AD: *k* = 40, HD: *k* = 6, MS: *k* = 137, PD: *k* = 124, Sz: *k* = 29, UD: *k* = 64), see Fig. 1.

A descriptive overview of these studies is provided in Online Resource 4. Of these, 163 studies fulfilled the inclusion criteria and were eligible for meta-analysis [62–224]. Forty-one studies provided insufficient information to compute common effect size. Therefore, a final total of 122 studies could be combined in meta-analysis. Risk of bias of all the eligible studies is shown in Online Resource 5 with a corresponding elaborative assessment of the studies included in the meta-analysis.

### Quality of life

**Fig. 2:**
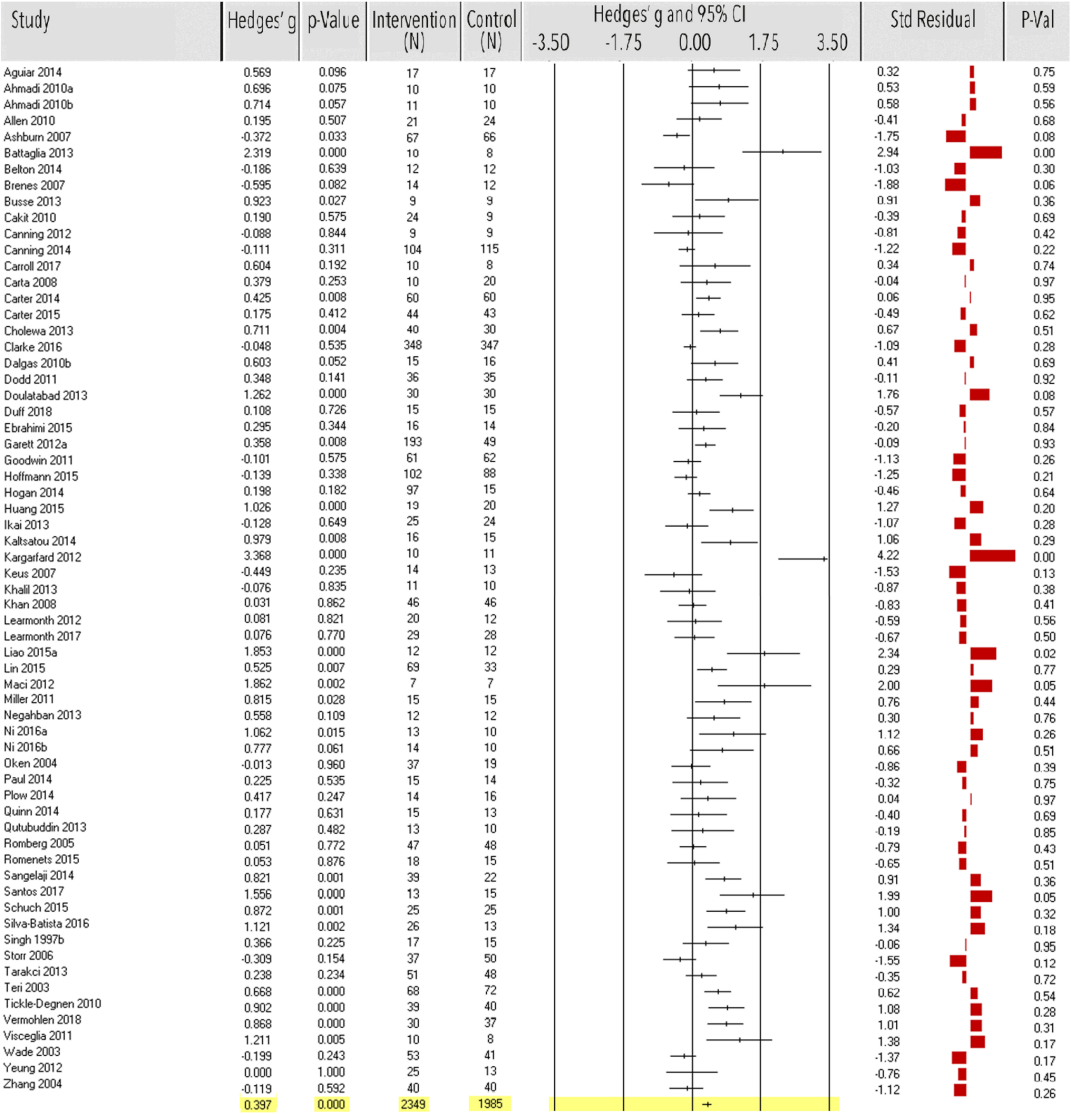
Meta-analysis of the effect of physical exercise on quality of life. Effect sizes (ES) per study and the overall ES are in Hedges’ *g* with corresponding *p* values and sample size of the intervention and control group. Standardized residual *z* scores of ES were used to detect outlier studies

Sixty-four studies (*n* = 4334) examined the effect of exercise on QoL. Exercise showed a significant medium-size effect (ES = 0.40, 95% CI 0.27–0.52, *p* < 0.0001; Fig. 2, Table 1). Heterogeneity was high [*Q*(63) = 250.18, *p* < 0.0001; *I*^2^ = 75%], indicating that 75% of the dispersion seen in Fig. 2 reflects difference in the true effect sizes while the remaining 25% can be attributed to random sampling error. Five studies [68, 142, 186, 200, 217] were identified as outliers, six studies [68, 119, 173, 200, 208, 216] had small sample sizes (*n* < 20) and another four studies [135, 140, 165, 193] were classified as having high risk of bias. After exclusion, ES decreased, but remained significant (*k* = 51, *n* = 3895, ES = 0.31, 95% CI 0.19–0.43, *p* < 0.0001). Heterogeneity decreased, but remained moderate to high [*Q*(50) = 159.13, *p* < 0.0001; *I*^2^ = 69%]. Funnel plot and Egger’s test indicated potential publication bias before [*t*(62) = 5.00, *p* < 0.0001, *N*_R_ = 1898], and after exclusion of the studies [*t*(49) = 3.39, *p* < 0.010, *N*_R_ = 847] but with very high fail-safe numbers (Table 1).

**Table 1:** Results of main and subgroup analyses across disorders

	Studies (*N*)	Patients (IG/CG)	Mean age (years) (range)	Exercise time (min/week) (range)	Intervention duration (weeks) (range)	Hedges’ *g*	95% CI	*P* value	*Q* statistic (*df*)	*I*^2^ (%)	Egger’s test	*N*_R_
*QoL*	64	2349/1985	53.3 (15.4–78.0)	116.50 (40.0–412.5)	12.20 (4.0–52.0)	**0.40**	0.27 to 0.52	**< 0.0001**	***Q*(63) = 250.18, *p* < 0.0001**	**75**	***t*(62) = 5.00, *p* < 0.0001**	**1898**
Without outliers	51	2091/1804	54.6 (15.4–78.0)	112.49 (40.0–360.0)	13.43 (4.0–52.0)	**0.31**	0.19 to 0.43	**< 0.0001**	***Q*(50) = 159.13, *p* < 0.0001**	**69**	***t*(49) = 3.39, *p* < 0.010**	**847**
Subgroup analysis												
Aerobic exercise	9	257/250				**0.45**	0.16 to 0.75	**0.003**	***Q*(8) = 27.36, *p* = 0.001**	**71**		
Neuromotor exercise	10	254/215				**0.35**	0.07 to 0.64	**0.013**	***Q*(9) = 22.63, *p* = 0.007**	**60**		
Resistance exercise	6	118/109				**0.57**	0.20 to 0.94	**0.003**	*Q*(5) = 4.19, *p* = 0.523	0		
All types of exercise	8	288/275				**0.37**	0.08 to 0.67	**0.014**	***Q*(7) = 26.93, *p* < 0.0001**	**74**		
*Depressive symptoms*	60	1635/1274	54.7 (15.4–83.0)	128.75 (40.0–300.00)	13.31 (1.4–52.0)	**0.78**	0.58 to 0.98	**< 0.0001**	***Q*(59) = 367.90, *p* < 0.0001**	**84**	***t*(58) = 6.10, *p* < 0.0001**	**3937**
Without outliers	43	1364/1066	54.3 (15.4–83.0)	118.14 (40.0–210.0)	14.61 (1.4–52.0)	**0.47**	0.32 to 0.62	**< 0.0001**	***Q*(42) = 130.55, *p* < 0.0001**	**68**	***t*(41) = 3.97, *p* < 0.001**	**1088**
Subgroup analysis												
Aerobic exercise	17	493/415				**0.40**	0.16 to 0.65	**0.001**	***Q*(16) = 49.41, *p* < 0.0001**	**68**		
Neuromotor exercise	8	176/143				**0.55**	0.18 to 0.91	**0.001**	*Q*(7) = 7.90, *p* = 0.342	11		
Resistance exercise	4	75/69				**0.96**	0.44 to 1.48	**< 0.001**	*Q*(3) = 6.22, *p* = 0.102	52		
All types of exercise	2	135/139				0.06	− 0.53 to 0.64	0.854	*Q*(1) = 2.35, *p* = 0.125	57		
**Cognition**												
*Attention and working memory*	21	794/519	55.8 (24.6–82.0)	118.57 (60.0–360.0)	15.36 (3.0–104.0)	**0.24**	0.06 to 0.41	**0.009**	***Q*(20) = 40.83, *p* = 0.004**	**51**	***t*(19) = 2.14, *p* = 0.046**	**55**
Without outlier	14	547/376	57.8 (24.6–82.0)	100.18 (60.0–180.0)	12.82 (6.0–24.0)	**0.25**	0.08 to 0.42	**0.004**	*Q*(13) = 20.83, *p* = 0.076	38	*t*(12) = 0.75, *p* = 0.466	
Subgroup analysis												
Aerobic exercise	8	287/184				0.06	− 0.16 to 0.29	0.575	*Q*(7) = 13.27, *p* = 0.066	47		
Neuromotor exercise	8	241/171				**0.39**	0.17 to 0.60	**0.001**	*Q*(7) = 6.84, *p* = 0.446	0		
*Executive functioning*	14	596/381	56.3 (24.6–78.8)	165.0 (60.0–480.0)	17.71 (3.0–52.0)	**0.15**	0.03 to 0.27	**0.013**	*Q*(13) = 12.30, *p* = 0.503	0	*t*(12) = 0.48, *p* = 0.641	
Without outlier	10	565/351	52.3 (24.6–78.0)	173.3 (60.0–480.0)	20.40 (3.0–52.0)	**0.17**	0.04 to 0.29	**0.009**	*Q*(9) = 4.58, *p* = 0.869	0	*t*(8) = 1.54, *p* = 0.163	
Subgroup analysis												
Aerobic exercise	7	316/241				**0.20**	0.06 to 0.35	**0.007**	*Q*(6) = 1.92, *p* = 0.927	0		
Neuromotor exercise	3	164/118				0.08	− 0.13 to 0.29	0.465	*Q*(2) = 5.41, *p* = 0.067	63		
*Memory*	12	609/385	51.9 (24.6–78.8)	139.38 (60.0–360.0)	13.50 (3.0–36.0)	**0.12**	0.07 to 0.24	**0.038**	*Q*(11) = 10.74, *p* = 0.465	0	*t*(10) = 0.59, *p* = 0.568	
Without outlier	9	582/357	54.7 (24.6–78.8)	143.33 (60.0–360.0)	16.11 (3.0–36.0)	0.09	− 0.03 to 0.21	0.127	*Q*(8) = 4.81, *p* = 0.777	0	*t*(7) = 0.90, *p* = 0.399	
Subgroup analysis												
Aerobic exercise	7	394/262				0.11	− 0.02 to 0.24	0.107	*Q*(6) = 2.94, *p* = 0.817	0		
Neuromotor exercise	4	179/84				0.14	− 0.10 to 0.38	0.254	*Q*(3) = 0.44, *p* = 0.933	0		
*Psychomotor speed*	16	509/387	53.1 (24.6–78.8)	115.0 (60.0–180.0)	13.88 (3.0–36.0)	**0.23**	0.08 to 0.38	**0.003**	*Q*(15) = 19.02, *p* = 0.213	21	***t*(14) = 2.36, *p* = 0.035**	**42**
Without outlier	10	454/332	53.0 (24.6–78.8)	112.5 (60.0–180.0)	28.86 (9.0–36.0)	**0.14**	0.005 to 0.27	**0.042**	*Q*(9) = 8.56, *p* = 0.479	0	*t*(8) = 1.02, *p* = 0.338	
Subgroup analysis												
Aerobic exercise	8	338/247				0.09	− 0.07 to 0.24	0.276	*Q*(7) = 7.04, *p* = 0.425	1		
Neuromotor exercise	2	60/26				0.32	− 0.08 to 0.71	0.116	*Q*(1) = 0.66, *p* = 0.416	0		
*Verbal fluency*	6	303/237	66.7 (49.6–78.8)	176.25 (60.0–480.0)	20.17 (9.0–52.0)	0.24	− 0.07 to 0.55	0.134	*Q*(5) = 14.36, *p* = 0.014	65	*t*(4) = 3.09, *p* = 0.037	3
Without outlier	5	288/222	65.7 (49.6–78.8)	193.50 (60.0–480.0)	21.80 (9.0–52.0)	0.06	− 0.15 to 0.27	0.569	*Q*(4) = 5.55, *p* = 0.236	28	*t*(3) = 2.48, *p* = 0.089	
*Global cognition*	15	376/349	71.1 (50.4–84.0)	157.86 (45.0–480.0)	19.13 (4.0–52.0)	0.30	− 0.03 to 0.63	0.076	*Q*(14) = 60.79, *p* < 0.0001	77	*t*(13) = 0.11, *p* = 0.917	
Without outliers	10	321/299	69.4 (50.4–82.0)	163.89 (45.0–480.0)	21.90 (8.0–52.0)	**0.39**	0.09 to 0.68	**0.010**	***Q*(9) = 26.15, *p* = 0.002**	**66**	*t*(8) = 1.14, *p* = 0.286	
Subgroup analysis												
Aerobic exercise	4	148/131				0.22	− 0.15 to 0.58	0.246	*Q*(3) = 7.23, *p* = 0.064	59		
Resistance exercise	1	26/13				**1.45**	0.56 to 2.34	**0.001**				

Results in bold indicate significant effect size

*CG* control group, *df* degrees of freedom, *IG* intervention group, *N*_*R*_ Rosenthal’s fail-safe number, *min/week* minutes per week

Within-disorder analysis showed a positive effect of exercise on QoL in patients with MS, PD and Sz (Table 2).

**Table 2:** Results per disorder for all outcome measures

Outcome measure	Studies (*N*)	Patients (IG/CG)	Hedges’ *g*	95% CI	*P *value	***Q*** statistic (*df*)	*I*^2^ (%)	Egger’s test^a^	*N*_R_
QoL									
Alzheimer’s disease	5	234/224	0.40	− 0.10 to 0.91	0.119	*Q*(4) = 23.51, *p* < 0.0001	83	*t*(3) = 1.30, *p* = 0.283	
Without outlier	4	227/217	0.22	− 0.24 to 0.68	0.345	*Q*(3) = 15.90, *p* = 0.001	81	*t*(2) = 0.47, *p* = 0.688	
Huntington’s disease	3	35/32	0.31	− 0.25 to 0.88	0.280	*Q*(2) = 3.39, *p* = 0.184	41	*t*(1) = 5.05, *p* = 0.124	
Without outlier	2	26/23	0.05	− 0.46 to 0.56	0.850	*Q*(1) = 0.24, *p* = 0.626	0		
Multiple sclerosis	25	909/641	**0.41**	**0.24 to 0.58**	**< 0.0001**	***Q*(24) = 72.61, *p* < 0.0001**	**67**	***t*(23) = 2.20, *p* = 0.038**	**380**
Without outlier	21	749/551	**0.39**	**0.25 to 0.54**	**< 0.0001**	***Q*(20) = 34.99, *p* = 0.020**	**43**	*t*(19) = 1.15, *p* = 0.263	
Parkinson’s disease	19	887/852	**0.31**	**0.08 to 0.54**	**0.009**	***Q*(18) = 81.45, *p* < 0.0001**	**78**	***t*(17) = 2.94, *p* = 0.009**	**59**
Without outlier	14	825/793	0.18	− 0.04 to 0.41	0.112	*Q*(13) = 52.43, *p* < 0.0001	75	*t*(12) = 2.05, *p* = 0.063	
Schizophrenia	5	130/88	**0.89**	**0.22 to 1.55**	**0.009**	***Q*(4) = 21.02, *p* < 0.0001**	**81**	*t*(3) = 1.67, *p* = 0.194	
Without outlier	3	110/72	0.43	− 0.13 to 0.99	0.130	*Q*(2) = 6.35, *p* = 0.042	68	*t*(1) = 0.11, *p* = 0.931	
Unipolar depression	7	154/148	0.34	− 0.04 to 0.72	0.082	*Q*(6) = 10.08, *p* = 0.004	69	*t*(5) = 0.64, *p* = 0.552	
Depressive symptoms									
Alzheimer’s disease	5	264/254	**0.80**	**0.12 to 1.49**	**0.022**	***Q*(4) = 48.15, *p* < 0.0001**	**92**	***t*(3) = 3.46, *p* = 0.041**	**24**
Without outlier	3	237/227	0.05	− 0.16 to 0.24	0.653	*Q*(2) = 2.38, *p* = 0.305	16	*t*(1) = 0.005, *p* = 0.997	
Huntington’s disease	2	24/24	0.40	− 0.76 to 1.56	0.496	*Q*(1) = 4.03, *p* = 0.045	75		
Multiple sclerosis	14	327/249	**0.45**	**0.12 to 0.79**	**0.007**	***Q*(13) = 47.78, *p* < 0.0001**	**73**	***t*(12) = 2.30, *p* = 0.040**	**69**
Without outlier	13	291/231	**0.23**	**0.06 to 0.40**	**0.010**	*Q*(12) = 9.61, *p* = 0.650	0	***t*(11) = 3.59, *p* = 0.004**	**18**
Parkinson’s disease	5	116/100	0.05	− 0.36 to 0.45	0.822	*Q*(4) = 7.91, *p* = 0.095	49	*t*(3) = 0.83, *p* = 0.469	
Without outlier	3	89/77	-0.04	− 0.63 to 0.55	0.895	*Q*(2) = 6.22, *p* = 0.045	68	*t*(1) = 0.20, *p* = 0.874	
Schizophrenia	2	46/21	**0.73**	**0.20 to 1.26**	**0.007**	*Q*(1) = 0.89, *p* = 0.347	0		
Without outlier	1	42/15	**0.62**	**0.04 to 1.19**	**0.037**	*Q*(0) = 0.00, *p* = 1.000	0		
Unipolar depression	32	858/626	**1.08**	**0.78 to 1.38**	**< 0.0001**	***Q*(31) = 210.96, *p* < 0.0001**	**85**	***t*(30) = 4.83, *p* < 0.0001**	**2024**
Without outliers	23	736/523	**0.88**	**0.62 to 1.14**	**< 0.0001**	***Q*(22) = 101.96, *p* < 0.0001**	**78**	***t*(21) = 4.18, *p* < 0.001**	**980**
**Cognition**									
Attention and working memory									
Alzheimer’s disease	3	44/43	0.28	− 0.13 to 0.69	0.185	*Q*(2) = 2.30, *p* = 0.317	13	*t*(1) = 5.29, *p* = 0.119	
Multiple sclerosis	5	117/95	0.23	− 0.04 to 0.49	0.089	*Q*(4) = 4.16, *p* = 0.384	4	*t*(3) = 0.67, *p* = 0.550	
Without outlier	4	112/90	0.24	− 0.07 to 0.56	0.134	*Q*(3) = 4.16, *p* = 0.245	28	*t*(2) = 1.16, *p* = 0.365	
Parkinson’s disease	5	89/82	**0.50**	**0.20 to 0.80**	**0.001**	*Q*(4) = 2.63, *p* = 0.622	0	*t*(3) = 0.05, *p* = 0.962	
Without outliers	2	57/54	0.41	− 0.12 to 0.94	0.129	*Q*(1) = 1.61, *p* = 0.205	38		
Schizophrenia	4	373/184	0.07	− 0.41 to 0.55	0.776	*Q*(3) = 14.57, *p* = 0.002	79	*t*(2) = 0.54, *p* = 0.642	
Without outlier	3	365/174	0.04	− 0.51 to 0.60	0.879	*Q*(2) = 14.32, *p* = 0.001	86	*t*(1) = 1.44, *p* = 0.386	
Unipolar depression	4	171/115	0.22	− 0.24 to 0.68	0.351	*Q*(3) = 8.72, *p* = 0.033	66	*t*(2) = 1.14, *p* = 0.373	
Without outlier	3	163/107	0.17	− 0.36 to 0.70	0.540	*Q*(2) = 7.79, *p* = 0.020	74	*t*(1) = 0.78, *p* = 0.578	
Executive functioning									
Alzheimer’s disease	3	78/82	0.03	− 0.58 to 0.64	0.921	*Q*(2) = 5.21, *p* = 0.074	62	*t*(1) = 0.0005, *p* = 1.000	
Without outlier	2	71/75	-0.17	− 0.86 to 0.52	0.628	*Q*(1) = 3.16, *p* = 0.076	68		
Multiple sclerosis	4	76/56	0.15	− 0.18 to 0.47	0.370	*Q*(3) = 2.00, *p* = 0.572	0	*t*(2) = 0.49, *p* = 0.673	
Without outlier	3	71/51	0.21	− 0.13 to 0.56	0.223	*Q*(2) = 0.74, *p* = 0.692	0	*t*(1) = 0.82, *p* = 0.564	
Parkinson’s disease	2	24/16	0.28	− 0.25 to 0.80	0.306	*Q*(1) = 0.70, *p* = 0.402	0		
Without outlier	1	15/8	0.08	− 0.62 to 0.78	0.827				
Schizophrenia	2	263/125	0.17	− 0.21 to 0.55	0.386	*Q*(1) = 2.88, *p* = 0.090	65		
Unipolar depression	2	146/91	0.20	− 0.01 to 0.42	0.065	*Q*(1) = 0.04, *p* = 0.835	0		
Memory									
Alzheimer’s disease	3	127/110	0.05	− 0.18 to 0.28	0.666	*Q*(2) = 0.75, *p* = 0.688	0	*t*(1) = 0.31, *p* = 0.811	
Multiple sclerosis	2	48/30	0.48	− 0.53 to 1.48	0.352	*Q*(1) = 4.64, *p* = 0.031	78		
Schizophrenia	3	271/135	0.13	− 0.07 to 0.33	0.201	*Q*(2) = 0.89, *p* = 0.641	0	*t*(1) = 1.01, *p* = 0.496	
Without outlier	2	263/125	0.12	− 0.09 to 0.33	0.250	*Q*(1) = 0.79, *p* = 0.376	0		
Unipolar depression	3	154/99	0.17	− 0.04 to 0.38	0.104	*Q*(2) = 0.77, *p* = 0.680	0	*t*(1) = 0.69, *p* = 0.615	
Without outlier	2	146/91	0.16	− 0.05 to 0.38	0.136	*Q*(1) = 0.67, *p* = 0.413	0		
Psychomotor speed									
Alzheimer’s disease	3	127/113	0.49	− 0.32 to 1.29	0.237	*Q*(2) = 10.38, *p* = 0.006	81	*t*(1) = 1.62, *p* = 0.352	
Multiple sclerosis	6	133/113	0.24	− 0.008 to 0.48	0.058	*Q*(5) = 3.22, *p* = 0.667	0	*t*(4) = 0.68, *p* = 0.533	
Without outliers	4	118/99	0.22	− 0.04 to 0.48	0.099	*Q*(3) = 2.63, *p* = 0.452	0	*t*(2) = 0.20, *p* = 0.858	
Schizophrenia	2	77/43	**0.45**	**0.07 to 0.83**	**0.020**	*Q*(1) = 0.02, *p* = 0.886	0		
Without outlier	1	69/33	**0.44**	**0.02 to 0.85**	**0.040**				
Unipolar depression	3	154/99	0.18	− 0.05 to 0.41	0.133	*Q*(2) = 1.84, *p* = 0.398	0	*t*(1) = 1.74, *p* = 0.332	
Without outlier	2	146/91	0.14	− 0.10 to 0.38	0.238	*Q*(1) = 0.48, *p* = 0.487	0		
Verbal fluency									
Alzheimer’s disease	4	188/178	0.27	− 0.20 to 0.74	0.264	*Q*(3) = 12.23, *p* = 0.007	75	*t*(2) = 2.92, *p* = 0.100	
Global cognition									
Alzheimer’s disease	10	299/287	0.21	− 0.21 to 0.63	0.332	*Q*(9) = 50.92, *p* < 0.0001	82	*t*(8) = 0.19, *p* = 0.853	
Without outliers	7	271/260	**0.32**	**0.02 to 0.63**	**0.039**	***Q*(6) = 16.63, *p* = 0.011**	**64**	*t*(5) = 0.81, *p* = 0.456	
Huntington’s disease	2	24/26	0.14	− 0.40 to 0.68	0.613	*Q*(1) = 0.15, *p* = 0.702	0		
Parkinson’s disease	3	53/36	0.71	− 0.03 to 1.45	0.060	*Q*(2) = 5.51, *p* = 0.064	64	*t*(1) = 0.07, *p* = 0.957	
Without outliers	1	26/13	**1.45**	**0.69 to 2.21**	**< 0.0001**				

Results in bold indicate significant effect size

*CG *control group, *df* degrees of freedom, *IG* intervention group, *N*_*R*_ Rosenthal’s fail-safe number

^a^Egger’s test cannot be performed for *k* ≤ 2

### Depressive symptoms

**Fig. 3:**
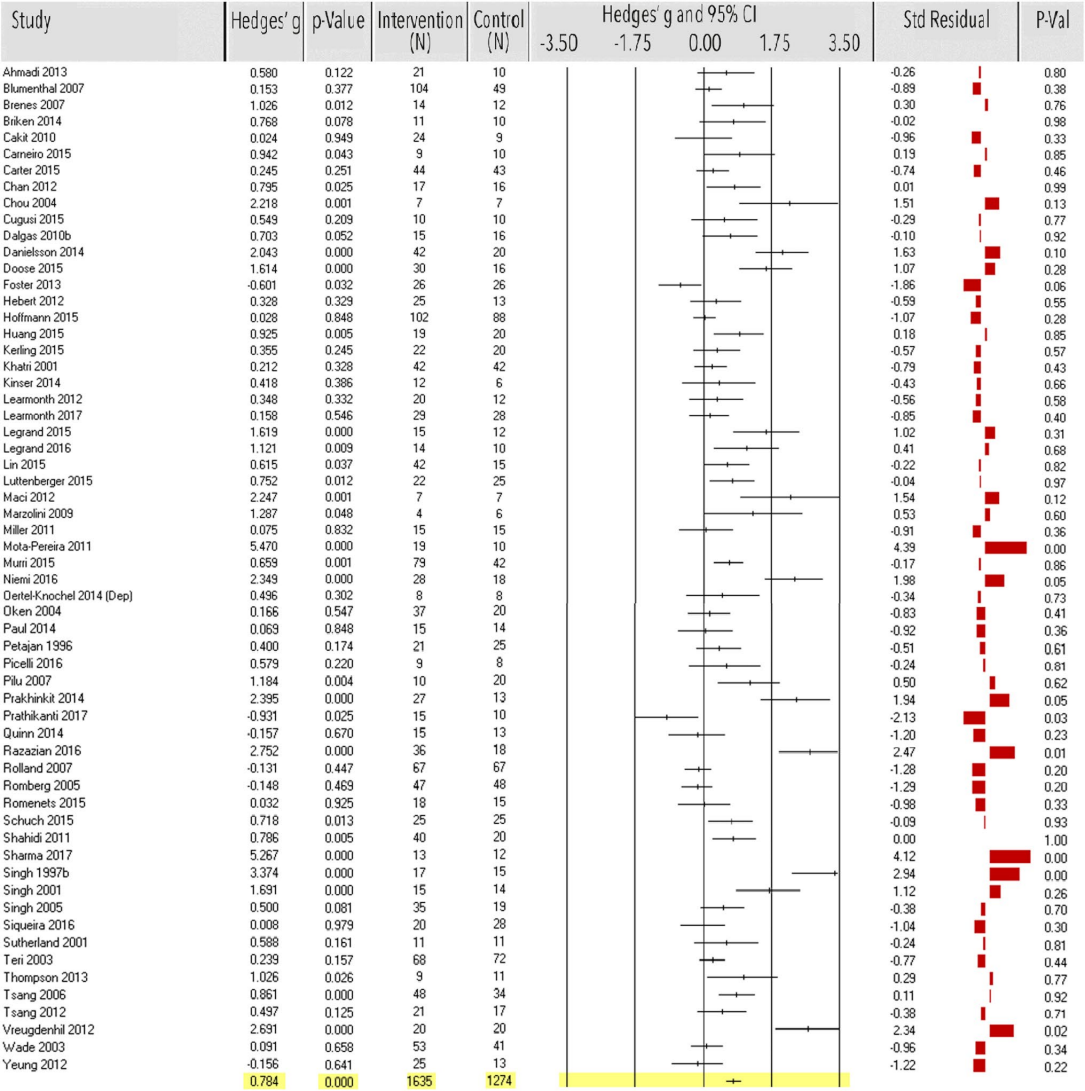
Meta-analysis of the effect of physical exercise on depressive symptoms. Effect sizes (ES) per study and the overall ES are in Hedges’ *g* with corresponding *p* values and sample size of the intervention and control group. Standardized residual *z* scores of ES were used to detect outlier studies

Sixty studies (*n* = 2909) showed a significant large-size effect of exercise on depressive symptoms (ES = 0.78, 95% CI 0.58–0.98, *p* < 0.0001; Fig. 3), with high heterogeneity [*Q*(59) = 367.90, *p* < 0.0001; *I*^2^ = 84%; Table 1]. Excluding eight outliers [75, 101, 104, 108, 112, 159, 220, 221], seven small studies (*n* < 20) [68, 82, 87, 95, 190, 207, 225] and two studies [99, 193] with high risk of bias decreased the overall ES to a medium effect (*k* = 43 *n* = 2430, ES = 0.47, 95% CI 0.32–0.62, *p* < 0.0001). Heterogeneity reduced to moderate to high [*Q*(42) = 130.55, *p* < 0.0001; *I*^2^ = 68%]. Funnel plot and Egger’s test indicated potential publication bias [*t*(58) = 6.10, *p* < 0.0001, *N*_R_ = 3937], which remained after exclusion of the outliers [*t*(41) = 3.97, *p* < 0.001, *N*_R_ = 1088; Table 1].

Within-disorder analysis showed a positive effect of exercise on depressive symptoms in AD, MS, Sz and UD (Table 2).

### Cognition

Of the 120 studies, 36 studies (AD: *k* = 12, HD: *k* = 3, MS: *k* = 7, PD: *k* = 7, Sz: *k* = 3, UD: *k* = 4), examining 2125 patients, evaluated cognitive functioning and were included.

#### Attention and working memory

**Fig. 4:**
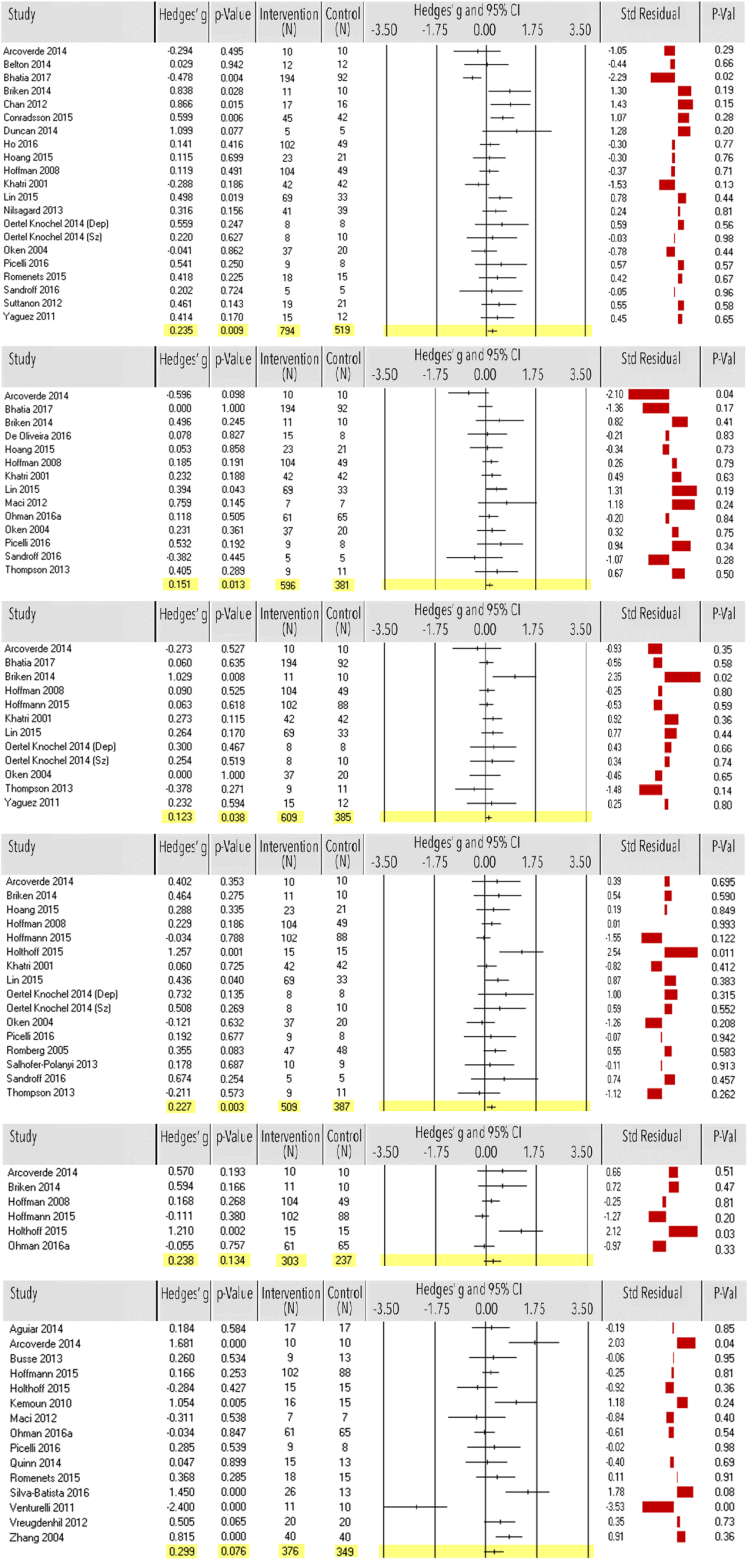
Meta-analysis of the effect of physical exercise on the cognitive domains (from top to down) attention and working memory, executive functioning, memory, psychomotor speed, verbal fluency and global cognition. Effect sizes (ES) per study and the overall ES are in Hedges’ *g* with corresponding *p* values and sample size of the intervention and control group. Standardized residual *z* scores of ES were used to detect outlier studies

Exercise showed a significant small effect on attention and working memory (*k* = 21, *n* = 1313, ES = 0.24, 95% CI 0.06–0.41, *p* = 0.009; Fig. 4) with moderate heterogeneity [*Q*(20) = 40.83, *p* = 0.004; *I*^2^ = 51%]. Eight (40%) out of 20 studies comprised AD, HD or PD. The funnel plot and Egger’s test indicated potential publication bias [*t*(19) = 2.14, *p = 0*.046, *N*_R_ = 55] (Table 1). The ES remained significant after excluding one outlier study [219], four small studies (*n* < 20) [163, 181, 190, 225] and one study [193] with high risk of bias (*k* = 14, *n* = 923, ES = 0.25, 95% CI 0.08–0.42, *p* = 0.004). Heterogeneity turned low to moderate [*Q*(13) = 20.83, *p* = 0.076; *I*^2^ = 38%]. Egger’s test was non-significant (Table 1).

#### Executive functioning

Fourteen studies (*n* = 977) showed a significant small effect of exercise on executive functioning (ES = 0.15, 95% CI 0.03–0.27, *p* = 0.013; Fig. 4). Five (35.7%) out of 14 studies investigated physical exercise in AD, HD or PD. Studies were homogenous [*Q*(13) = 12.30, *p* = 0.503; *I*^2^ = 0%]. Egger’s test was non-significant (Table 1). After excluding one outlier [63] and three small studies [68, 163, 190], ES remained significant (*k* = 10, *n* = 916, ES = 0.17, 95% CI 0.04–0.29, *p* = 0.009). There were no studies with high risk of bias.

#### Memory

Twelve studies (*n* = 994) examined the effect of physical exercise on memory and showed a beneficial small effect of exercise (involving mainly aerobic exercise) (ES = 0.12, 95% CI 0.07–0.24, *p* = 0.038; Fig. 4). Four (33.3%) out of 2 studies comprised AD, HD or PD. Studies were homogenous [*Q*(11) = 10.74, *p* = 0.465; *I*^2^ =  0%]. Egger’s test was non-significant (Table 1). After excluding one outlier study [128] and one small study [225], ES was non-significant (*k* = 9, *n* = 939, ES = 0.09, 95% CI − 0.03 to 0.21, *p* = 0.127), while studies remained homogenous (Table 1).

#### Psychomotor speed

Exercise showed a significant small effect on psychomotor speed (*k* = 16, *n* = 896, ES = 0.23, 95% CI 0.08 to 0.38, *p* = 0.003; Fig. 4). Five (31.3%) out of 16 studies were based on AD, HD or PD. Heterogeneity among studies was low [*Q*(15) = 19.02, *p* = 0.213; *I*^2^ = 21%]. Funnel plot and Egger’s test indicated potential publication bias [*t*(14) = 2.36, *p* = 0.035, *N*_R_ = 42]. After excluding one outlier [65] and four small studies [162, 163, 190, 225], ES remained significant (*k* = 10, *n* = 786, ES = 0.14, 95% CI 0.005–0.27, *p* = 0.042). Studies showed complete homogeneity and Egger’s test was non-significant (Table 1).

#### Verbal fluency

Exercise showed no significant effect on verbal fluency (*k* = 6, *n* = 540, ES = 0.24, 95% CI − 0.07 to 0.55, *p* = 0.134; Fig. 4) and remained non-significant after excluding one outlier study [65] (*k* = 5, *n* = 510, ES = 0.06, 95% CI − 0.15 to 0.27, *p* = 0.569). Four (66.7%) out of six studies comprised AD, HD or PD. Heterogeneity among studies was moderate to high [*Q*(5) = 14.36, *p* = 0.014; *I*^2^ = 65%; Table 1] but decreased after excluding the outlier (Table 1).

#### Global cognition

Fifteen studies (*n* = 725), all comprising AD, HD or PD, showed a trend of exercise in improving global cognition (ES = 0.30, 95% CI − 0.03 to 0.63, *p* = 0.076; Fig. 4). ES increased and showed significance (*k* = 10, *n* = 620, ES = 0.39, 95% CI 0.09–0.68, *p* = 0.010) after excluding two outliers [63, 74], three small studies [68, 119, 190] and one study [193] with high risk of bias. Heterogeneity was high [*Q*(14) = 60.79, *p* < 0.0001; *I*^2^ = 77%] but decreased after exclusion of the studies [*Q*(9) = 26.15, *p* = 0.002; *I*^2^ = 66%]. Egger’s test was non-significant (Table 1).

Separate analyses per disorder showed beneficial effects of exercise on A and WM in PD, PS in Sz and on GC in AD and PD (Table 2).

The study by Oertel Knöchel et al. [105] and Maci et al. [68] investigated physical exercise in combination with a cognitive intervention. Exclusion of these studies did not change results for any of the outcome measures.

### Studies with ITT-analysis

Additional analyses with studies with only low or unclear risk of bias on ITT analyses showed even larger effect of exercise on both QoL (ES = 0.56) and depressive symptoms (ES = 0.90), while effect on the cognitive domain psychomotor speed remained small (ES = 0.24) but significant. Effect of physical exercise on all the other cognitive domains was no longer significant. See Online Resource 6 for a detailed overview of these results.

### Moderator analysis

Subgroup analysis showed a significant medium effect of aerobic and neuromotor exercise and a medium-to-large effect of resistance exercise on QoL and depressive symptoms. Furthermore, a comprehensive program including all types of exercises according to ACSM was also effective in improving QoL. For cognition, aerobic and neuromotor exercises showed significant effects (Table 1).

Meta-regression analysis showed a small but positive dose–response effect for the amount of weekly exercise in min/week in reducing depressive symptoms (*β* = 0.007, 95% CI 0.002–0.013, *p* = 0.012; Online Resource 7–8), indicating that every 1-min increase in exercise intervention per week corresponds to an 0.007 unit increase is ES. No significant effect was found for the moderator total length of intervention (range 1.4–104 weeks). Additional meta-regression results are shown in Online Resource 7.

#### Intensity

With regard to intensity of the exercise intervention as possible moderator, 50 of the analyzed studies (41.0%) did not report any information. Of the remaining 59.0%, 18 studies (25.0%) investigated neuromotor exercises and therefore possibly could not report any intensity level. 36 studies (50.0%) applied low-to-moderate intensity of exercise, while 16 studies (22.2%) investigated moderate-to-high intensity exercise. Two studies (2.8%) investigated low-to-high intensity exercise (Online Resource 9).

#### Safety

Sixty-five studies (53.3%) reported on safety aspects of the exercise intervention (Online Resource 10). Forty-five of these studies (69.2%) found no physical injuries related to exercise. Eighteen studies (27.7%) found physical injuries that were related to the exercise intervention. These consisted mainly of muscle/joint pain (17.5%), fall incidents (11.4%, all with complete recovery) and ankle sprain (1.9%). In 83.3% of these studies (*k* = 15), physical injuries were short-lasting and/or had no consequences for participation in and completion of the exercise intervention.

## Discussion

One hundred and twenty-two studies, including 7231 patients, showed a significant medium-size effect (ES = 0.40) of exercise as an add-on therapeutic intervention on QoL (*k* = 64, *n* = 4334), a large effect (ES = 0.78) on depressive symptoms (*k* = 60, *n* = 2909) and a small but significant effect (ES = 0.12–0.24) on improving function in several cognitive domains. The effects for QoL and depression were well powered. The included number of patients was lower for cognition (*k* = 36, *n* = 2125), which makes these results more sensitive for new findings. From the studies that reported on safety (*k* = 18), low incidences of complications related to the exercise interventions were found, which had no lasting consequences for participation in and completion of the exercise interventions.

### Current clinical practice

In present clinical practice, the role of physical exercise as an add-on therapy in the management of QoL, depressive symptoms and cognitive impairment in chronic brain disorders remains elusive [226–228]. Management guidelines sometimes suggest physical exercise in treatment of, e.g., physical health, motor symptoms, falls and fatigue in chronic brain disorders but lack in clarity over the effectiveness of physical exercise on the studied symptoms [229–235].

Chronic brain disorders commonly affect well being and QoL. Therefore, improvement of QoL is a main care objective in these disorders. Depressed mood and cognitive inabilities are important contributors to reduce QoL. Currently, evidence for treatment designed specifically to target QoL is lacking. Most treatments for chronic brain disorders alleviate disease-specific symptoms, progression or relapse. In contrast, exercise therapy targets overall well-being, mood and cognition, independent of type of disease.

At present, physical exercise is not generally viewed as an effective intervention. For example, in a recent review, Kok et al. evaluated treatment of depression in older adults and stated that depressive symptoms can be effectively treated with antidepressants whereas physical exercise may not be a mainstream treatment modality, yet might be considered as a complementary therapy [236]. In contrast, Turner et al., showed that the efficacy of antidepressants is subject to selective publication of positive studies with a precipitous drop in ES to an overall ES of 0.32 when non-published FDA approved drug trials of antidepressants were combined with published drug trials [237].

For dementia, there are still no disease-modifying agents available and treatment is limited to amelioration of symptoms [238]. The effects for cognition found in our meta-analysis are statistically small but significant and similar or larger than effects of cognitive therapy [239–244] or drug treatment [245–248], which makes these effects relevant for cognitive outcomes.

### Heterogeneity and moderators

To our knowledge, this is the first meta-analysis to assess the effect of physical exercise interventions across chronic brain disorders. Since heterogeneity between studies is a valid reason of concern in meta-analyses, our study shows that when we consider brain disorders to share underlying mechanisms, it is feasible to combine disorders and studies across disorders in a joint analysis. We found lower heterogeneities in the joint analysis compared to within-disorder analysis. High heterogeneity across studies and disorders was accounted for using the random-effects model and excluding outlier studies, small studies and studies with high risk of bias. As a consequence, for QoL and depressive symptoms, both heterogeneity and ES decreased, but exercise still showed a significant medium effect. Moderator analyses, performed to assess potential sources of heterogeneity, showed moderate variability between studies that investigated aerobic exercises whereas studies that evaluated the efficacy of resistance or neuromotor exercises on QoL and depressive symptoms showed higher ES and no heterogeneity. Largest effects were found for resistance exercise. Better performance of resistance exercise on these outcomes might be mediated by an increase in peripheral blood levels of Insulin-growth-factor-1 (IGF-1), which can cross the blood–brain barrier and has been shown to regulate the effects of exercise on depression, learning, angiogenesis and hippocampal neurogenesis [249, 250]. As one study evaluated the role of resistance exercise only on cognition, this result should be interpreted with caution. Heterogeneity across studies assessing cognition was low or completely lacking for all but two cognitive domains (i.e., attention and working memory and global cognition) that showed significant results. For cognition, neuromotor exercise resulted in higher effects than aerobic exercise. Neuromotor exercises involve multifaceted exercises that target different brain systems involved in the regulation of attention, balance, coordination, mood, motor functioning and cognition, amongst others. Hence, neuromotor exercises are suggested to improve synchronization between different brain areas, which might explain their efficacy on a wide variety of clinical symptoms [251].

We found a positive dose–response effect for the weekly time spent on exercise in min/week in reducing depressive symptoms, indicating that the more time spent on exercise per week, the larger the reduction in depressive symptoms. However, no significant dose–response effect was found for the total length of the exercise intervention (i.e., the number of weeks spend on exercise), suggesting that both short- and long-term exercise interventions might be beneficial in improving QoL, depressive symptoms, and cognition. Patient groups ranged in mean age from 15.4 to 84.0 years, but no significant effect of this moderator was found on the outcome measures indicating that the effect of exercise on the examined outcome measure is not age-dependent.

Regarding exercise intensity, most of the studies that provided information on the intensity of the studied exercise intervention, applied moderate exercise intensity. Additionally, we found that risk of possible complications due to exercise is low, which should not be considered a limiting factor for exercise intervention.

While all aforementioned moderators were expected to be an explanatory factor for the high heterogeneity in QoL, depressive symptoms and the cognitive domain global cognition, the role of exercise intensity and safety could not be assessed quantitatively. One other explanation for the high heterogeneity could be the different questionnaires used in the separate studies. For both QoL and depressive symptoms, 13 different rating scales were used. For global cognition, six different tests were used.

### Implications for clinical practice

Currently, physical exercise is not a standard part of the treatment of the six chronic brain disorders included in this study. Based on our work, it is likely that patients with any of the investigated brain disorders could benefit from additional physical exercise therapy. As safety issues and age constraints do not seem to be a limiting factor, healthcare professionals could use the present findings to provide patients with a tailored intervention in terms of type of exercise, exercise time and duration of intervention period. We showed a positive dose–effect interaction for exercise time, indicating that longer exercise programs are better for mood improvement. Most studies included in our meta-analysis assessed supervised exercise. Therefore, our results cannot be generalized to unsupervised exercise.

### Implications for further research

Given the purpose and transdiagnostic character of the present study, we chose to compare exercise intervention only to TAU control condition. Evaluation of any differential effects of other components of the interventions such as adherence, setting (e.g., home-based vs. gym-based), monitoring of exercise sessions with instruments (e.g., heart rate meters), cost-effectiveness and comparison with other control groups (e.g., active control conditions) is required to provide detailed recommendations on physical exercise interventions for the clinical practice.

### Strengths and limitations

The greatest strength of the present study is that it provides an up-to-date and extensive quantitative overview of the literature regarding the efficacy of different exercise interventions in patients with chronic brain disorders. Second, our findings are largely in accordance with previous (quantitative) reviews that synthesized evidence on the efficacy of physical exercise in the studied brain disorders [20, 22, 24, 25, 28, 252]. However, in contrast to previous work, we performed both transdiagnostic and within-disorder analyses and evaluated the effect of several moderators providing evidence that physical exercise can be considered as an effective add-on and transdiagnostic treatment.

This study has some limitations. First, several studies could not be included in the cognitive meta-analyses, so that the overall effect of exercise on cognition was based on fewer studies than the other meta-analyses, making these findings more susceptible to change over time (when more studies become available). Notably, a recent RCT of 4-month aerobic and resistance exercise of moderate to high intensity added to usual care found that physical exercise did not slow cognitive decline in patients with mild-to-moderate dementia [18]. The authors measured global cognition with Alzheimer’s disease assessment scale-cognitive subscale (ADAS-cog) and found a small average difference with uncertain clinical relevance. This study did not fulfill the inclusion criteria of our study to be included in the quantitative review. However, considering the fact that we included four RCTs [65, 68, 74, 224] with negative outcomes of exercise on global cognition in AD (see Fig. 4) and did not find a significant overall effect of exercise on global cognition, we do not expect that adding this study would have changed our findings. Second, the analysis regarding the effect of physical exercise on depressive symptoms included studies with different disorders, and the included studies also differed in the severity of depression, ranging from mild depression to the presence of major depressive disorder. This might have biased the findings and resulted in a high effect size. However, both low and high effect sizes were found in mild and major depression, which suggests that physical exercise is effective for depressive symptoms in general, irrespective of the underlying severity. Third, publication bias is an important possible drawback in meta-analytical studies. Egger’s test showed potential publication bias for QoL and depressive symptoms. However, the fail-safe numbers of these tests were extremely large, increasing the validity of the results. Fourth, heterogeneity among studies was high, possibly due to combining studies with largely different interventions offered to different groups. However, heterogeneity values of the joint analysis were lower than the within-disorder heterogeneities (Tables 1, 2), indicating consistency in studies across disorders so that joint analysis of disorders deemed sensible. Moreover, one of the main inter-study differing variables, age, did not affect the efficacy of exercise on the outcome measures. Besides, *Q*- and *I*^2^-statistic cannot be used to estimate the magnitude of true dispersion [253]. Fifth, for all outcome measures, the risk of bias assessment indicated highest risk in terms of attrition. Incomplete outcome data and lack of ITT-analysis in studies could have biased the observed results. However, to account for possible attrition bias, we performed separate analyses on studies that performed ITT-analysis and thus had low risk of bias and studies with unclear risk of bias on ITT analysis (i.e., insufficient information to judge). These results showed even higher effects of exercise on QoL and depressive symptoms, while effects on cognition remained similar for the cognitive domain PS, but turned to non-significance for the cognitive domains A and WM, EF and M. The latter is likely due to the moderate to high heterogeneity among studies after inclusion of the study by [219]. Finally, we randomly selected six brain disorders of various etiology (e.g., neurodegenerative, neurodevelopmental, inflammatory) to demonstrate the generalizability of efficacy of exercise. Since we did not find any RCTs evaluating the effect of physical exercise in bipolar disorder, we decided to only include unipolar depression in the present study. Other brain disorders, such as epilepsy, traumatic brain injury and migraine have been investigated as well, but given restriction in time and capacity (as well as wordcount), this paper was confined to the chronic brain disorders summed above.

## Conclusion

Additional therapy with physical exercise in patients with chronic brain disorders seems safe and has a medium-sized effect on QoL and a large beneficial effect on depressive symptoms, with a positive dose–response correlation. The evidence for the efficacy on cognition is small, but clinically relevant. Therefore, to improve the health status of patients with chronic brain disorders, add-on exercise therapy should be considered as an essential part of the treatment.

## Electronic supplementary material

Below is the link to the electronic supplementary material.

Supplementary file1 (PDF 87 kb)

Supplementary file2 (PDF 99 kb)

Supplementary file3 (PDF 595 kb)

Supplementary file4 (PDF 826 kb)

Supplementary file5 (PDF 329 kb)

Supplementary file6 (PDF 112 kb)

Supplementary file7 (PDF 98 kb)

Supplementary file8 (PDF 116 kb)

Supplementary file9 (PDF 105 kb)

Supplementary file10 (PDF 110 kb)

Supplementary file11 (PDF 82 kb)

Supplementary file12 (PDF 148 kb)

Supplementary file13 (PDF 147 kb)

Supplementary file14 (PDF 182 kb)
