# Supplementary material for: Physical exercise improves quality of life, depressive symptoms, and cognition across chronic brain disorders: a transdiagnostic systematic review and meta-analysis of randomized controlled trials
Source: J Neurol. 2019 Aug 14;268(4):1222–46. doi: 10.1007/s00415-019-09493-9 (PMC7990819; doi:10.1007/s00415-019-09493-9)
Supplement: Supplementary file 1 — Supplementary file1 (PDF 87 kb) [file 415_2019_9493_MOESM1_ESM.pdf]

Physical exercise improves quality of life, depressive symptoms, and cognition across chronic brain disorders: a transdiagnostic systematic review and meta-analysis of randomized controlled trials

Meenakshi Dauwan*, Marieke JH Begemann, Margot IE Slot, Edwin HM Lee, Philip Scheltens, Iris EC Sommer

*** Corresponding author:**
Meenakshi Dauwan, M.D.
Neuroimaging Center, University Medical Center Groningen
Department of Clinical Neurophysiology and MEG Center, Amsterdam UMC, Vrije Universiteit Amsterdam
Department of Psychiatry, University Medical Center Utrecht
Neuroimaging Center 3111
Antonius Deusinglaan 2
9713 AW Groningen, The Netherlands
Tel: +31 88 75 57468
E-mail: m.dauwan@umcg.nl; m.dauwan-3@umcutrecht.nl

Table S1: Systematic search strategy for two out of four used databases for one disorder

**PUBMED**
**#1: Domain**<br>(Alzheimer[Title/Abstract] OR AD[Title/Abstract])
**#2: Determinant**<br>(Exercise[Title/Abstract] OR physical[Title/Abstract] OR training[Title/Abstract] OR endurance[Title/Abstract] OR aerobic[Title/Abstract] OR anaerobic[Title/Abstract] OR resistance[Title/Abstract] OR sport[Title/Abstract] OR yoga[Title/Abstract])
**#3: #1 AND #2**
**EMBASE**
**#1: Domain**<br>(Alzheimer OR AD):ab,ti
**#2: Determinant**<br>(Exercise OR physical OR training OR endurance OR aerobic OR anaerobic OR resistance OR sport OR yoga):ab,ti
**#3: #1 AND #2**
