# Supplementary material for: Physical exercise improves quality of life, depressive symptoms, and cognition across chronic brain disorders: a transdiagnostic systematic review and meta-analysis of randomized controlled trials
Source: J Neurol. 2019 Aug 14;268(4):1222–46. doi: 10.1007/s00415-019-09493-9 (PMC7990819; doi:10.1007/s00415-019-09493-9)
Supplement: Supplementary file 2 — Supplementary file2 (PDF 99 kb) [file 415_2019_9493_MOESM2_ESM.pdf]

**Physical exercise improves quality of life, depressive symptoms, and cognition across chronic brain disorders: a transdiagnostic systematic review and meta-analysis of randomized controlled trials**

Meenakshi Dauwan*, Marieke JH Begemann, Margot IE Slot, Edwin HM Lee, Philip Scheltens, Iris EC Sommer

*** Corresponding author:**
Meenakshi Dauwan, M.D.
Neuroimaging Center, University Medical Center Groningen
Department of Clinical Neurophysiology and MEG Center, Amsterdam UMC, Vrije Universiteit Amsterdam
Department of Psychiatry, University Medical Center Utrecht
Neuroimaging Center 3111
Antonius Deusinglaan 2
9713 AW Groningen, The Netherlands
Tel: +31 88 75 57468
E-mail: m.dauwan@umcg.nl; m.dauwan-3@umcutrecht.nl

**Table S2: Cognitive domains classified based on the cognitive domains and/or cognitive tests investigated across studies and disorders**

| **Attention & Working Memory** |
|---|
| 'Alertness' and 'Shift of attention' subtests of computerized ' Test Battery of Attention' |
| Digit Span forward |
| Digit Vigilance Test |
| Dual-task Timed Up and Go (TUG) |
| Letter Cancellation Test |
| Memory with Interference test |
| Modified Flanker task |
| Rapid visual information processing |
| Spatial working memory |
| Useful Field of View task |
| Wechsler Memory Scale (WMS)-III: Working Memory: Spatial Span & Letter-Number-Span |
| Working memory for patterns |

| **Executive Functioning** |
|---|
| Clock Drawing Test |
| Delis-Kaplan Executive Function System |
| Digit Span backward |
| Frontal Assessment Battery |
| Raven colored progressive matrices |
| Ruff 2 & 7 test |
| Stroop test (congruent score) |
| Subset 3 of 'Achievement Testing System' (Leistungsprüfsystem) |
| Trail Making Test (TMT) Part B |
| TMT-B minus TMT-A |
| Wisconsin Card Sorting test |

| **Memory** |
|---|
| Alzheimer's Disease Assessment Scale – Cognitive Subscale (ADAS-Cog) verbal memory test |
| Brief Visuospatial Memory Test-Revised |

| |
| --- |
| Hong Kong List Learning Test (HKLLT) |
| Hopkins Verbal Learning Test-Revised |
| Paired Associate Learning |
| Pattern Recognition Memory |
| Rey Auditory Verbal Learning test |
| Verbal Learning and Memory test |
| WMS Logical Memory subtest |
| WMS Verbal Paired Associates subtest |
| WMS Visual Reproduction Subtest |
| **Psychomotor Speed** |
| Brief Assessment of cognition in Schizophrenia (BACS): Symbol Coding + category fluency: animal naming (1 score) |
| FETZ-test |
| Paced Auditory Serial Addition Test (PASAT) |
| Stroop test (incongruent score) |
| Symbol Digit Modalities Test (SDMT) |
| TMT-A |
| Wechsler Adult Intelligence Scale-Revised (WAIS-R) Digit Symbol Subtest |
| **Verbal Fluency** |
| Controlled Oral Word Association test |
| Regensburg Verbal Fluency test |
| Semantic Word Fluency with CERAD and FAS-test |
| Verbal Fluency test (animal category) |
| **Global Cognition** |
| ADAS-Cog |
| Cambridge Cognitive Examination (CAMCOG) |
| French ERFC (Rapid Evaluation of Cognitive Function) |
| Mini Mental State Examination (MMSE) |
| Montreal Cognitive Assessment (MoCA) |
| Unified Huntington’s Disease Rating Scale (UHDRS) cognitive scale |
