# Supplementary material for: Physical exercise improves quality of life, depressive symptoms, and cognition across chronic brain disorders: a transdiagnostic systematic review and meta-analysis of randomized controlled trials
Source: J Neurol. 2019 Aug 14;268(4):1222–46. doi: 10.1007/s00415-019-09493-9 (PMC7990819; doi:10.1007/s00415-019-09493-9)
Supplement: Supplementary file 3 — Supplementary file3 (PDF 595 kb) [file 415_2019_9493_MOESM3_ESM.pdf]

Physical exercise improves quality of life, depressive symptoms, and cognition across chronic brain disorders: a transdiagnostic systematic review and meta-analysis of randomized controlled trials

Meenakshi Dauwan*, Marieke JH Begemann, Margot IE Slot, Edwin HM Lee, Philip Scheltens, Iris EC Sommer

*** Corresponding author:**
Meenakshi Dauwan, M.D.
Neuroimaging Center, University Medical Center Groningen
Department of Clinical Neurophysiology and MEG Center, Amsterdam UMC, Vrije Universiteit Amsterdam
Department of Psychiatry, University Medical Center Utrecht
Neuroimaging Center 3111
Antonius Deusinglaan 2
9713 AW Groningen, The Netherlands
Tel: +31 88 75 57468
E-mail: m.dauwan@umcg.nl; m.dauwan-3@umcutrecht.nl

Supplementary text

Formulas used to compute effect sizes
The effect sizes have been calculated in Comprehensive Meta-Analysis according to the following formula (Hedges 1981, Distribution theory for Glass's estimator of effect size and related estimators. Journal of Educational Statistics 6, 107-128):

$$d = \frac{\bar{x}_1 - \bar{x}_2}{s*}$$

with d = effect size, x_1 and x_2 = sample means and s* = pooled standard deviation, computed as:

$$s* = \sqrt{\frac{(n_1 - 1)s_1^2 + (n_2 - 1)s_2^2}{n_1 + n_2 - 2}}$$

with n_1 and n_2 the two sample sizes s_1^2 and s_2^2 the estimated population variances of both groups

Unbiased effect size estimated (Hedges' g) =

$$g = J \times d$$

with J computed as:

$$J = 1 - \frac{3}{4df - 1}$$

J = correction factor used to convert d to Hedges' g with df = degrees of freedom used to estimate the pooled standard deviation which for two independent groups is $n_1 + n_2 - 2$.

Two examples of how Comprehensive Meta-Analysis computes Hedges' g are shown below:

Example 1: when pre- and post-mean data was available from studies:

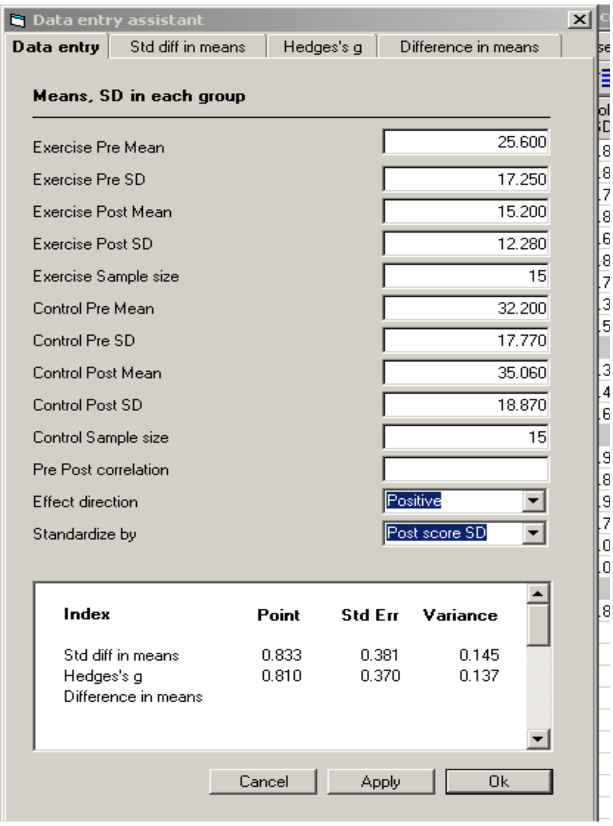

Standardized difference in means

Starting with
Mean change, SD difference, N, in each group, Pre/Post Corr

Standardized difference in means
** Option to standardize by Post SD **

MeanChange(1) = Group 1 mean difference
MeanChange(2) = Group 2 mean difference
RawDiff = MeanChange(1) - MeanChange(2)

SDPost(1) = Given
SDPost(2) = Given

SDPostPooled = Sqr(((n(1) - 1) * SDPost(1) ^ 2 + (n(2) - 1) * SDPost(2) ^ 2) / (n(1) + n(2) - 2))

StdPostDiff = RawDiff / SDPostPooled
StdPostDiffSE = Sqr((1 / n(1) + 1 / n(2)) + StdPostDiff ^ 2 / (2 * (n(1) + n(2))))

RawDiff = -10.400 - 2.860 = -13.260
SDPost(1) = 12.280
SDPost(2) = 18.870
SDPostPooled = Sqr(((15 - 1) * 12.280 ^ 2 + (15 - 1) * 18.870 ^ 2) / (15 + 15 - 2)) = 15.920

StdPostDiff = -13.260 / 15.920 = -0.833
StdPostDiffSE = Sqr((1 / 15 + 1 / 15) + -0.833 ^ 2 / (2 * (15 + 15))) = 0.381

Hedges' g
Standardized mean difference corrected for bias (Hedges' g)

The program computes the Standardized mean difference (d) and then multiplies d by a correction factor (J) to compute g.

Correction factor J
J = 1 - (3 / (4 * df - 1))
Where df = NTot - 2
J = 1 - (3 / (4 * 28 - 1)) = 0.973

Computation of g
g = d * J
StdErr(g) = StdErr(d) * J
Variance(g) = StdErr(g) ^ 2

g = 0.833 * 0.973 = 0.810
StdErr(g) = 0.381 * 0.973 = 0.370
Variance(g) = 0.370 ^ 2 = 0.137

Example 2: when mean change data was available from studies:

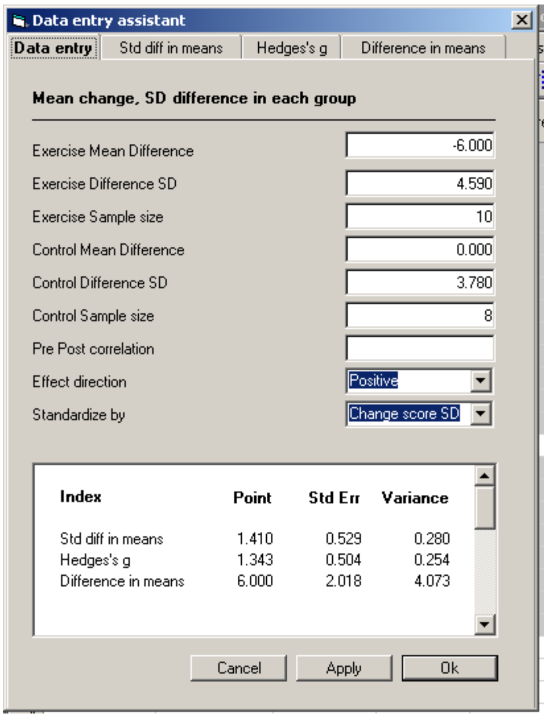

Standardized difference in means

Starting with
Mean change, SD difference, N, in each group, Pre/Post Corr

Standardized difference in means
** Option to standardize by Change SD **

RawDiff = MeanChange(1) - MeanChange(2)
SDChangePooled = Sqr((((n(1) - 1) * SDChange(1) ^ 2 + (n(2) - 1) * SDChange(2) ^ 2) / (n(1) + n(2) - 2)))

StdChangeDiff = RawDiff / SDChangePooled
StdChangeDiffSE = Sqr(1 / n(1) + 1 / n(2) + StdChangeDiff ^ 2 / (2 * (n(1) + n(2))))

RawDiff = -6.000 - 0.000 = -6.000
SDChangePooled = Sqr((((10 - 1) * 4.590 ^ 2 + (8 - 1) * 3.780 ^ 2) / (10 + 8 - 2))) = 4.255

StdChangeDiff = -6.000 / 4.255 = -1.410
StdChangeDiffSE = Sqr(1 / 10 + 1 / 8 + -1.410 ^ 2 / (2 * (10 + 8))) = 0.529

Hedges' g

Standardized mean difference corrected for bias (Hedges' g)

The program computes the Standardized mean difference (d) and then multiplies d by a correction factor (J) to compute g.

Correction factor J
J = 1 - (3 / (4 * df - 1))

Where df = NTot - 2
J = 1 - (3 / (4 * 16 - 1)) = 0.952

Computation of g
g = d * J
StdErr(g) = StdErr(d) * J
Variance(g) = StdErr(g) ^ 2

g = 1.410 * 0.952 = 1.343
StdErr(g) = 0.529 * 0.952 = 0.504
Variance(g) = 0.504 ^ 2 = 0.254

Acknowledgement
The authors gratefully acknowledge the corresponding authors of the studies that provided additional information upon request:

Prof. Dr. Cynthia L. Comella; Dr. Erin R. Foster; Prof. Dr. Steen G. Hasselbalch; Prof. Dr. Linda Tickle-Degnen; Dr. Victoria Goodwin; Dr. Patricia A. Kinser; Prof. Dr. C.M. Wiles; Dr. Elaine H. Coulter; Dr. Lorna Paul; Dr. C.C. Harro; Dr. Mark B. Andersen, Dr. U. Dalgas, Dr. H. Negahban; Dr. D. Veale; Dr. T. Carter; Dr. F. Schuch; Prof. Dr. O. P. Almeida; Dr. B. Sangelaji; Dear dr. Martiny; Dr. M. Niemi; Dr. A. Vreugdenhil; Prof. Dr. M. Busse; Dr. D.K. Lee; Prof. Dr. C. Dettmers; Prof. Dr. D. T. Wade; Dr. M. Steinberg; Prof. Dr. A. Chetta; Dr. A. Solari; Dr. M.G. Ceravolo; Dr. A. Park; Dr. L.M. Shulman; Dr. E. Eftekhari; Dr. M. Morris; Dr. M. Shahidi; DR. M. Kelson; Prof. Dr. M.R. Ziman; Prof. Dr. B.S. Oken; Dr. T. Cruickshank; Dr. M. Danoudis; Dr. J. Krogh; Dr. C. Heesen; Dr. S.M. Gold; Dr. P. Suttanon; Prof. Dr. A. Ashburn; Dr. F. Patti; Dr. L. Teri; Dr. K. Dashtipour; Dr. P. Hoang; Prof. Dr. S. Lord; Dr. R. Machado-Vieira; Dr. M. Kim; Dr. C.C. Siqueira
