# Supplementary material for: Physical exercise improves quality of life, depressive symptoms, and cognition across chronic brain disorders: a transdiagnostic systematic review and meta-analysis of randomized controlled trials
Source: J Neurol. 2019 Aug 14;268(4):1222–46. doi: 10.1007/s00415-019-09493-9 (PMC7990819; doi:10.1007/s00415-019-09493-9)
Supplement: Supplementary file 4 — Supplementary file4 (PDF 826 kb) [file 415_2019_9493_MOESM4_ESM.pdf]

Physical exercise improves quality of life, depressive symptoms, and cognition across chronic brain disorders: a transdiagnostic systematic review and meta-analysis of randomized controlled trials

Meenakshi Dauwan*, Marieke JH Begemann, Margot IE Slot, Edwin HM Lee, Philip Scheltens, Iris EC Sommer

*** Corresponding author:**
Meenakshi Dauwan, M.D.
Neuroimaging Center, University Medical Center Groningen
Department of Clinical Neurophysiology and MEG Center, Amsterdam UMC, Vrije Universiteit Amsterdam
Department of Psychiatry, University Medical Center Utrecht
Neuroimaging Center 3111
Antonius Deusinglaan 2
9713 AW Groningen, The Netherlands
Tel: +31 88 75 57468
E-mail: m.dauwan@umcg.nl; m.dauwan-3@umcutrecht.nl

Table S3: Descriptive overview of studies per disorder

Study	Age in years (mean or range)	Gender	Type of patient	Illness duration (mean in years)	Type of control (N)	Type of exercise (N)	Frequency	Study duration	Type of outcome
**Alzheimer's disease**									
Aguiar 2014	76.7	Both	AD	IG: 2.1<br>CG: 1.6	AD: rivastigmine (N=18)	Aerobic + strength exercise (N=22)	40min/day, 2x/week	6 months	- Cognition<br>- QoL<br>- ADL<br>- TUG
Arcoverde 2014	78.8	Both	AD	IG: 4.3<br>CG: 4.1	AD: usual care (N=10)	Treadmill walking (N=10)	30min/day, 2x/week	4 months	- Cognition<br>- BBS<br>- TUG<br>- STS
Cott 2002	82.0	Both	AD	-	- AD: talking (N=30)<br>- AD: usual care (N=26)	Walking (N=30)	30min/day, 5x/wk	16 weeks	- Communication<br>- 2MWT<br>- Functional status
Cox 2015	74.4	Both	AD	-	AD: usual care (N=-)	Home-based physical activity (N=-)	150min/week	6 months	- Physical activity<br>- Balance

									- Leg & grip strength<br>- Mobility
Friedman 1991	-	-	Probable AD	-	AD: conversation (N=-)	Walking (N=-)	30min/day, 3x/week	10 weeks	- Conversation performance
Hoffmann 2015	70.5	Both	Probable AD	-	AD: TAU (N=93)	Aerobic exercise (N=107)	60min/day, 3x/week	16 weeks	- Cognition<br>- HADS<br>- QoL<br>- NPI<br>- ADL
Holthoff 2015[30]	71.5	Both	Mild to moderate AD	-	AD: usual care (N=15)	Physical activity (N=15)	30min/day, 3x/wk, for 12 weeks	24 weeks	- ADL<br>- NPI<br>- Cognition
Kemoun 2010	82.0	Both	AD	-	AD: usual care (N=18)	Walking + exercise (N=20)	60min/day, 3x/wk	15 weeks	- Cognition<br>- Physical function
Kim 2016	81.5	Both	AD	-	AD: MCP (N=14)	Aerobic & flexibility exercise + MCP (N=19)	Exercise: 60min/day, 5x/week<br>MCP: 120min/day, 5x/week	6 months	- Cognition<br>- BBS<br>- Physical function
Lanza 2018	78.5	Both	AD	IG: 2.7<br>CG: 4.0	AD: physical activity (N=6)	Shiatsu + physical activity (N=6)	- Physical activity: 75/day, 3x/wk<br>- Shiatsu: 40min/wk	10 months	- Cognition<br>- Depression<br>- ADL
Lamb 2018	-	Both	AD, Vascular dementia	-	Dementia: usual care (N=-)	Aerobic and strength exercises (N=-)	60-90min/day, 2x/week + 60min/week at home, for 4 months	12 months	- Cognition<br>- EQ-5D<br>- NPI
Lautenschlager 2015[1]	74.4	Both	AD	-	AD: usual care (N=66)	Home-based physical activity (N=66)	150min/week	6 months	- Cognition<br>- QoL
Maci 2012	72.7	Both	AD	-	AD: usual care (N=7)	Aerobic exercise + cognitive stimulation + socialization (N=7)	- Aerobic exercise: 60min/day, 5x/week<br>- Cognitive stimulation: 60min/day, 5x/week<br>- Socialization: 30min/day, 5x/week	12 weeks	- Cognition<br>- CSDD<br>- QoL<br>- ADL<br>- Anxiety
McCurry 2011	81.0	Both	Probable or possible AD	IG: 4.5<br>CG (light therapy): 3.0<br>CG (combination): 3.0<br>CG (usual care): 4.0	- AD: light therapy (N=34)<br>- AD: usual care (N=33)	- Walking (N=32)<br>- Walking + light therapy (N=33)	3x60min in 8 weeks	6 months	- Sleep parameters
Morris2017[31]	72.9	Both	Probable AD	-	AD: stretching and tonic exercises (N=37)	AD: aerobic exercise (N=39)	60min/wk in week 1, increasing to 150min/wk	26 weeks	- Cognition<br>- Depression<br>- Cardiorespiratory fitness<br>- MRI

Ohman 2016a	78.0	Both	Probable AD	-	AD: usual care (N=70)	- Home-based exercise (N=70)<br>- Group-based exercise (N=70)	60min/day, 2x/week	1 year	- Cognition
Ohman 2016b	77.8	Both	Probable AD	-	AD: usual care (N=65)	Exercise (N=129)	60min/day, 2x/week	1 year	- Fall rate<br>- FIM
Padala 2012	80.5	Both	Mild AD	-	AD: Wii-Fit intervention (N=11)	Walking (N=11)	30min/day, 5x/week	8 weeks	- BBS<br>- TUG<br>- Tinetti test<br>- ADL<br>- QoL<br>- Cognition
Pedrinolla 2018	-	-	AD	-	AD: cognitive training (N=-)	Exercise (N=16)	-	-	- Gait
Perea 2015	-	-	AD	-	AD: stretching (N=14)	Treadmill walking (N=16)	-	26 weeks	- MRI Thalamo-cortical connectivity
Pitkala 2013[2]	78.0	Both	Probable AD	-	- AD: usual care (N=70)	- Home-based exercise (N=70)<br>- Group-based exercise (N=70)	60min/day, 2x/week	1 year	- FIM<br>- Mobility
Roach 2011	88.2	Both	Probable or possible AD	-	AD: social conversation (N=25)	- Walking (N=29)<br>- Strength, balance & endurance exercise (N=28)	30min/day, 5x/wk	16 weeks	- 6MWT<br>- Cognition<br>- ACIF
Rolland 2007	83.0	Both	Probable or possible AD	-	AD: usual care (N=67)	Aerobic + strength exercise (N=67)	60min/day, 2x/wk	12 months	- ADL<br>- 6MWT<br>- One-leg balance<br>- Nutritional status<br>- NPI<br>- Depression
Santana-Sosa 2008	74.5	Both	AD	-	AD: usual care (N=8)	Resistance + coordination exercises (N=8)	75min/day, 3x/week	12 weeks	- ADL<br>- Barthel index<br>- Functional capacity<br>- Tinetti test
Sopina 2017[30]	70.5	Both	AD	-	AD: TAU (N=93)	Aerobic exercise (N=107)	60min/day, 3x/week	16 weeks	- Cost-effectiveness
Steen Jensen 2016[30]	68.7	Both	AD	IG: 1.1<br>CG: 1.5	AD: TAU (N=27)	Aerobic exercise (N=26)	60min/day, 3x/wk	16 weeks	- CSF amyloid beta and tau
Steen Jensen 2017[30]	68.6	Both	Mild AD	IG: 1.1<br>CG: 1.7	AD: TAU (N=26)	Aerobic exercise (N=25)	60min/day, 3x/wk	16 weeks	- CSF NFL<br>- CSF Ng<br>- CSF YKL-40

Steinberg 2009	75.3	Both	Probable AD	-	AD: home safety assessment (N=13)	Aerobic, strength + balance exercise (N=14)	-	12 weeks	- CSF VILIP-1<br>- Cognition<br>- QoL<br>- Depression<br>- NPI<br>- Functional performance
Suttanon 2012	82.0	Both	AD	-	AD: education program (N=21)	Balance + strength home exercise (N=19)	5x/week	6 months	- Cognition<br>- Physical function
Tappen 2000	86.1	Both	Probable AD	-	AD: conversation (N=24)	- Walking (N=26)<br>- Walking + conversation (N=21)	30min/day, 3x/week	16 weeks	- 6MWT
Teri 2003	78.0	Both	Probable or possible AD	IG: 4.0<br>CG: 5.0	AD: usual care (N=77)	Aerobic, strength, balance exercise (N=76)	30min/day, 2x/week, for 3 months	24 months	- SF-36<br>- Depression<br>- Physical function
Van der Kleij 2018	68.5	Both	AD	-	AD: usual care (N=24)	Aerobic exercise (N=27)	60min/day, 3x/week	16 weeks	- MRI: cerebral blood flow
Venturelli 2011	84.0	Both	AD	-	AD: usual care (N=12)	Walking (N=12)	30min/day, 4x/week	24 weeks	- Cognition<br>- Barthel index<br>- Physical function<br>- 6MWT
Venturelli 2016	-	-	AD	-	- AD: TAU (N=20)<br>- AD: cognitive training (N=20)	- Aerobic exercise (N=20)<br>- Aerobic exercise + cognitive training (N=20)	60min/day, 5x/wk	12 weeks	- Cortisol levels<br>- NPI<br>- Agitation
Vidoni 2017[31]	72.6	Both	AD	-	AD: stretching and tonic exercises (N=32)	AD: aerobic exercise (N=33)	60min/wk in week 1, increasing to 150min/wk	26 weeks	- Functional disability<br>- Informal caregiving
Vreugdenhil 2012	74.1	Both	AD	IG: 4.2<br>CG: 3.8	AD: TAU (N=20)	Home-based exercises + walking (N=20)	At least 30min/day	4 months	- Cognition<br>- Physical function<br>- Depression<br>- ADL
Wong 2013	75.9	-	AD	-	AD: stretching exercises (N=7)	Tai Chi Chuan (N=9)	45min/day, 2x/week	12 weeks	- Cognition<br>- CDSS<br>- Balance
Yaguez 2011	73.1	Both	AD	-	AD: usual care (N=12)	Movement exercise (N=15)	90min/wk	6 weeks	- Cognition
Yang 2015	50-80	-	AD	-	AD: health education (N=25)	Cycling (N=25)	40min/day, 3x/week	12 weeks	- Cognition<br>- QoL<br>- Plasma APOE a1 levels<br>- NPI
Zhang 2004	-	-	AD	-	AD: usual care (N=40)	Rehabilitation (N=40)	-	12 months	- SF-36<br>- Cognition<br>- ADL

**Huntington's disease**									
Busse 2013	50.4	Both	HD	-	HD: usual care (N=15)	- Gym training<br>- Home walking (N=16)	- Gym: 30min/wk<br>- Walking: 10-30min/day, 2x/wk	24 weeks	- Retention<br>- Adherence<br>- Safety<br>- UHDRS (motor, cognition, functioning, independence)<br>- Gait parameters<br>- SF-36<br>- 6MWT<br>- Physical activity
Busse 2017	54.9	Both	HD	-	HD: social interaction (N=24)	Physical activity (N=22)	-	14 weeks	- UHDRS (motor and functioning)<br>- Physical activity<br>- 6MWT<br>- TUG<br>- Cognition<br>- EQ-5D
Khalil 2013	52.8	-	Early- to mid-stage HD	-	HD: usual care (N=12)	Home based exercise with DVD + Walking (N=13)	- Home exercise: 3x/wk<br>- Walking: 30min/wk	8 weeks	- Gait<br>- Balance<br>- Functional status<br>- Physical activity<br>- QoL<br>- UHDRS-motor
Quinn 2014	57.0	Both	HD	-	HD: usual care (N=15)	Task specific physical therapy (N=15)	± 60min/day, 2x/week, for 8 weeks	16 weeks	- UHDRS (motor, cognition, functioning)<br>- Adherence<br>- Retention<br>- BBS<br>- TUG<br>- HDQoL<br>- Physical function<br>- 10mWT<br>- 30CST<br>- HADS<br>- EQ-5D
Quinn 2016	52	Both	HD	-	HD: usual care (N=15)	Aerobic + resistance exercise (N=17)	50min/day, 3x/wk	12 weeks	- Safety<br>- Retention<br>- Cognition<br>- Motor function<br>- Physical fitness<br>- EQ-5D

Thompson 2013	53.0	-	Early to middle-stage HD	-	HD: usual care (N=11)	Multidisciplinary rehabilitation program (N=9)	- Group exercise: 60min/wk, 9 months<br>- Home exercise: 3x/wk, for 6 months<br>- Occupational therapy: 1x/2wk, for 6 months	9 months	- UHDRS motor<br>- Cognition<br>- Postural stability/balance<br>- BDI<br>- SF-36<br>- HDQoL
**Multiple Sclerosis**									
Afrasiabifar 2018	32.7	Both	MS	-	MS: TAU (N=25)	- Cawthorne-Cooksey balance exercise (N=25)<br>- Frenkel balance exercises (N=25)	60min/day, 3x/wk	12 weeks	- BBS
Aghaie 2010	-	Female	RR-MS	-	MS: wait-list (N=10)	- Aerobic training (N=10)<br>- Resistance training (N=10)	45min/day, 3x/week	8 weeks	- BBS<br>- FSS<br>- 10mWT<br>- 2MWT
Ahmadi 2010a	36.8	Female	MS; EDSS 2.3	IG: 5.6<br>CG: 5.0	MS: wait-list (N=10)	Treadmill training (N=10)	30min/day, 3x/week	8 weeks	- FSS<br>- MSQOL-54<br>- BBS<br>- 10mWT<br>- 2MWT
Ahmadi 2010b	34.4	Female	MS; EDSS 2.1	IG: 4.7<br>CG: 5.0	MS: wait-list (N=10)	Yoga therapy (N=11)	60-70min/day, 3x/week	8 weeks	- FSS<br>- MSQOL-54<br>- BBS<br>- 10mWT<br>- 2MWT
Ahmadi 2013[13]	35.2	Female	MS; EDSS 2.2	IG (treadmill): 5.6<br>IG (Yoga): 4.7<br>CG: 5.0	MS: wait-list (N=10)	- Treadmill training (N=10)<br>- Yoga therapy (N=11)	Treadmill: 50min/day, 3x/week<br>Yoga: 60-70min/day, 3x/week	8 weeks	- BDI<br>- BAI
Amiri 2018	31.6	Female	RR-MS; EDSS 2.5 – 5.5	-	MS: usual care (N=36)	Core stability training (N=36)	60min/day, 3x/wk	10 weeks	- Balance
Arazi 2016	28.5	Female	MS; EDSS1-4		MS: usual care (N=20)	Aerobic + resistance training (N=27)	55min/day, 3x/week	8 weeks	- Balance<br>- Fatigue<br>- 10mWT
Azimzadeh 2015	20-60	Female	RR-MS; EDSS ≤ 5.5	-	MS: usual care (N=18)	Tai Chi (N=18)	45-60min/day, 2x/week	12 weeks	- BBS
Barghi 2018	-	-	Chronic MS	-	MS: complementary and alternative medicine (N=10)	CIMT (N=10)	3,5h/day	10 days	- Motor function<br>- Structural MRI

Barrett 2009	54.4	Both	SP-MS with unilateral dropped foot	IG: 17.7<br>CG: 13.6	MS: functional peroneal nerve stimulation (N=26)	Physiotherapy home exercises (N=27)	30-60min/day, 7x/week	18 weeks	- 10mWT<br>- 3MWT
Bernhardt 2011	-	Both	MS; EDSS 0-6.5	-	MS: usual care (N=-)	- Gym training (N=-)	-	12 weeks	- Balance<br>- Spasticity<br>- Coordination
Bernhardt 2012	46.3	Both	MS; EDSS 3.2	-	MS: wait-list (N=-)	- PRT (N=-)<br>- Core & stability training (N=-)	60min/week	24 weeks	- BDI<br>- SF-36<br>- FSMC<br>- MSFC
Bjarnadottir 2007	37.4	Both	RR-MS; EDSS <4	IG: 8.7<br>CG: 8.3	MS: usual care (N=12)	Exercise (N=11)	60min/day, 3x/week	5 weeks	- SF-36<br>- Physical fitness<br>- Borg scale
Braendvik 2015	47.9	Both	MS; EDSS <6	IG: 8.3<br>CG: 6.2	MS: Strength training (N=15)	Treadmill training (N=14)	30min/day, 3x/week	8 weeks	- Balance<br>- Gait
Briken 2014[32]	49.8	Both	Progressive MS; EDSS 5.0	IG (arm): 17.1<br>IG (rowing): 14.1<br>IG: (bicycle): 13.3<br>CG: 18.9	MS: wait-list (N=11)	- Arm ergometry (N=12)<br>- Rowing (N=12)<br>- Bicycle ergometry (N=12)	15-45min/day, 2-3x/wk	10 weeks	- 6MWT<br>- Cognition<br>- Depression<br>- MFIS
Briken 2016[32]	50.2	Both	Progressive MS; EDSS 4.9	IG: 15.5<br>CG: 18.9	MS: wait-list (N=10)	Endurance exercise (N=32)	15-45min/day, 2-3x/wk	10 weeks	- BDNF<br>- IL-6<br>- Irisin
Broekmans 2010a	47.8	Both	MS; EDSS 4.3	-	- MS: usual care (N=14)	- Resistance training without electro-stimulation (N=11)<br>- Resistance training with electro-stimulation (N=11)	5x60min per 2 weeks	22 weeks	- Knee strength<br>- TUG<br>- T25FW<br>- 2MWT<br>- FR<br>- RMI
Broekmans 2010b	47.9	Both	MS; EDSS 4.3	-	- MS: usual care (N=14)	WBV exercise program (N=11)	50min per session	22 weeks	- Knee strength<br>- BBS<br>- TUG<br>- T25FW<br>- 2MWT
Bulguroglu 2015	-	-	MS; EDSS <4	-	MS: usual care (N=13)	- Mat pilates (N=12)<br>- Reformer pilates (N=13)	-	-	- BBS<br>- TUG<br>- ABC<br>- FSS<br>- FIS<br>- MSQOL-54
Cakit 2010	38.3	Both	RR-MS/SP-MS; EDSS ≤ 6.0	Resistance: 9.2<br>Home exercise: 6.2<br>CG: 6.6	- MS: usual care (N-15)	- Progressive resistance training (N=15)	90-95min/day, 2x/wk	8 weeks	- TUG<br>- DGI<br>- FR

						- Home-based exercises (N-15)			- FES<br>- FSS<br>- 10mWT<br>- BDI<br>- SF-36
Carling 2017	58	Both	MS; EDSS 6.1	IG: 21.6<br>CG: 20.2	MS: wait-list (N=25)	CoDuSe balance exercises (N=26)	60/min day, 2x/wk for 7 weeks	14 weeks	- TUG<br>- BBS<br>- 2MWT<br>- 10mWT<br>- FSMC<br>- FES
Carter 2014	45.9	Both	MS; EDSS 1.0 – 6.5	IG: 8.4<br>CG: 9.2	MS: Usual care (N=60)	Pragmatic aerobic exercise therapy (N=60)	60min/day, 3x/wk, for 12 weeks	9 months	- GLTEQ<br>- Daily steps<br>- MSQOL-54<br>- MSFC<br>- 6MWT
Castro-Sánchez 2012	48.0	Both	MS; EDSS 5.9 – 6.3	IG: 10.7<br>CG: 11.9	MS: relaxation exercises (N=37)	Ai Chi aquatic exercise program (N=36)	60min/day, 2x/wk, for 20 weeks	30 weeks	- MPQ<br>- RMDQ<br>- Spasm VAS<br>- MSIS-29<br>- MFIS<br>- FSS<br>- BDI<br>- Barthel index
Catena 2014	-	-	MS	-	MS: aerobic, relaxation + respiratory exercises (N=10)	Pilates (N=10)	60min/day, 2x/week	8 weeks	- FSS<br>- FAMS<br>- Pain
Cattaneo 2007	46.0	Both	RR-MS, PP-MS, SP-MS	13.8 over whole group	MS: conventional therapy (N=15)	- Balance rehabilitation improving motor & sensory strategies (N=23)<br>- Balance rehabilitation improving only motor strategies (N=12)	45min per session, 10-12 sessions over 3 wk	3 weeks	- BBS<br>- DGI<br>- DHI<br>- ABC
Claerbout 2012	43.5	Both	MS; EDSS 5.2	IG (full WBV): 12.1<br>IG (light WBV): 12.5<br>CG: 10.3	MS: usual care (N=17)	- Full WBV exercises (N=20)<br>- Light WBV exercises (N=18)	7-13min per session; 10 sessions over 3 weeks	3 weeks	- TUG<br>- BBS<br>- 3MWT<br>- Muscle strength lower limb
Coghe 2018	45.5	Both	RR-MS; EDSS 3.3	-	MS: TAU (N=11)	Aerobic + strength training (N=11)	60min/day, 3x/wk for 24 weeks	48 weeks	- Cognition<br>- MFIS<br>- TUG

Conklyn 2010	48.6	Both	MS	IG: 16.6<br>CG: 12.2	MS: usual care (N=5)	Home-based walking program (N=5)	20min/day for 2 weeks	6 weeks	- BBS<br>- Gait analysis<br>- Cardiopulmonary test<br>- Gait parameters<br>- T25FW<br>- MAS<br>- Muscle strength<br>- Pain<br>- Ambulation disability<br>- MS related disability<br>- SGI<br>- CGI
Costello 2009	-	Both	MS	-	MS: usual care (N=-)	Home walking program (N=-)	30min/day, 3x/week	12 weeks	- FSS<br>- 6MWT
Dalgas 2009	48.4	Both	RR-MS; EDSS 3.8	IG: 6.6<br>CG: 8.1	MS: usual care (N=19)	Lower body progressive resistance training (N=19)	2x/week, for 12 weeks	24 weeks	- Muscle strength<br>- 10mWT<br>- 6MWT<br>- CST<br>- SCT
Dalgas 2010a	48.4	Both	RR-MS; EDSS 3.8	IG: 6.6<br>CG: 8.1	MS: usual care (N=19)	Lower body progressive resistance training (N=19)	2x/week, for 12 weeks	24 weeks	- Muscle fiber size<br>- Thigh volume<br>- Muscle strength
Dalgas 2010b	48.4	Both	RR-MS; EDSS 3.8	IG: 6.6<br>CG: 8.1	MS: usual care (N=19)	Lower body progressive resistance training (N=19)	2x/week, for 12 weeks	24 weeks	- FSS<br>- MFI<br>- SF-36<br>- Depression<br>- Leg muscle strength
Dalgas 2013	48.7	Both	RR-MS; EDSS 3.8	IG: 6.6<br>CG: 8.1	MS: usual care (N=19)	Lower body progressive resistance training (N=19)	2x/week, for 12 weeks	24 weeks	- Muscle strength<br>- EMG
Deckx 2016[14]	48.5	Both	MS; EDSS 3.0	-	MS: usual care (N=25)	Endurance & resistance training (N=38)	5 sessions per 2 weeks	12 weeks	- Inflammatory marker levels
De Bolt 2004	50.7	Both	MS; EDSS 1-6.5	IG: 15.1<br>CG: 13.1	MS: usual care (N=18)	Resistance training (N=19)	35-50min/day, 3x/week	8 weeks	- Balance<br>- Leg power<br>- TUG
De Oliveira 2016	-	Both	MS	-	MS: wait-list (N=6)	Yoga (N=6)	60min/week	24 weeks	- BBS<br>- EDSS<br>- ADL
Dettmers 2009	42.8	Both	MS; EDSS 2.7	IG: 8.0<br>CG: 6.1	MS: stretching, balance and coordination training (N=15)	Endurance exercise (N=15)	45min/day, 3x/week, for 3 weeks	4 months	- MFIS<br>- FSMC<br>- BDI<br>- HAQUAMS

Dodd 2011	49.1	Both	RR-MS	-	MS: usual care + social program (N=37)	Lower body progressive resistance training (N=39)	45min/day, 2x/week, for 10 weeks	22 weeks	- Walking distance<br>- 2MWT<br>- Muscle strength<br>- MFIS<br>- WHOQoL-BREF<br>- MSSS-88
Doulatabad 2013	31.6	Female	MS	-	MS: usual care (N=30)	Yoga therapy (N=30)	60-90min/day, 8 sessions per month, for 3 month	4 month	- Pain<br>- QoL
Doulatabad 2014	31.6	Female	MS	-	MS: usual care (N=30)	Yoga therapy (N=30)	60-90min/day, 8 sessions per month	3 months	- Physical activity<br>- Sexual satisfaction
Duff 2018	45.4	Both	MS	-	MS: massage therapy (N=15)	Pilates + massage therapy (N=15)	50min/day, 2x/wk	12 weeks	- 6MWT<br>- TUG<br>- Balance<br>- MSQOL-54
Ebrahimi 2015	38.8	Both	RR-MS; EDSS 3.1	IG: 6.5<br>CG: 10.5	MS: usual care (N=17)	Low-intensity exercise + whole-body vibration (N=17)	3x/wk	10 weeks	- MFIS<br>- MSQOL-54<br>- BBS<br>- TUG<br>- 10mWT<br>- 6MWT<br>- FR<br>- Serum ghrelin<br>- Serum leptin<br>- Serum testosterone
Eftekhari 2012	34.4	Female	RR-MS EDSS 2-4	-	MS: usual care (N=12)	Resistance training (N=12)	3x/week	8 weeks	- Strength knee extensors<br>- 10mWT
Eftekharsadat 2015	35.2	Both	RR-MS, SP-MS	IG: 5.8<br>CG: 8.3	MS: usual care (N=15)	Virtual reality based balance training (N=15)	20min/day, 2x/week	12 weeks	- Muscle test<br>- TUG<br>- MAS<br>- BBS<br>- Fall risk test
Espinosa 2015	-	-	RR-MS; EDSS ≤2.5	-	MS: stretching exercises (N=9)	Qigong exercises (N=9)	60min/day, 3x/week	8 weeks	- Fatigue<br>- Pain<br>- MSQOL-54
Feys 2015	57.3	Both	MS; EDSS 7.2	IG: 21.1<br>CG: 14.4	MS: usual care (N=9)	Robot-assisted upper limb training (N=9)	30min/day, 3x/week	8 weeks	- Muscle strength<br>- Range of motion<br>- Movement duration<br>- Movement quality
Feys 2016	-	Both	MS	-	MS: waitlist (N=21)	Running training (N=21)	3x/week	12 weeks	- 6MWT<br>- MSWS<br>- Cognition

									- FSMC<br>- MSIS-29<br>- Brain imaging
Fimland 2010	53.5	Both	MS; EDSS 4.1	IG: 8.0<br>CG: 8.0	MS: conventional rehabilitation (N=7)	Conventional rehabilitation + Maximal strength training (N=7)	5x/week	3 weeks	- EMG soleus msucle
Forsberg 2016	54.2	Both	MS	IG: 15.0<br>CG: 16.0	MS: waitlist (N=38)	CoDuSe balance intervention (N=38)	50-60min/day, 2x/wk	7 weeks	- BBS<br>- TUG<br>- ABC<br>- MSWS-12<br>- FSS<br>- FGA
Fox 2016	54.1	Both	MS; EDSS 4-6.5	Pilates: 13.2<br>Exercise: 13.9<br>CG: 12.1	- MS: relaxation sessions (N=32)	- Pilates (N=33)<br>- Standardized physiotherapy exercises (N=35)	30min/week, for 12 weeks	16 weeks	- 10mWT<br>- FR<br>- MSWS-12<br>- ABC<br>- Walking velocity
Frevel 2015	45.5	Both	RR-MS; PP-MS; SP-MS; EDSS 3.8	IG: 16.1<br>CG: 22.3	MS: internet-based balance, postural control & strength exercises (N=9)	Hippotherapy (N=9)	20-30min/day, 2x/week	12 weeks	- BBS<br>- DGI<br>- Muscle strength<br>- TUG<br>- 2MWT<br>- FSS<br>- MFIS<br>- HAQUAMS
Gandolfi 2014	50.5	Both	RR-MS, SP-MS; EDSS 4.2	IG: 13.5<br>CG: 14.9	MS: sensory integration balance training (N=12)	Robot-assisted gait training (N=14)	50min/day, 2x/week, for 6 weeks	10 weeks	- Gait parameters<br>- BBS<br>- ABC<br>- SOT<br>- FSS<br>- MSQOL-54
Gandolfi 2015	48.4	Both	RR-MS; EDSS 3.3	IG: 12.3<br>CG: 15.2	MS: sensory integrated balance training (N=39)	Conventional rehabilitation (N=41)	50min/day, 3x/week, for 5 weeks	9 weeks	- BBS<br>- ABC<br>- SOT<br>- MSQOL-54<br>- FSS<br>- Number of falls
Garrett 2012a	50.1	Both	MS	IG (physio-therapy): 9.8<br>IG (fitness): 10.5<br>IG (yoga): 11.6<br>CG: 10.6	MS: usual care (N=71)	- Physiotherapy (N=80)<br>- Fitness (N=86)<br>- Yoga (N=77)	60min/week, for 10 weeks	12 weeks	- MFIS<br>- 6MWT<br>- MSIS-29

Garrett 2012b[15]	50.1	Both	MS	IG (physio-therapy): 9.8<br>IG (fitness): 10.5<br>IG (yoga): 11.6<br>CG: 10.6	MS: usual care (N=71)	- Physiotherapy (N=80)<br>- Fitness (N=86)<br>- Yoga (N=77)	30min/week, for 10 weeks	24 weeks	- MFIS<br>- 6MWT<br>- MSIS-29
Gervasoni 2014	47.7	Both	MS; EDSS 5.3	IG: 14.5<br>CG: 15.5	MS: conventional exercise (N=15)	Conventional exercise + treadmill training (N=15)	45min/day, 6x/week	2 weeks	- BBS<br>- FSS<br>- DGI<br>- PANAS<br>- Borg RPE scale
Ghafari 2008	31.5	Both	MS	-	MS: usual care (N=33)	Progressive muscle relaxation training (N=33)	60 sessions	8 weeks	- QoL
Giovannelli 2007	47.3	Both	Progressive MS with spasticity; EDSS 5.9	-	MS: only botulinum injection (N=20)	Physiotherapy after botulimum injection (N=20)	40min/day for 15 days	12 weeks	- MAS
Golzari 2010	33.0	Female	RR-MS	-	MS: usual care (N=10)	Combined endurance & resistance exercises (N=10)	75min/day, 3x/week	8 weeks	- IL-4<br>- IL-17<br>- IFN-Y<br>- Balance<br>- Muscle strength
Hasanpour 2016	-	Both	MS	-	MS: usual care (N=30)	- Yoga (N=30)<br>- Aerobic (N=30)	40min/day, 3x/week	12 weeks	- Pain<br>- Fatigue<br>- Psychosocial status
Hayes 2011	49.0	Both	MS; EDSS 5.2	IG: 12.5<br>CG: 11.9	MS: standard exercises (N=10)	High-intensity resistance training (N=10)	45-60min/day, 3x/week	12 weeks	- Muscle strength<br>- TUG<br>- 10mWT<br>- 6MWT<br>- BBS<br>- FSS
Hebert 2012	46.5	Both	RR-MS; SP-MS	IG: 6.5<br>CG (exercise): 5.1<br>CG (usual care): 9.1	MS: usual care (N=13)	- Bicycle endurance & stretching exercises (N=13)<br>- Vestibular rehabilitation program (N=12)	40-55min/day, 2x/week, for 6 weeks	14 week	- MFIS<br>- SOT<br>- 6MWT<br>- DHI<br>- BDI
Heine 2017	45.8	Both	MS; EDSS 3.0	IG: 12.0<br>CG: 7.0	MS: nurse consultation (N=45)	Aerobic training (N=45)	30min/day, 3x/wk	16 weeks	- Safety<br>- MFIS<br>- FSS
Hoang 2015	52.4	Both	MS; EDSS 4.2	IG: 11.6<br>CG: 13.4	MS: usual care (N=22)	Home-based step training (N=28)	30min/day, 2x/wk	12 weeks	- CSRT<br>- SST<br>- Postural sway

									- TUG<br>- 10mWT<br>- Cognition<br>- MSFC<br>- 9HPT
Hogan 2014[16]	54.0	Both	MS	IG (group physiotherapy): 18.0<br>IG (individual physiotherapy): 13.0<br>IG (yoga): 15.0<br>CG: 10.0	MS: usual care (N=19)	- Group physiotherapy (N=66)<br>- Individual physiotherapy (N=45)<br>- Yoga (N=16)	60min/wk, for 10 weeks	12 weeks	- BBS<br>- MFIS<br>- MSIS<br>- 6MWT
Hojjatollah 2012	-	Male	MS; EDSS 2-3.5	-	MS: usual care (N=10)	Combined resistance, aerobic and balance exercises (N=10)	60min/day, 3x/wk	8 weeks	- Muscle strength
Jäckel 2015	36.6	Both	RR-MS; EDSS 0-3.5	-	MS: usual care (N=-)	Internet guided endurance + strength training (N=-)	10-60min/day, 2-3x/week	12 weeks	- FSMC<br>- QoL<br>- Depression<br>- Cognition<br>- 2MWT<br>- TUG
Jonsdottir 2014	48.3	Both	MS; EDSS 2.5-5	-	MS: resistance training (N=6)	Treadmill training (N=13)	30min/session, total 20 sessions		- 2MWT<br>- 10mWT<br>- Cognition<br>- Muscle synergies
Kalron 2016a	43.6	Both	RR-MS; EDSS 4.4	IG: 11.3<br>CG: 12.4	MS: standardized physiotherapy (N=25)	Pilates (N=25)	30min/wk	12 weeks	- TUG<br>- 2MWT<br>- 6MWT<br>- FR<br>- BBS<br>- FSST<br>- MFIS<br>- MSWS-12
Kargarfard 2012	32.6	Female	RR-MS; EDSS 3.0	IG: 4.9<br>CG: 4.6	MS: usual care (N=16)	Aquatic exercise (N=16)	60min/day, 3x/week	8 weeks	- MFIS<br>- MSQOL-54
Kargarfard 2013[17]	32.6	Female	RR-MS	-	MS: usual care (N=11)	Aquatic exercise (N=10)	45-60min/day, 3x/week	8 weeks	- BBS<br>- 6MWT
Kargarfard 2018[33]	36.4	Female	RR-MS; EDSS 3.6	IG: 6.4<br>CG: 6.1	MS: usual care (N=15)	Aquatic exercise (N=17)	45-60min/day, 3x/week	8 weeks	- BBS<br>- 6MWT<br>- Sit-to-stand test<br>- MFIS
Kerling 2015	44.0	Both	MS; EDSS 2.9	-	MS: endurance training (N=30)	Endurance + resistance training (N=30)	40min/day, 2x/week	3 months	- MFIS<br>- SF-36

Khan 2008	50.3	Both	MS; EDSS 2-8	IG: 10.7<br>CG: 9.7	MS: wait-list (N=52)	Rehabilitation (N=49)	30-90min/day, 3-5x/week	12 months	- MSIS<br>- FIM<br>- GHQ-28
Khurana 2012	-	-	RR-MS	-	MS: medication (N=6)	- Physical therapy (N=6)<br>- Virtual exercise (N=6)	60min/day, 3x/week	12 weeks	- 6MWT
Kjolhede 2015	43.2	Both	RR-MS; EDSS 3.0	5.0 over whole group	MS: wait-list (N=17)	Progressive resistance training (N=18)	2x/week, for 24 weeks	48 weeks	- T25FW<br>- 2MWT<br>- 5x Sit-to-stand test<br>- MSWS-12<br>- Muscle area on MRI<br>- Muscle contractions
Kooshiar 2015	-	Female	MS	-	MS: usual care (N=19)	Aquatic exercise (N=18)	45min/day, 3x/week	8 weeks	- FSS<br>- MFIS<br>- QoL
Kucuk 2016	48.5	Both	MS; EDSS 3,0	IG: 14.8<br>CG: 14.2	MS: traditional exercises (9)	Pilates (N=11)	45min/day, 2x/week	8 weeks	- MSFC – cognition (PASAT)<br>- QoL<br>- BBS<br>- TUG<br>- BDI<br>- TIS<br>- Physical function
Learmonth 2012	51.6	Both	MS; EDSS 6.0	IG: 13.4<br>CG: 12.6	MS: usual care (N=12)	Group exercises (endurance, resistance, balance) (N=20)	45-60min/day, 2x/wk	12 weeks	- T25FW<br>- MSFC<br>- BBS<br>- FSS<br>- TUG<br>- Muscle strength lower limb<br>- HADS<br>- QoL<br>- 6MWT<br>- ABC
Learmonth 2017	48.4	Both	MS; EDSS 1.5	IG: 14.8<br>CG: 13.0	MS: waitlist (N=28)	Home-based resistance and aerobic exercise (N=29)	30min/day, 2x/wk	16 weeks	- Adherence<br>- ABC<br>- MSWS<br>- FSS<br>- HADS<br>- MSIS
Leavitt 2014	-	-	MS	-	MS: stretching exercises (N=-)	Aerobic training (N=-)	30min/day, 3x/week	12 weeks	- fMRI<br>- sMRI<br>- Cognition

Leone 2014	51.2	Both	MS; EDSS 4-6.5	15.6 over whole group	MS: physio-kinesiotherapy (N=27)	Wii balance exercises (N=19)	-	-	- BBS<br>- Barthel index<br>- SF-36<br>- RMI<br>- T25FW<br>- MSWS-12<br>- 9-HPT<br>- FIM
Louie 2015	48.6	Both	MS	-	MS: usual care (N=11)	Exercise + education (N=12)	2x/week	6 weeks	- Pain<br>- Fatigue<br>- 10mWT<br>- FR<br>- FSS<br>- MSIS
Majdinasab 2016	30.5	Female	MS; EDSS ≤4.5	IG: 2.9<br>CG: 3.4	MS: usual care (N=20)	Aquatic exercise (N=20)	60min/day, 3x/week	8 weeks	- FR<br>- LR<br>- TUG
McCullagh 2008	37.0	Both	RR-MS; SP-MS	IG: 5.4<br>CG: 5.0	MS: usual care (N=13)	Aerobic exercises (N=17)	40-50/day, 3x/week, for 12 weeks	24 weeks	- FAMS<br>- MSIS-29<br>- MFIS<br>- Borg RPE scale
Medina-Perez 2014	47.9	Both	RR-MS; EDSS 4.3	IG: 11.3<br>CG: 12.2	MS: usual care (N=12)	Lower limb resistance training (N=30)	2x/week, for 12 weeks	24 weeks	- Muscle strength<br>- Muscle endurance<br>- Muscle contraction
Miller 2011	54.6	Both	MS; EDSS 7.1	IG: 13.0<br>CG: 18.7	MS: usual care (N=15)	Home-based physiotherapy (N=15)	60min/day, 2x/week, for 8 weeks	16 weeks	- MSIS-29<br>- QoL<br>- Pain<br>- HADS<br>- Muscle strength<br>- 10mWT<br>- Timed sit-to-stand
Moghadasi 2015	32.4	Female	MS; EDSS 1-5	-	MS: usual care (N=13)	Resistance training (N=14)	3x/week	8 weeks	- BDNF
Moradi 2015	34.1	Male	MS; EDSS 2.9	-	MS: usual care (N=-)	Resistance training (N=-)	3x/week	8 weeks	- Balance<br>- Muscle strength<br>- Mobility
Mori 2011	38.4	Both	RR-MS; EDSS 2-6	-	- MS: only real iTBS (N=10)	- Land & aquatic exercises + sham iTBS (N=10)<br>- Land & aquatic exercises + real iTBS (N=10)	120min/day, 5x/week	2 weeks	- MAS<br>- MSSS-88<br>- FSS<br>- MSQOL-54<br>- Barthel index

Negahban 2013	36.6	Both	RR-MS, SP-MS; EDSS 3.7	IG: 8.5<br>CG (massage): 12.4<br>CG (massage + exercise): 9.6<br>CG (usual care): 7.2	- MS: massage therapy (N=12)<br>- MS: usual care (N=12)	- Exercise therapy (N=12)<br>- Exercise + massage (N=12)	30min/day, 3x/week	5 weeks	- Pain<br>- FSS<br>- MAS<br>- BBS<br>- TUG<br>- 10mWT<br>- 2MWT<br>- MSQOL-54
Nilsagard 2013	49.7	Both	MS	IG: 12.5<br>CG: 12.2	MS: usual care (N=42)	Balance exercises with Nintento Wii (N=42)	30min/day, 2x/wk	8 weeks	- MSIS-29<br>- TUG<br>- DGI<br>- T25FW<br>- FSST<br>- MSWS-12<br>- ABC<br>- TCS
O'Donnell 2011	-	-	MS	-	MS: usual care (N=-)	Physiotherapy (N=-)	60min/session, 10 sessions in 2 weeks, followed by 60min/ session, 10 sessions in 10 weeks	12 weeks	- MSIS-29
Oken 2004	49.0	Both	MS; EDSS 3.1		MS: wait-list (N=22)	- Yoga therapy (N=26)<br>- Aerobic exercise (N=21)	90min/week	6 months	- Cognition<br>- Alertness<br>- MFI<br>- Depression<br>- Stress<br>- QoL<br>- T25FW<br>- 9HPT<br>- Chair Sit and Reach
Ortiz-Rubio 2016	43.6	Both	MS; EDSS 5.9	-	MS: information on upper limb training (N=18)	Home-based upper limb training (N=18)	60min/day, 2x/wk	8 weeks	- Arm function<br>- Manual dexterity<br>- Handgrip strength
Ozgen 2016	41.0	Both	MS; EDSS 3.5	-	MS: waitlist (N=20)	Balance exercises + vestibular rehabilitation (N=20)	Rehabilitation: 30-45min/week<br>Home-exercises: 15-20min/day, 2x/week	8 weeks	- Dizziness<br>- ABC<br>- Balance<br>- TUG<br>- 6MWT<br>- DGI<br>- BDI<br>- MSQOL-54<br>- EDSS

Patti 2002	45.7	Both	PP-MS; SP-MS; EDSS 6.2	IG: 17.2<br>CG: 17.2	MS: Home exercises (N=53)	Rehabilitation program (N=58)	50-60min/day, 6x/wk, for 6 weeks	12 weeks	- SF-36<br>- FIS<br>- BDI
Patti 2003	45.7	Both	PP-MS; SP-MS	IG: 17.2<br>CG: 17.2	MS: Home exercises (N=53)	Rehabilitation program (N=58)	6x/wk, for 6 weeks	12 weeks	- FIM<br>- EDSS and functional system of Kurtzke scale
Pau 2018	46.0	Both	RR-MS; EDSS 3.5	-	MS: TAU (N=11)	Aerobic + strength training (N=11)	60min/day, 3x/wk	24 weeks	- Gait analysis
Paul 2014	51.7	Both	MS; EDSS 5.9	IG: 12.5<br>CG: 12.8	MS: usual care (N=15)	Web-based physiotherapy (N=15)	2x/week	12 weeks	- TUG<br>- BBS<br>- T25FW<br>- MSIS<br>- QoL<br>- HADS<br>- MS symptoms
Petajan 1996	40.1	Both	MS; EDSS ≤6.0	IG: 9.3<br>CG: 6.2	MS: usual care (N=25)	Aerobic exercise (N=21)	50min/day, 3x/week	15 weeks	- Muscle strength<br>- Blood lipids<br>- POMS<br>- FSS<br>- Sickness impact
Plow 2014	47.5	Female	RR-MS	IG: 8.0<br>CG: 10.0	MS: usual care (N=16)	Home-exercise program (N=14)	30-45min/day, 3-5/week	12 weeks	- SF-12 PCS<br>- MSIS<br>- TUG<br>- 6MWT
Prosperini 2013	36.2	Both	MS; EDSS 3.3	IG: 12.2<br>CG: 9.3	MS: usual care (N=18)	Home-based balance exercise with Wii (N=18)	30min/day, 5x/week, for 12 weeks	24 weeks	- MSIS-29<br>- T25FW<br>- FSST<br>- Standing balance
Rahnama 2011	33.4	Female	MS	-	MS: usual care (N=15)	Yoga (N=15)	60-75min/day, 3x/week	8 weeks	- Depression
Rampello 2007	41.0	Both	MS; EDSS 3.5	8.0 over whole group	MS: neuro-rehabilitation (N=11)	Aerobic training (N=8)	60min/day, 3x/week, for 8 weeks	24 weeks	- MFIS<br>- MSQOL-54<br>- 6MWT<br>- Pulmonary function
Razazian 2016	33.9	Female	PP-MS; SP-MS; RR-MS; EDSS ≤6.0	IG (yoga): 6.9<br>IG (aqua): 7.1<br>CG: 6.8	MS: usual care (N=18)	- Yoga (N=18)<br>- Aquatic exercise (N=18)	60min/day, 3x/week	8 weeks	- Fatigue<br>- BDI<br>- Paresthesia
Rietberg 2014	46.0	Both	MS; EDSS 3.5	IG: 7.0<br>CG: 8.0	MS: MS-nurse consultation (N=25)	Multidisciplinary rehabilitation (N=23)	Aerobic training part: 45min/day, 2x/week, for 12 weeks	24 weeks	- Fatigue<br>- MFIS<br>- MSIS-29<br>- FSS<br>- FIM

Robinson 2015	52.8	Both	MS	-	MS: usual care (N=18)	- Traditional balance exercises (N=18)<br>- Wii balance exercises (N=20)	40-60min/day, 2x/wk	4 weeks	- DIP<br>- IPA<br>- Postural sway<br>- Gait<br>- FSS<br>- MSWS-12<br>- WHODAS
Romberg 2004	43.9	Both	MS; EDSS 2.3	IG: 6.0<br>CG: 5.5	MS: usual care (N=50)	Strength & aerobic training (N=49)	Wk 1-3: 10 sessions, wk 4-26: 4-5x/wk	26 weeks	- T25FW<br>- 500mWT<br>- Static balance<br>- Upper limb endurance<br>- Knee flexion/extension
Romberg 2005[18]	43.9	Both	MS; EDSS 2.3	IG: 6.0<br>CG: 5.5	MS: usual care (N=48)	Long term exercise (mainly resistance) (N=47)	Wk 1-3: 10 sessions, wk 4-26: 4-5x/wk	6 months	- MSFC<br>- T25FW<br>- 9PHT<br>- Cognition<br>- Kurtzke functional system<br>- MSQOL-54<br>- FIM<br>- CES-D
Ruiz 2013	47.5	Both	MS; EDSS 4.8	IG: 7.4<br>CG: 13.8	MS: usual care (N=4)	Combined robot-assisted + body-weight supported treadmill training (N=4)	40min/day, 2x/wk, for 2 months	16 weeks	- 6MWT<br>- T25FW<br>- FR
Sabapathy 2011	55.0	Both	MS	10.0 over whole group	MS: resistance training (N=15)	Endurance training (N=6)	2x/wk	8 weeks	- FR<br>- FSST<br>- TUG<br>- 6MWT<br>- MSIS<br>- BDI<br>- SF-36<br>- MFIS
Salhofer-Polanyi 2013	53.4	Both	MS; EDSS 5.8	IG: 17.6<br>CG: 15.9	MS: wait-list (N=9)	Multidisciplinary rehabilitation (N=10)	Physiotherapy: 30min/session, 4-5x/day, 5x/week, for 3 weeks	15 weeks	- 2MWT<br>- 6MWT<br>- 50mWT<br>- 9HPT<br>- MSFC<br>- T25FW<br>- BBS<br>- EDSS<br>- Tinetti test<br>- FAMS

									- RMI<br>- MSSE<br>- Cognition
Sandroff 2016	42.9	Female	MS	IG: 11.4<br>CG: 12.2	MS: wait-list (N=5)	Treadmill walking (N=5)	15-40min/day, 3x/week	12 weeks	- Cognition<br>- 6MWT<br>- Disability
Sandroff 2017a[34]	43.5	Female	RR-MS	-	MS: wait-list (N=3)	Treadmill walking (N=5)	15-40min/day, 3x/week	12 weeks	- Cognition<br>- MRE
Sandroff 2017b	50.5	Both	MS	-	MS: stretching +toning activities (N=40)	Multimodal exercise (N=43)	30-60min/day, 3x/wk	24 weeks	- Cognition<br>- Gait<br>- Physical fitness<br>- T25FW<br>- 6MWT<br>- MSWS-12
Sangelaji 2014	32.6	Both	RR-MS	-	MS: usual care (N=30)	Combined aerobic and strengthening exercises (N=42)	90min/day, 3x/week, for 10 weeks	1 year	- EDSS<br>- Balance<br>- Fatigue<br>- 6MWT<br>- QoL
Solari 1999	44.8	Both	MS; EDSS 5.5	-	MS: home exercises (N=23)	Physical rehabilitation (N=27)	90min/day, for 3 weeks	15 weeks	- FIM<br>- Depression<br>- SF-36
Sosnoff 2014	60.0	Both	MS; EDSS 5.0	IG: 13.9<br>CG: 17.7	MS: wait-list (N=14)	Home-based exercises (N=13)	45-60min/day, 3x/week	12 weeks	- T25FW<br>- 6MWT<br>- TUG<br>- MSWS-12<br>- BBS<br>- ABC<br>- PPA
Sosnoff 2015	61.5	Both	MS; EDSS 5.9	IG: 15.0<br>CG (wait-list): 19.0<br>CG (education): 14.6<br>CG (exercise + education): 20.0	- MS: wait-list (N=9)<br>- MS: education program (N=9)	- Home-based exercise program (N=11)<br>- Combined exercise and education (N=8)	3x/week	12 weeks	- PPA<br>- Falls
Storr 2006	51.6	Both	MS; EDSS 6.5	IG: 9.0<br>CG: 9.0	MS: wait-list (N=65)	Multidisciplinary rehabilitation (N=41)	45min/day, 4-5x/week	5 weeks	- 9PHT<br>- MSIS<br>- 10mWT<br>- QoL<br>- VAS<br>- GNDS
Straudi 2014	52.6	Both	MS; EDSS 4.9	15.2 over whole group	MS: usual care (N=12)	Rehabilitation + home (gait, strengthening +	Rehabilitation: 120min/session, 10	14 weeks	- TUG<br>- 6MWT

						stretching) exercises (N=12)	sessions over 2 weeks<br>Home exercises: 60min/day, 3x/week		- 10mWT<br>- MSIS-29<br>- DGI<br>- FSS<br>- MSWS-12
Straudi 2015	55.3	Both	RR-MS; PP-MS; SP-MS; EDSS 5.8	IG: 18.6<br>CG: 17.1	MS: Conventional physiotherapy (N=28)	RAGT (N=30)	60min/day, 2x/week, for 6 weeks	18 weeks	- 6MWT<br>- 10mWT<br>- BBS<br>- TUG<br>- FSS<br>- PHQ-9<br>- SF-36
Surakka 2004	44.0	Both	MS; EDSS 2.6	IG: 6.0<br>CG: 5.5	MS: usual care (N=48)	Aerobic + resistance exercises (N=47)	30-35min/day, 4-5/week	26 weeks	- Fatigue<br>- FSS
Sutherland 2001	46.3	Both	MS	IG: 7.0<br>CG: 6.2	MS: usual care (N=11)	Land and aquatic aerobic exercise (N=11)	45min/day, 3x/week	10 weeks	- MSQOL-54<br>- Mood (depressed affect)
Tallner 2012	40.8	Both	MS; EDSS 2.8	-	MS: usual care (N=-)	Internet-based endurance + strength training (N=-)	3x/week	12 weeks	- Knee strength<br>- QoL<br>- Fatigue
Tarakci 2013	40.6	Both	MS; EDSS 4.3	IG: 9.0<br>CG: 8.4	MS: wait-list (N=55)	Group strengthening exercise (N=55)	60min/day, 3x/week	12 weeks	- BBS<br>- 10mWT<br>- FSS<br>- MAS<br>- SCT<br>- MSQOL-54
Van den Berg 2006	30 – 65	Both	MS	-	MS: usual care (N=9)	Treadmill training (N=10)	30min/day, 3x/week, for 4 weeks	12 weeks	- GNDS<br>- FSS<br>- 10mWT<br>- 2MWT
Van Kessel 2008	45.0	Both	MS; EDSS 3.5	IG: 6.7<br>CG: 5.5	MS: cognitive behavior therapy (N=35)	Muscle relaxation training (N=37)	50min/week, for 8 weeks	6 months	- HADS<br>- Fatigue<br>- Stress<br>- Sleep<br>- Work & social adjustment
Velikonja 2010	41.5	Both	RR-MS; PP-MS; SP-MS; EDSS 4.1	-	MS: yoga (N=10)	Sports climbing (N=10)	1x/wk	10 weeks	- EDSS<br>- MFIS<br>- Cognition<br>- MAS<br>- CES-D
Vermöhlen 2018	50.5	Both	MS; EDSS 5.4	IG: 16.5<br>CG: 17.6	MS: usual care (N=37)	Hippotherapy (N=30)	1x/wk	12 weeks	- BBS<br>- FSS<br>- Pain

Wens 2015a	45.7	Both	MS; EDSS 2.5	-	MS: usual care (N=11)	- High intensity continuous cardiovascular + resistance training (N=11)<br>- High intensity interval cardiovascular + resistance training (N=12)	5 sessions per 2 weeks	12 weeks	- Spasticity<br>- MSQOL-54<br>- Muscle fiber proportion<br>- Muscle strength<br>- Physical activity level
Wens 2015b	48.5	Both	MS; EDSS 3.3	-	MS: usual care (N=15)	Endurance & resistance training (N=30)	5 sessions per 2 weeks	24 weeks	- Blood glucose level<br>- Serum insulin level<br>- Knee muscle strength
Wens 2016a[19]	43.0	Both	RR-MS; EDSS 2.6	-	MS: usual care (N=7)	Endurance & resistance training (N=15)	45-75min/session, 5 session per 2 weeks	24 weeks	- Serum BDNF levels<br>- Muscle strength
Wens 2016b[20]	46.0	Both	MS; EDSS 2.5	-	MS: usual care (N=11)	- High-intensity interval training + resistance training (N=12)<br>- High-intensity continuous training + resistance training (N=11)	5 sessions per 2 weeks	24 weeks	- OGTT<br>- Muscle GLUT4 via biopsy
Wiles 2001	47.2	Both	MS	4.4 over whole group	MS: usual care (N= -)	- Home physiotherapy (N= -)<br>- Hospital physiotherapy (N= -)	45min, 2x/wk	8 weeks	- RMI<br>- Barthel index<br>- Balance<br>- 6MWT<br>- 9HPT<br>- HADS<br>- Cognition
Zenginler 2016	44.1	Both	MS; EDSS 4.1	-	MS: usual care (N=-)	Wii balance exercises (N=-)	45min/session, total 16 sessions		- BBS<br>- TUG
**Parkinson's disease**									
Allen 2010	67.0	Both	Idiopathic PD	IG: 7.0<br>CG: 9.0	PD: usual care (N=24)	Lower limb strengthening + balance exercises (N=24)	40-60min/day, 3x/week	6 month	- PD falls risk score<br>- BBS<br>- FOG<br>- Physical ability (SPPB)<br>- FES-I<br>- QoL (PDQ-39)
Ashburn 2007	72.2	Both	Idiopathic PD; HY-stage 2-4	IG: 7.7<br>CG: 9.0	PD: usual care (N=72)	Physiotherapy exercises (N=70)	60min/day, 1x/week by physiotherapist,	6 month	- Self reported falling<br>- FRT

							followed by 60 min daily self-practice, for 6 weeks		- BBS<br>- TUG<br>- Chair stand test<br>- SAS<br>- EQ-5D
Bega 2015	67.3	Both	PD; HY-stage 1-3	-	PD: resistance exercise (N=-)	Yoga (N=-)	-	12 weeks	- PDQ-39<br>- TUG<br>- MDS-UPDRS III
Belton 2014	68.1	Both	Idiopathic PD; HY-stage 1-4	IG: 6.5<br>CG: 8.8	PD: usual care (N=12)	Balance exercises (N=12)	60min/week	6 weeks	- TUG<br>- TUG-cognitive<br>- TUG-manual<br>- FES<br>- PDQ-39<br>- Brief BESTest<br>- MDS-UPDRS
Burini 2006	65.2	Both	PD; HY-stage 2-3	IG: 11.2<br>CG: 10.6	PD: Qigong (N=13)	Aerobic training (N=13)	45min/day, 3x/week, total 20 sessions in 7 weeks	15 weeks	- MDS-UPDRS III<br>- B'DS<br>- 6MWT<br>- PDQ-39<br>- BDI
Cakit 2007	71.8	Both	Idiopathic PD; HY-stage>3	5.6 over whole group	PD: usual care (N=27)	Speed-dependent treadmill training (N=27)	30min/week	8 weeks	- BBS<br>- Dynamic Gait Index<br>- FES<br>- Walking speed<br>- Walking distance
Canning 2012	61.8	Both	Idiopathic PD; HY-stage 1-2	IG: 6.1<br>CG: 5.2	PD: usual care (N=10)	Treadmill walking (N=10)	30-40min/day, 4x/week, for 6 weeks	12 weeks	- 6MWT<br>- PDQ-39<br>- MDS-UPDRS III<br>- Fatigue<br>- Walking speed
Canning 2014	70.7	Both	Idiopathic PD; HY-stage 2-3	IG: 7.5<br>CG: 8.3	PD: usual care (N=116)	Lower limb strengthening + balance exercises (N=115)	40-60min/day, 3x/week (1x/month by physical therapist, remaining self-practice)	6 month	- Number of falls<br>- Proportion of fallers<br>- PD falls risk score<br>- Knee extensor muscle strength<br>- Balance test<br>- Physical ability (SPPB)<br>- Walking velocity<br>- STS<br>- FOG<br>- FES-I<br>- Physical activity

									- SF-12v2<br>- SF-6D<br>- QoL (PDQ-39)<br>- PANAS
Capecci 2014	69.4	Both	Idiopathic PD; HY-stage 2-4	IG: 9.5<br>CG (PR+KT): 11.0<br>CG (waitlist): 9.6	PD: waiting list (N=7)	- Postural Rehabilitation (N=7)<br>- PR+ Kinesio taping (N=6)	40min/day, 3x/week, for 4 weeks	2 months	- BBS<br>- TUG<br>- Axial postural abnormalities
Carroll 2017	71.4	Both	Idiopathic PD; HY-stage 1-3	IG: 7.0<br>CG: 10.5	PD: usual care (N=10)	Aquatic therapy (N=11)	45min/day, 2x/weeks	6 weeks	- Gait<br>- Freezing<br>- MDS-UPDRS III<br>- PDQ-39
Carvalho 2015	63.7	Both	Idiopathic PD; HY-stage 1-3	-	PD: Strength training (N=8)<br>PD: physiotherapy (N=9)	- Aerobic training (N=5)	30-40min/day, 2x/week, for 12 weeks	12 weeks 2015	- MDS-UPDRS III<br>- MMSE<br>- CST<br>- ACT<br>- 2-MST<br>- CSRT<br>- BST<br>- 8-FT<br>- 10mWT<br>- BBS<br>- EEG mean frequency
Catalan 2013	-	-	PD; HY-stage 3	-	PD: motor imagery (N=7)	Treadmill training (N=7)	-	-	- MDS-UPDRS III<br>- FOG<br>- PDQ-39
Cheng 2016	66.5	Both	PD	IG (balance): 6.5<br>IG (turning): 6.1<br>CG: 8.1	PD: trunk exercises (N=12)	- Balance + strength training (N=12)<br>- Turning-based training (N=12)	30min/day, total 12 sessions	4-6 weeks	- FGA<br>- Balance
Choi 2013	63.2	Both	Idiopathic PD; HY-stage 1-2	IG: 5.2<br>CG: 5.2	PD: usual care (N=9)	Tai chi (N=11)	60min/day, 3x/week	12 weeks	- OLS<br>- 6MWT<br>- Gait<br>- TUG
Cholewa 2013	70.2	Both	Idiopathic PD; HY-stage 3	IG: 8.0<br>CG: 7.3	PD: usual care (N=30)	Rehabilitation exercises (N=40)	60min/day, 2x/week	12 weeks	- MDS-UPDRS I-III<br>- PDQ-39<br>- ADL
Clarke 2016	70.0	Both	Idiopathic PD	IG: 4.5<br>CG: 4.6	PD: usual care (N=381)	Physiotherapy + occupational therapy (N=381)	-	12 weeks	- PDQ-39<br>- EQ-5D<br>- SF-12<br>- ADL
Colgrove 2012	68.1	Both	PD; HU-stage 1-2	IG: 3.2	PD: usual care (N=5)	Yoga (N=8)	60min/day, 2x/week	12 weeks	- MDS-UPDRS

				CG: 3.7					- BBS<br>- Muscle strength<br>- Muscle flexibility
Combs 2013	67.3	Both	Idiopathic PD; HY-stage 2	IG: 3.5<br>CG: 4.2	PD: traditional group exercise (N=14)	Boxing training (N=17)	90min/session, 24-36 sessions	12 weeks	- BBS<br>- ABC<br>- TUG<br>- dTUG<br>- Gait velocity<br>- 6MWT<br>- PDQL
Comelia 1994	66.0	Both	PD; HY-stage 2-3	10.0 over whole group	PD: usual care (N=-)	Physical rehabilitation (N=-)	60min/day, 3x/week, for 4 weeks	6 months	- MDS-UPDRS<br>- GDS
Conradsson 2015	73.3	Both	Idiopathic PD; HY-stage 2-3	IG: 6.0<br>CG: 5.6	PD: usual care (N=49)	Highly challenging balance training (N=51)	60min/day, 3x/week	10 weeks	- Mini-BESTest<br>- Gait velocity<br>- FES-I<br>- MFE<br>- UPDRS-ADL<br>- Cognitive dual task
Corcos 2013	58.8	Both	Idiopathic PD; HY-stage 2-3	IG: 6.5<br>CG: 6.5	PD: mFC (N=24)	PRET (N=24)	2x/week	24 months	- Off-medication<br>- MDS-UPDRS-III<br>- Muscle strength<br>- Movement speed<br>- mPPT<br>- PDQ-39
Cugusi 2015	67.3	Both	Idiopathic PD; HY-stage 1-3	IG: 7.0<br>CG: 7.0	PD: usual care (N=10)	Nordic walking (N=10)	60min/day, 2x/week	12 weeks	- MDS-UPDRS III<br>- 6MWT<br>- FTSTS<br>- BBS<br>- TUG<br>- SRT<br>- PFS-16<br>- BDI<br>- Apathy<br>- NMSS
Dashtipour 2015	63.4	Both	Idiopathic PD; HY-stage 1-2	IG: 2.9<br>CG: 4.5	PD: LSVT BIG therapy (N=6)	General exercise (N=5)	60min/day, 4x/week, for 4 weeks	6 months	- MDS-UPDRS III + total<br>- BDI<br>- BAI<br>- MFIS
David 2015[21]	58.8	Both	Idiopathic PD; HY-stage 2	IG: 6.5<br>CG: 6.5	PD: mFC (N=24)	PRET (N=24)	2x/week	24 months	- Cognition

De Bruin 2015	65.6	Both	PD; HY-stage 2-3	IG: 6.4<br>CG: 4.5	PD: usual care (N=17)	Walking with music cueing (N=16)	30min/day, 3x/week	13 weeks	- MDS-UPDRS III<br>- Gait parameters
De Oliveira 2016	72.6	Both	Idiopathic PD; HY-stage 1-3	IG (individual): 4.4<br>IG (group): 4.3<br>CG: 4.0	PD: usual care (N=8)	- Group exercises (N=8)<br>- Individual exercises (N=8)	60min/day, 2x/week	24 weeks	- Cognition
Di Biagio 2014	-	-	PD	9.9 overall	PD: balance training (N=-)	- Dance therapy (N=-)<br>- Treadmill training (N=-)	Dance: 60min/day, 3x/week<br>Balance & treadmill: 30min/day, 5x/week	4 weeks	- 10MWT<br>- 6MWT<br>- TUG<br>- BBS<br>- FOG
DiFrancisco-Donoghue 2012	68.0	Both	PD; HY-stage 2	IG: 8.0<br>CG (vit): 9.0<br>CG (vit + ex): 7.0<br>CG (usual): 9.0	PD: usual care (N=9)<br>PD: vitamin supplementation (N=10)	- Aerobic + strength exercise (N=10)<br>- Exercise + vitamin supplementation (N=12)	40min/day, 2x/week	6 weeks	- Muscle strength<br>- Blood homocysteine, vit B6, B12, folate levels
Duncan 2012	69.2	Both	Idiopathic PD; HY-stage 2-3	IG: 5.8<br>CG: 7.0	PD: usual care (N=30)	Argentine Tango dance (N=32)	60min/day, 2x/week	12 months	- MDS-UPDRS I-III<br>- Mini-BESTest<br>- FOG<br>- 6MWT<br>- Gait velocity<br>- Upper extremity function (9HPT)
Duncan 2014	67.8	Both	Idiopathic PD; HY-stage 2-3	IG: 6.6<br>CG: 11.0	PD: usual care (N=5)	Argentine Tango dance (N=5)	60min/day, 2x/week	2 years	- MDS-UPDRS I-III<br>- Mini-BESTest<br>- Gait velocity<br>- TUG<br>- dTUG<br>- 6MWT<br>- FOG
Ebersbach 2008	73.8	Both	Idiopathic PD; HY-stage	IG: 7.5<br>CG: 7.0	PD: rehabilitation program + whole body vibration (N=14)	Rehabilitation program + balance training (N=13)	150min/day, 5x/week, for 3 weeks	7 weeks	- Tinetti score<br>- 10mWT<br>- MDS-UPDRS III<br>- Stand-walk-sit test<br>- Postural stability
Ebersbach 2010	67.3	Both	Idiopathic PD; HY-stage 1-3	IG: 6.1<br>CG (Nordic): 7.8<br>CG (domestic): 7.4	PD: Nordic walking (N-20)<br>PD: domestic exercises (N=20)	LSVT BIG therapy (N=20)	60min/day, 4x/week, for 4 weeks	16 weeks	- MDS-UPDRS III<br>- TUG<br>- PDQ-39<br>- 10mWT
Ellis 2005	64.0	Both	Idiopathic PD; HY-stage 2-3	-	PD: medication therapy (N=33)	Rehabilitation program (N=35)	90min/day, 2x/week	6 months	- MDS-UPDRS I-III<br>- CWS<br>- SIP-68

Fisher 2008	62.9	Both	Early-stage PD; HY-stage 1-2	Treadmill: 1.2<br>Physical therapy: 0.7<br>CG: 1.5	PD: education classes (N=10)	- Body-weight supported treadmill training (N=10)<br>- Traditional physical therapy (N=10)	Up to 45min/day, 24 sessions	8 weeks	- MDS-UPDRS<br>- Functional assessment walking, and sit-to-stand<br>- Walking test<br>- STS<br>- TMS
Fitton 2015	71.0	Both	Parkinson		PD: usual care (N=15)	Dance (N=36)	60min/day, 2x/week, for 10 weeks	6 months	- Balance<br>- Confidence<br>- Spinal posture<br>- Mobility
Foster 2013	69.2	Both	Idiopathic PD; HY-stage 2-4	IG: 5.8<br>CG: 7.0	PD: usual care (N=26)	Argentine Tango dance (N=26)	60min/day, 2x/week	12 weeks	- MDS-UPDRS I-III<br>- BDI<br>- ACS
Frazzitta 2012[22]	71.0	Both	Idiopathic PD; HY-stage 3	IG: 8.0<br>CG: 9.0	PD: usual care (N=25)	Intensive rehabilitation treatment (N=25)	180min/day, 5x/week, for 4 weeks → repeated after 12 months	12 months	- MDS-UPDRS II-III & total
Frazzitta 2014[22]	66	-	Idiopathic PD; HY-stage 1-1.5	IG: 8.0<br>CG: 8.0	PD: usual care (N=10)	Intensive rehabilitation treatment (N=15)	180min/day, 3x/week, for 4 weeks	4 weeks	- Serum BDNF<br>- MDS-UPDRS III
Frazzitta 2015[22]	68.5	Both	Idiopathic PD; HY-stage 1-1.5	-	PD: usual care (N=20)	Intensive rehabilitation treatment (N=20)	180min/day, 5x/week, for 4 weeks → repeated after 12 months	24 months	- MDS-UPDRS II-III<br>- 6MWT<br>- TUG<br>- PDDS
Ganesan 2014	58.1	Both	PD; HY-stage 2-3	PWSTT: 5.7<br>Gait: 4.9<br>CG: 5.5	PD: usual care (N=20)	- PWSTT (N=20)<br>- Conventional gait training (N=20)	30min/day, 4x/week	4 weeks	- Dynamic posturography<br>- BBS<br>- POMA
Ganesan 2015	58.2	Both	PD; HY-stage 2-3	PWSTT: 5.7<br>Gait: 4.9<br>CG: 5.5	PD: usual care (N=20)	- PWSTT (N=20)<br>- Conventional gait training (N=20)	30min/day, 4x/week	4 weeks	- MDS-UPDRS<br>- 10mWT<br>- Gait parameters
Gao 2014	68.9	Both	Idiopathic PD; HY-stage 1-4	IG: 9.2<br>CG: 8.4	PD: usual care (N=40)	Tai Chi (N=40)	60min/day, 3x/week, for 12 weeks	6 months	- MDS-UPDRS III<br>- BBS<br>- TUG<br>- Fall incidence
Gobbi 2013	67.8	Both	Idiopathic PD; HY-stage 1-3	IG (multimodal): 4.3<br>IG (posture & gait): 6.2<br>CG: 6.8	PD: cognitive activities (N=15)	- Multimodal exercises (N=15)<br>- Posture & gait exercises (N=15)	60min/day, 2x/week	4 months	- Cognition<br>- LSSI<br>- MDS-UPDRS
Goodwin 2011	71.1	Both	Idiopathic PD; HY-stage 1-4	IG: 9.1<br>CG: 8.2	PD: usual care (N=66)	Strength & balance training (N=64)	60min/day, 3x/week, for 10 weeks	20 weeks	- Number of falls<br>- FES-I<br>- EQ-5D

Gu 2013	68.5	Both	PD; HY-stage 1-3	IG: 5.8<br>CG: 6.2	PD: usual care (17)	PD weight bearing exercise for balance (N=18)	40-60min/day, 3x/week	8 weeks	- BBS<br>- TUG<br>- Phone-FITT<br>- FES<br>- Mini-BESTest<br>- MDS-UPDRS
Hackney 2008	67.0	Both	Idiopathic PD; HY-stage 1.5-3	IG: 8.7<br>CG: 12.0	PD: usual care (N=16)	Tai Chi (N=17)	60min/day, 2x/week, total 20 sessions	13 weeks	- MDS-UPDRS III<br>- BBS<br>- TUG<br>- 6MWT<br>- OLS<br>- Gait parameters
Hackney 2009	67.2	Both	Idiopathic PD; HY-stage 1-3	IG (tango): 6.9<br>IG (waltz): 9.2<br>CG: 5.9	PD: usual care (N=20)	- Tango dance (N=18)<br>- Waltz/Foxtrot dance (N=19)	60min/day, 2x/week, total 20 sessions	13 weeks	- MDS-UPDRS III<br>- BBS<br>- TUG<br>- 6MWT<br>- FOG<br>- Gait parameters
Harro 2014a	66.1	Both	Idiopathic PD; HY-stage 1-3	IG: 4.3<br>CG: 4.0	PD: rhythmic auditory cueing (N=11)	Speed dependent treadmill training (N=11)	30min/day, 3x/week, for 6 weeks	18 weeks	- RST<br>- BBS<br>- SMART EquiTest system for balance<br>- Falls incidence<br>- ABC<br>- PDQ-39
Harro 2014b[23]	66.1	Both	Idiopathic PD; HY-stage 1-3	IG: 4.3<br>CG: 4.0	PD: rhythmic auditory cueing (N=11)	Speed dependent treadmill training (N=11)	30min/day, 3x/week, for 6 weeks	18 weeks	- 10mWT<br>- 6MWT<br>- FGA
Hass 2012	65.5	Both	Idiopathic PD; HY-stage 1-3	IG: 11.1<br>CG: 6.4	PD: usual care (N=9)	Progressive resistance training (N=9)	2x/week	10 weeks	- Gait initiation
Hubble 2018	65.4	Both	Idiopathic PD	-	PD: fall prevention education (N=11)	Endurance exercises + fall prevention education (N=11)	90min/week	12 weeks	- Gait<br>- EMG
Kanegusuku 2017	65.0	Both	PD; HY-stage 2-3	IG: 8.5<br>CG: 9.0	PD: usual care (N=15)	Resistance training (N=15)	2x/week	12 weeks	- Cardiac autonomic modulation<br>- Muscle strength
Keus 2007	-	Both	Idiopathic PD;	-	PD: usual care (N=13)	Physiotherapy (N=14)	1-2x/week	10 weeks	- PDQ-39<br>- PAS<br>- PPOS
Kurtais 2008	64.8	Both	Idiopathic PD; HY-stage 2-3	IG: 5.3<br>CG: 5.4	PD: usual care (N=14)	Treadmill gait training (N=13)	40min/day, 3x/week, for 6 weeks	7 weeks	- 20m walking time<br>- Timed U-turn task<br>- Climbing stairs

									- Standing up from chair
Laupheimer 2011	69.4	-	PD; HY-stage 2-4	IG: 7.2<br>CG: 11.0	PD: usual care (N=23)	Cycling (N=21)	40min/day, 5x/week	10 weeks	- PDQ-8<br>- Motor function
Lee 2015	69.3	Both	PD	-	PD: neuro-development + electrical stimulation (N=10)	Virtual reality dance + neuro-development + electrical stimulation (N=10)	30min/day, 5x/week	6 weeks	- BBS<br>- ADL<br>- BDI
Lee 2018	65.7	Both	PD; HY-stage 1-3	IG: 4.5<br>CG: 4.4	PD: waitlist	Qigong dance training (N=	60min/day, 2x/week	8 weeks	- BBS<br>- BDI<br>- QoL<br>- MDS-UPDRS
Li 2012	68.7	Both	PD: HY-stage 1-4	Tai chi: 8.0<br>Resistance: 8.0<br>CG: 6.0	PD: stretching (N=65)	- Tai chi (N=65)<br>- Resistance training (N=65)	60min/day, 2x/week, for 24 weeks	9 months	- Maximum excursion<br>- Directional control<br>- MDS-UPDRS III<br>- TUG<br>- Falls incidence<br>- FRT<br>- Gait parameters<br>- Strength knee
Li 2014	68.7	Both	PD: HY-stage 1-4	Tai chi: 8.0<br>Resistance: 8.0<br>CG: 6.0	PD: stretching (N=65)	- Tai chi (N=65)<br>- Resistance training (N=65)	60min/day, 2x/week, for 24 weeks	9 months	- PDQ-8<br>- VPS<br>- 50-food speed walk test
Liao 2015a[24]	65.7	Both	Idiopathic PD; HY-stage 1-3	IG (traditional exercise): 6.9<br>IG (Wii program): 7.9<br>CG: 6.4	PD: no exercise (N=12)	- Traditional exercise + treadmill training (n=12)<br>- Virtual reality Wii fit exercise (N=12)	60min/day, 2x/week, for 6 weeks	16 weeks	- Obstacle crossing parameters<br>- LOS<br>- PDQ-39<br>- FES-I<br>- TUG<br>- SOT
Liao 2015b[24]	65.7	Both	Idiopathic PD; HY-stage 1-3	IG (traditional exercise): 6.9<br>IG (Wii program): 7.9<br>CG: 6.4	PD: fall prevention education (N=12)	- Traditional exercise + treadmill training (N=12)<br>- Virtual reality Wii fit exercise (N=12)	60min/day, 2x/week	6 weeks	- Walking ability<br>- Muscle strength<br>- Sensory integration ability
Liu 2016	64.2	Both	PD	-	PD: medication (N=26)	Qigong (N=	60min/day, 5x/week	10 weeks	- TUG<br>- Physical stability<br>- Muscle hardness
Mak 2008	64.0	-	PD; HY-stage 2-3	IG: 6.1<br>CG (cued-training): 5.9<br>CG (usual care): 5.9	PD: usual care (N=18)<br>PD: cued-training (N=21)	Mobility and strengthening exercises (N=21)	45min/day, 2x/week, for 4 weeks	6 weeks	- Motion analysis
McCamish 2013	63.9	Male	PD; HY-stage 1-3	-	PD: stretching exercises (N=4)	Cardiovascular exercise (N=3)	40min/day, 3x/week	12 weeks	- Cognition<br>- PDQ-39

Mezzarobba 2009	75.0	-	PD; Hy-stage 1-3	9 over whole group	PD: neurocognitive rehabilitation with motor imagery (N=11)	Treadmill training (N=10)	20 sessions	12 weeks	- PDQ-39<br>- TUG<br>- 6MWT<br>- Cognition<br>- FOG<br>- BBS<br>- Disability
Mirabella 2016	-	-	Idiopathic PD	-	PD: emotional training via theater program (N=12)	Physiotherapy (N=12)	-	18 months	- Cognition<br>- Motor symptoms<br>- Well being
Miyai 2002	69.7	Both	PD; HY-stage 2.5-3	IG: 4.1<br>CG: 4.5	PD: physical therapy (N=12)	Body-weight supported treadmill training (N=12)	45min/day, 3x/week, for 1 month	6 months	- MDS-UPDRS I-IV<br>- Gait parameters
Mollinedo-Cardalda 2017	64.4	Both	PD; HY-stage 2.0	IG: 5.8<br>CG: 5.7	PD: aerobic exercises (N=10)	Pilates (N=12)	60min/day, 2x/week	12 weeks	- TUG<br>- MDS-UPDRS<br>- Feasibility<br>- Muscle strength
Monticone 2015	73.8	Both	Idiopathic PD; HY-stage 2.5-4	IG: 15.3<br>CG: 15.7	PD: multidisciplinary rehabilitation (N=35)	Physiotherapy (N=35)	90min/day, for 8 weeks	12 months	- MDS-UPDRS III<br>- BBS<br>- FIM<br>- PDQ-39
Morris 2009	67.0	-	Idiopathic PD; HY-stage 2-3	-	PD: movement strategies training (N=14)	Musculoskeletal exercises (N=14)	45min per session, max 16 session in 2 weeks	14 weeks	- MDS-UPDRS II-III<br>- 10mWT<br>- TUG<br>- 2MWT<br>- Balance pull test<br>- PDQ-39
Morris 2015	67.9	Both	Idiopathic PD; HY-stage 1-4	IG: 7.2<br>CG (movement training): 6.0<br>CG (life skills): 6.9	PD: movement strategy training (N=69)<br>PD: life skills discussion (N=71)	Progressive strength training (N=70)	120min/day, 1x/week, for 8 weeks	14 months	- Falls rate<br>- MDS-UPDRS II-III<br>- 6MWT<br>- TUG<br>- PDQ-39<br>- ED-5D VAS
Morrone 2016	72.5	Both	Idiopathic PD; HY-stage 1-3	IG: 6.5<br>CG: 6.3	PD: perceptive rehabilitation (N=10)	Conventional physical therapy (N=10)	45min/day, 3x/week	4 weeks	- Gait analysis<br>- Fall risk index
Nadeau 2014	62.8	Both	Idiopathic PD; HY-stage <2	-	PD: light exercises (N=14)	- Speed treadmill training (N=17)<br>- Speed + incline treadmill training (N=14)	60min/day, 3x/week	24 weeks	- Gait parameters<br>- MDS-UPDRS I-IV<br>- Cognition<br>- BDI<br>- PDQ-39<br>- ABC

Ni 2015	72.6	Both	Idiopathic PD; HY-stage 1-3	IG (PWT): 6.6<br>IG (Yoga): 6.9<br>CG: 5.9	PD: health education classes (N=12)	- Power training (PWT; N=14)<br>- Yoga (N=15)	60min/day, 2x/week	12 weeks	- MDS-UPDRS III<br>- BBS<br>- Mini-BESTest<br>- TUG<br>- FRT<br>- SLS<br>- PS<br>- Muscle strength<br>- 10mWT
Ni 2016a[25]	73,1	Both	Idiopathic PD; HY-stage 1-3	IG: 6.9<br>CG: 5.9	PD: usual care (N=12)	Power yoga (N=15)	60min/day, 2x/week	12 weeks	- Bradykinesia<br>- Rigidity<br>- Muscle strength<br>- PDQ-39
Ni 2016b[25]	73.3	Both	Idiopathic PD; HY-stage 1-3	IG: 6.6<br>CG: 5.9	PD: usual care (N=12)	Power resistance training (N=14)	2x/week	12 weeks	- Bradykinesia<br>- Muscle strength<br>- PDQ-39
Ortiz-Rubio 2018	-	-	PD	-	PD: TAU (N=-)	Resistance training (N=-)	-	8 weeks	- Balance
Park 2014a	59.9	Both	Idiopathic PD; HY-stage 1-2	IG (communal): 6.2<br>IG (individual): 5.9<br>CG: 6.3	PD: no exercise (N=15)	Fitness cycling (N=16)	60min/day, 3x/week, for 24 weeks	48 weeks	- MDS-UPDRS III + total<br>- Tinetti score<br>- PDQ-39<br>- BDI<br>- Timed walk
Park 2014b	72.5	Both	Idiopathic PD; HY-stage 1-3	-	PD: usual care (N=9)	- Communal strength + balance exercises (N=10)<br>- Individual strength + balance exercises (N=10)	60min/day, 3x/week	10 weeks	- Gait parameters<br>- Fear of falling
Paolucci 2017	66.5	Both	Idiopathic PD; HY-stage 1-3	-	PD: home-based exercise (N=10)	Mezieres rehabilitation (N=10)	60min/day, 5x/week, for 5 weeks	12 weeks	- BBS<br>- Pain<br>- 6MWT<br>- SF_36<br>- FGA<br>- DGI<br>- MDS-UPDRS
Picelli 2012b	68.3	Both	Idiopathic PD; HY-stage 3-4	7.5 over whole group	PD: robot-assisted gait training (N=17)	Physical therapy (N=17)	40min/day, 3x/week, for 4 weeks	2 months	- BBS<br>- ABC<br>- TUG<br>- 10mWT<br>- MDS-UPDRS III<br>- Nutt's rating scale
Picelli 2015	68.9	Both	Idiopathic PD; HY-stage 3	IG: 8.3<br>CG: 7.5	PD: robot-assisted gait training (N=33)	Balance training (N=33)	45min/day, 3x/week, for 4 weeks	8 weeks	- BBS<br>- ABC<br>- TUG

Picelli 2016	71.4	Both	Idiopathic PD; HY-stage 3	IG: 11.2<br>CG: 10.8	PD: lifestyle program (N=8)	Treadmill training (N=9)	45min/day, 3x/week	4 weeks	- MDS-UPDRS III<br>- Cognition<br>- 6MWT<br>- 10MWT<br>- BDI
Pikel 2015	64.5	Both	Idiopathic PD; HY-stage 1-4	-	PD: orientation about fall prevention (N=-)	Physical gait training with mental practice (N=-)	-	14 days	- MDS-UPDRS<br>- TUG<br>- Balance
Poliakoff 2013	65.2	Both	PD	IG: 7.4<br>CG: 4.7	PD: no exercise (N=16)	Gym training (N=16)	60min/day, 2x/week	10 weeks	- PDQ-39<br>- MDS-UPDRS III<br>- Timed chair<br>- 6 foot walk
Prodoehl 2015[21]	58.8	Both	Idiopathic PD; HY-stage 2-3	IG: 6.5<br>CG: 6.5	PD: mFC (N=24)	PRET (N=24)	60-90min/day, 2x/week	24 months	- mPPT<br>- STS<br>- FRT<br>- TUG<br>- BBS<br>- 6MWT<br>- Walking speed
Protas 2005	72.5	Men	Idiopathic PD; HY-stage 2-3	IG: 7.1<br>CG: 8.1	PD: usual care (N=9)	Gait and step treadmill training (N=9)	60min/day, 3x/week, for 8 weeks	12 weeks	- Gait parameters<br>- Freezing of gait<br>- Fall frequency<br>- Dynamic balance
Qutubuddin 2013	68.2	Both	PD	7.2 over whole group	PD: usual care (N=10)	Cycling (N=13)	30min/day, 2x/week	8 weeks	- BBS<br>- MDS-UPDRS III<br>- PDQ-39<br>- Fingertapping
Reuter 2011	62.4	Both	PD; HY-stage 2-3	IG (walk): 6.0<br>IG (Nordic walk): 5.3<br>CG: 5.2	PD: flexibility + relaxation exercises (N=30)	- Walking (N=30)<br>- Nordic walking (N=30)	70min/day, 3x/week	24 weeks	- Walking speed<br>- MDS-UPDRS<br>- PDQ-39<br>- Pain
Reuter 2012	64.0	Both	PD; HY-stage 2-4	IG (transfer): 7.9<br>IG (transfer + motor): 8.3<br>CG: 8.2	PD: cognitive training (N=71)<br>PD: cognitive + transfer training (N=75)	- Endurance training + cognitive training + transfer & psychomotor training (N=76)	- Cognitive training: 60min/day, 4x/week<br>- Transfer training: 90min/day, 3x/week<br>- Motor training: 60min/day, 3x/week<br><br>4 weeks at rehabilitation unit	7 months	- Cognition<br>- PDQ-39<br>- HDRS

							followed by 6 months at home		
Ridgel 2013	-	-	PD	-	PD: usual care (N=-)	Cycling (N=-)	12 sessions	4 weeks	- MDS-UPDRS III<br>- Balance
Romenets 2015	63.8	Both	Idiopathic PD; HY-stage 1-3	IG: 5.5<br>CG: 7.7	PD: wait-list (N=15)	Argentine Tango (N=18)	60min/day, 2x/week	12 weeks	- MDS-UPDRS III<br>- Mini-BESTest<br>- TUG<br>- dTUG<br>- FOG<br>- Cognition<br>- BDI<br>- Apathy<br>- PDQ-39<br>- CGI-C
Sacheli 2012	71.1	Both	PD	-	PD: SAFE (N=9)	- Aerobic exercise + SAFE (N=10)<br>- Resistance exercise + SAFE (N=14)	60min/day, 3x/week	24 weeks	- MDS-UPDRS III<br>- TUG
Sage 2009	65.9	Both	Idiopathic PD	IG: 3.2<br>CG (sensory attention): 4.7<br>CG (wait-list): 2.5	PD: wait-list (N=15)<br>PD: SAFE (N=21)	Lower limb aerobic training (N=17)	30min/day, 3x/week	12 weeks	- MDS-UPDRS III<br>- Posture & gait measure<br>- TUG<br>- Gait parameters
Santos 2017a	67.8	Both	Idiopathic PD; HY-stage 1.5-3	IG: 5.6<br>CG: 5.4	PD: balance training (N=21)	Resistance training (N=19)	60min/day, 2x/week	12 weeks	- Postural balance<br>- BESTest
Santos 2017b	73.6	Both	Akinetic PD; HY-stage 1-2	IG: 10.8<br>CG: 10.5	PD: usual care (N=15)	Resistance training (N=13)	60-70min/day, 2x/week, for 8 weeks	12 weeks	- MDS-UPDRS<br>- Gait<br>- FOG<br>- PDQ-39
Schenkman 1998	70.9	Both	PD; HY-stage 2-3	-	PD: usual care (N=24)	Exercises to improve spinal flexibility + physical performance (N=27)	45-60min/day, 3x/week	10 weeks	- Range of motion<br>- Spinal flexibility<br>- 6MWT
Schenkman 2012	64.7	Both	Early or mild PD; HY-stage 1-3	IG (Flexibility): 4.9<br>IG (aerobic): 3.9<br>CG: 4.5	PD: home exercise (N=41)	- Flexibility/ balance/function exercise (N=39)<br>- Aerobic exercise (N=41)	3x/week, for 4 months, tapered to 1x/month	16 months	- Physical function<br>- FRT<br>- MDS-UPDRS II-III<br>- PDQ-39
Schilling 2010	59.2	Both	PD; HY-stage 1-2.5	-	PD: usual care (N=9)	Lower body resistance training (N=9)	2x/week	8 weeks	- Lower body strength<br>- 6MWT<br>- TUG<br>- ABC

Schlenstedt 2015	75.7	Both	Idiopathic PD; HY-stage 2.5 – 3	-	PD: balance training (N=20)	Resistance training (N=20)	60min/day, 2x/week, for 7 weeks	12 weeks	- FAB<br>- TUG<br>- CGI<br>- MDS-UPDRS III + total<br>- PDQ-39<br>- BDI<br>- PASE
Schmitz-Hübsch 2006	63.5	Both	PD	IG: 6.0<br>CG: 5.6	PD: usual care (N=24)	Qigong (N=32)	60min/week	8 weeks	- PDQ-39<br>- MADRS<br>- MDS-UPDRS III
Sedaghati 2016	58.4	Both	Idiopathic PD; HY-stage 2.6	IG (with pad): 4.9<br>IG (without pad): 5.2<br>CG: 4.9	PD: usual care (N=15)	- Balance exercises with balance pad (N=15)<br>- Balance exercises without balance pad (N=15)	60min/day, 3x/week	10 weeks	- Number of falls<br>- FES-I<br>- BBS<br>- TUG
Sharma 2015[26]	68.1	Both	PD; HY-stage 1-2	IG: 3.8<br>CG: 3.8	PD: usual care (N=5)	Yoga (N=8)	60min/day, 2x/week	12 weeks	- GDS<br>- SF-36<br>- MDS-UPDRS III<br>- FES
Shen 2012	64.8	Both	PD; HY-stage 2-3	IG: 5.8<br>CG: 7.1	PD: repetitive step training (N=15)	Lower limb strength training (N=14)	± 60min/day, 3x/week	4 weeks	- Posture and gait<br>- Gait parameters<br>- Fall incidence<br>- LOS
Shen 2014[27]	64.3	Both	Idiopathic PD; HY-stage 2-3	IG: 8.1<br>CG: 6.6	PD: lower-limb strength training (N=25)	Balance and gait training (N=26)	60min/day, 3x/week, for 8 weeks at laboratory, and 20min/day, 5x/week, for 4 weeks at home	15 months	- ABC<br>- SLS<br>- LOS<br>- Gait parameters
Shen 2015[27]	64.3	Both	Idiopathic PD; HY-stage 2-3	IG: 8.1<br>CG: 6.6	PD: lower-limb strength training (N=25)	Balance and gait training (N=26)	60min/day, 3x/week, for 8 weeks at laboratory, and 20min/day, 5x/week, for 4 weeks at home	15 months	- Fall rate<br>- Number of fallers<br>- SLS<br>- MCT<br>- Walking test
Shulman 2013	65.8	Both	PD; HY-stage 1-3		PD: stretching & resistance exercises (N=28)	- High-intensity treadmill training (N=26)<br>- Low-intensity treadmill training (N=26)	3x/week, for 3 months	4 months	- 6MWT<br>- 10mWT<br>- Muscle strength<br>- ADL<br>- TUG<br>- MDS-UPDRS III & total<br>- BDI

									- FES<br>- PDQ-39<br>- PFS
Silva-Batista 2016	64.2	Both	Idiopathic PD; HY-stage 2-3	IG (RT): 9.6<br>IG (RTI): 10.5<br>CG: 10.7	PD: Education + bingo games (N=13)	- Resistance training (RT; N=13)<br>- Resistance training with instability (RTI; N=13)	60min/day, 2x/week	12 weeks	- TUG<br>- Cognition<br>- PDQ-39<br>- MDS-UPDRS III<br>- Muscle strength
Silva-Batista 2017[35]	64.5	Both	Idiopathic PD; Hy-stage 2.5	IG: 10.0<br>CG: 11.6	PD: Education + bingo games (N=13)	Resistance training (N=	50min/day, 2x/week	12 weeks	- Sleep quality
Smania 2010	67.5	Both	Idiopathic PD; HY-stage 3-4	IG: 10.4<br>CG: 8.6	PD: general physical exercises (N=31)	Balance training (N=33)	50min/day, 3x/week, for 7 weeks	11 weeks	- BBS<br>- ABC<br>- Fall incidence<br>- MDS-UPDRS I-IV<br>- GDS<br>- Postural transfers
Sparrow 2016	66.7	Both	Idiopathic PD; HY-stage 2-3	4.3 over entire group	PD: usual care (N=11)	Balance exercises (N=10)	90min/day, 2x/week	12 weeks	- Mini-BESTest<br>- FES-I<br>- Falls
Stack 2012	74.0	Both	PD; HY-stage 1-4	IG: 8.0<br>CG: 7.0	PD: usual care (N=23)	Physiotherapy (N=24)	60min/day, 3x/week, for 4 weeks	12 weeks	- FRS<br>- HR-QoL<br>- MDS-UPDRS posture<br>- SAS<br>- Sit-to-stand time<br>- Chair transfer score
Stozek 2015	65.5	Both	PD; HY-stage 1.5-3	IG: 4.6<br>CG: 4.3	PD: usual care (N=31)	Rehabilitation (N=30)	First 2 week: 120min 2x/day, Last 2 week: 120min/day, 3x/week, total 28 sessions	4 weeks	- Balance<br>- Gait<br>- Spinal axial rotation
Taheri 2011	-	-	PD	-	PD: usual care (N=10)	Tension + supple exercises (N=12)	60min/day, 4x/week	10 weeks	- BBS<br>- Tinetti scale<br>- Gait
Tickle-Degnen 2010	66.3	Both	Idiopathic PD; HY-stage 2-3		PD: usual care (N=41)	- 18 hours rehabilitation (N=37)<br>- 27 hours rehabilitation (N=39)	3-4,5hours/week, for 6 weeks	6 months	- PDQ-39
Tramontano 2016	70.0	Both	PD	IG: 8.8<br>CG: 7.9	PD: blindfolded balance training (N=15)	Physical therapy (N=15)	90min/day, 5x/week	8 weeks	- MDS-UPDRS III<br>- Gait parameters

Vergara-Diaz 2018	63.9	Both	Idiopathic PD; HY-stage 1-2.5	IG: 2.9<br>CG: 2.9	PD: usual care (N=16)	Tai Chi (N=16)	60min/day, 2xweek + 60min/week at home	6 months	- Safety<br>- Gait<br>- MDS-UPDRS<br>- PDQ-39<br>- TUG<br>- ABC<br>- Cognition
Wade 2003	70.9	Both	PD	-	PD: no rehabilitation (N=72)	Multidisciplinary rehabilitation (N=72)	2 hours/week, for 6 weeks	24 weeks	- PDQ-39<br>- SF-36<br>- EQ-5D<br>- NHPB<br>- HADS<br>- MDS-UPDRS
Wong-Yu 2015a	61.1	Both	PD; HY-stage 2-3	IG: 7.3<br>CG: 5.4	PD: upper limb training (N=38)	Balance training (N=32)	120min/day, 1x/week, for 8 weeks	8 months	- Mini-BESTest<br>- FRT<br>- FTSTS<br>- TUG<br>- dTUG<br>- OLS
Wong-Yu 2015b[28]	61.0	Both	PD; HY-stage 2-3	IG: 7.1<br>CG: 5.6	PD: upper limb training (N=43)	Balance training (N=41)	120min/day, 1x/week, for 8 weeks	14 months	- BESTest<br>- Gait speed<br>- dTUG<br>- ABC
Xiao 2015	67.3	Both	Idiopathic PD; HY-stage 1-3	IG: 5.5<br>CG: 6.2	PD: walking (N=48)	Baduanjin Qigong + walking (N=48)	Qigong: 45min/day, 4x/week<br>Walking: 30min/day	6 months	- Gait<br>- TUG<br>- 6MWT<br>- BBS<br>- MDS- UPDRS III
Yang 2010	67.2	Both	Idiopathic PD; HY-stage 1-3	IG: 4.8<br>CG: 5.3	PD: conventional therapy (N=17)	Downhill walking (N=16)	30min/day, 3x/week, for 4 weeks	8 weeks	- Gait parameters<br>- Muscle strength
Yen 2011	70.7	Both	Idiopathic PD; HY-stage 2-3	IG: 6.1<br>CG (virtual reality): 6.0<br>CG (usual care): 7.8	PD: usual care (N=14)	- Balance training (N=14)<br>- Virtual reality balance training (N=14)	30min/day, 2x/week, for 6 weeks	10 week	- Equilibrium scores
Zhang 2015	65.2	Both	Idiopathic PD; HY-stage 1-4	IG: 6.8<br>CG: 4.9	PD: multimodal exercises (N=20)	Tai Chi (N=20)	60min/day, 2x/week	12 weeks	- BBS<br>- TUG<br>- Gait parameters<br>- MDS-UPRS III
**Schizophrenia**									
Battaglia 2013	35.5	Male	Schizophrenia and/or schizoaffective	-	TAU (N=11)	Soccer (N=12)	100-120 min/day, 2x/week	12 weeks	- SF-12 health survey (PCS-12, MCS-12)

			disorder						
Beebe 2005	52.0	Both	Schizophrenia	-	TAU (N=6)	Treadmill walking program (N=6)	30 min/day, 3x/week	16 week	- PANSS
Behere 2011	31.7	Both	Schizophrenia	Yoga: 10.5<br>Exercise: 7.2<br>CG: 10.1	Waitlist (N=26)	- Yoga (N=34)<br>- Exercise (N=31)	Not mentioned	- 1 month by yoga instructor<br>- 2 month home practice	- PANSS<br>- SOFS<br>- TRENDS
Bhatia 2017	35.2	Both	Schizophrenia	Yoga: 8.5<br>Exercise: 8.7<br>CG: 8.5	TAU (N=92)	- Yoga (N=104)<br>- Physical exercise (N=90)	60min/day, 6x/week, for 21 days	6 months	- Cognition
Duraiswamy 2007	31.9	Both	Schizophrenia	IG: 8.3<br>CG: 6.8	Physical training (N=30)	Yoga (N=31)	60min/day, 5x/week for 3 weeks by yoga instructor. Thereafter at home for 3 months	4 months	- PANSS<br>- SOFS<br>- WHOQOL-BREF
Falkai 2013	35.0	Male	Schizophrenia	-	Healthy controls: aerobic exercise (N=8)<br>SZ patients: table football (N=8)	Aerobic exercise (N=8)	30min/day, 3x/week	3 months	- MRI of the cortex (gray matter density, cortical surface expansion)<br>- PANSS
Gholipour 2012	40.7	Male	Schizophrenia	At least 3 year history of disease	- TAU (N=15)<br>- Token behavior therapy (N=15)	Exercise (N=15)	120min/day, 3x/week	3 months	- SANS
Ho 2016	54.0	Both	Schizophrenia	Tai chi: 28.5<br>Exercise: 30.6<br>CG: 30.7	Waitlist (N=49)	- Tai chi (N=51)<br>- Aerobic exercise (N=51)	1x60min/wk, 2x45min/wk, for 12 weeks	6 mnd	- PANSS<br>- Cognition<br>- Barthel's ADL<br>- Salivary cortisol
Hu 2004	-	-	Schizophrenia	-	TAU (N=58)	Music & sport therapy (N=58)	-	-	- BPRS
Ikai 2013	53.2	Both	Schizophrenia or related psychotic disorder	IG: 24.5<br>CG: 27.7	Regular day-care program (social skills, psycho-education; N=24)	Yoga (N=25)	60min/week	8 weeks	- PANSS<br>- EQ-5D

Kaltsatou 2014	60.0	Both	Schizophrenia	-	TAU (N=15)	Dancing (N=16)	60min/day, 3x/week	8 months	- GAF<br>- PANSS<br>- QOL enjoyment and satisfaction questionnaire
Kim 2014	49.7	-	Paranoid schizophrenia	>3	TAU (N=15)	Aerobic exercise + strength training (N=25)	60min/day, 3x/week	12 weeks	- BDNF
Kimhy 2015	36.9	Both	Schizophrenia or related disorders	-	TAU (N=17)	Aerobic exercise (N=16)	1h/day, 3x/week	12 weeks	- BDNF<br>- MATRICS cognitive battery
Lin 2015	24.6	Female	Schizophrenia Spectrum Disorder	Yoga: 2.5<br>Exercise: 2.4<br>CG: 2.0	Waitlist (N=46)	- Yoga (N=48)<br>- Aerobic exercise (N=46)	60min/day, 3x/wk	12 weeks	- PANSS<br>- SF-36<br>- Cognition<br>- CDSS<br>- Structural MRI
Lin 2017[36]	24.6	Female	Schizophrenia Spectrum Disorder	-	Waitlist (N=12)	- Yoga (N=23)<br>- Aerobic exercise (N=23)	60min/day, 3x/wk	12 weeks	- Brain functional fluctuation
Loh 2016	21.6	Both	Schizophrenia	-	TAU (N=52	Walking (N=52)	20-40min/day, 3x/wk	12 weeks	- SF-36<br>- PANSS<br>- PSP
Manjunath 2013	31.4	Both	Schizophrenia	IG: 10<br>CG: 8.1	Exercise therapy (N=44)	Yoga (N=44)	60min/day, 5x/week	- 2 weeks by yoga instructor<br>- 4 weeks yoga at home	- PANSS<br>- HDRS<br>- CGI-S
Martin 2016	39.3	Both	Schizophrenia Spectrum Disorder	IG: 16.1<br>CG: 15.6	TAU (N=24)	Movement therapy (N=44)	90min/day, 2x/wk	10 weeks	- SAPS<br>- SANS<br>- BPRS<br>- GAF
Marzolini 2009	44.9	Both	Schizophrenia or schizoaffective disorder	-	TAU (N=6)	Aerobic exercise (N=7)	90min/day, 2x/week	12 weeks	- MHI-18

Oertel Knöchel 2014	39.7	Both	Schizophrenia	Minimum of 5	- Relaxation + cognitive training (N=11)<br>- Waitlist (N=10)	Aerobic + cognitive training (N=8)	75 min (30min cognitive training + 45 min aerobic/relaxation training), 3x/week	4 weeks	- MATRICS cognitive battery<br>- STAI<br>- SF-12 (PSK)<br>- PANSS<br>- RHS
Paikkatt 2012	20-50	Male	Schizophrenia	Minimum of 2	TAU (N=15)	Yoga (N=15)	90min/day, 5x/week	1 month	- GWBM<br>- IDEAS<br>- Basic living skills
Pajonk 2010	33.9	Male	Chronic schizophrenia	10.4	SZ patients: table football (N=11)<br>Healthy controls: aerobic exercise (N=8)	Aerobic exercise (N=11)	30min/day, 3x/week	12 weeks	- Cognition<br>- MRI: hippocampal volume, total brain volume, gray matter volume
Scheewe 2013a[29]	29.4	Both	Schizophrenia, schizoaffective or schizophreniform disorder	6-7.9	Healthy controls: exercise therapy (N=27) or life as usual (N=28)<br>SZ patients: occupational therapy (N=32)	Cardiorespiratory & strength exercises (N=31)	60min/day (40min cardiorespiratory, 20min strength exercise), 2x/week	6 months	- MRI: global brain volume, hippocampal volume, cortical thickness
Scheewe 2013b[29]	29.7	Both	Schizophrenia, schizoaffective or schizophreniform disorder	IG: 6.3<br>CG: 6.96	Occupational therapy (N=32)	Cardiovascular + strength training (N=31)	60min/day, 2x/week	6 months	- PANSS<br>- MADRS
Svatkova 2015	29.2	Both	Schizophrenia	-	Healthy controls: exercise (N=24), life as usual (N=24)<br>SZ patients: occupational therapy (N=17)	Aerobic & anaerobic exercise (N=16)	60min/day (40min aerobic, 20 min anaerobic exercise), 2x/week	6 month	- MRI (DTI): structural connectivity
Ulloa 2013	15.1	Both	Schizophrenia or schizophreniform disorder		TAU (N=12)	Exercise (N=7)	30min, 9 times		- PANSS<br>- CGI-SCH-S<br>- PSP
Varambally 2012	32.2	Both	Schizophrenia	-	Waitlist (N=36)	- Yoga (N=47)<br>- Exercise (N=37)	- 45min/day, 25 days by yoga instructor<br>- 3 months of yoga	4 months	- PANSS<br>- SOFS

							at home		
Visceglia 2011	42.8	Both	Schizophrenia (some with additional schizoaffective disorder or PTSD)	-	Waitlist (N=8)	Yoga (N=10)	45min/day, 2x/week	8 weeks	- PANSS<br>- WHOQOL-BREF
Zwick 2010	32.1	Both	Paranoid schizophrenia	IG: 6.25<br>CG: 4.82	Occupational therapy (N=17)	Endurance training (N=20)	30min/day, 4x/week	-	- Cognition
**Unipolar Depression**									
Babyak 2000[3]	57.0	Both	MDD	-	- MDD: medication (N=48)	- Aerobic exercise (N=53)<br>- Medication + exercise (N=55)	45min/day, 3x/wk, for 16 weeks	10 months	- HDRS<br>- BDI<br>- DIS
Blumenthal 1999	57.0	Both	MDD	-	- MDD: medication (N=48)	- Aerobic exercise (N=53)<br>- Medication + exercise (N=55)	45min/day, 3x/wk	16 weeks	- HDRS<br>- BDI<br>- STAI<br>- RSES<br>- Life satisfaction<br>- Dysfunctional attitude
Blumenthal 2007	52.0	Both	MDD	-	- MDD: sertraline (N=49)<br>- MDD: placebo (N=49)	- Supervised aerobic exercise (N=51)<br>- Home-based aerobic exercise (N=53)	45min/day, 3x/wk	16 weeks	- HDRS<br>- BDI
Brenes 2007	74.6	Both	Minor depression	-	- Depression: sertraline (N=11)<br>- Depression: usual care (N=12)	Aerobic + resistance exercise (N=14)	60min/day, 3x/wk	16 weeks	- HDRS<br>- GDS<br>- SF-36<br>- Physical functioning
Buschert 2018	47.4	Both	Unipolar depression		- Depression: occupational therapy (N=20)	Physical exercise (N=18)	30min/day, 2-3x/week	3-4 weeks	- Cognition<br>- BDI<br>- HDRS
Carneiro 2015	50.2	Female	Clinical depression	-	Depression: usual pharmacotherapy (N=13)	Aerobic exercise + pharmacotherapy (N=13)	45-50min, 3x/wk	16 weeks	- BDI<br>- DASS-21<br>- RSES<br>- 6MWT<br>- 30CST
Carneiro 2016[4]	50.2	Female	Clinical depression		Depression: usual pharmacotherapy (N=13)	Aerobic exercise + pharmacotherapy (N=13)	45-50min, 3x/wk	16 weeks	- Blood monoamines levels<br>- Blood cortisol level
Carta 2008[5]	40-60	Female	MDD	-	MDD: pharmaco-therapy (N=20)	Strengthening exercises (N=10)	60min/day, 2x/wk	8 months	- WHOQOL-BREF

Carter 2015	15.4	Both	Depression	-	Depression: TAU (N=43)	Strengthening + aerobic exercises (N=44)	60min,day, 2x/wk, for 6 weeks	6 months	- RPE<br>- CDI-2<br>- EQ-5D
Cecchini-Estrada 2015	19.6	Both	Depression	-	- Depression: physical activity TARGET strategies program with teacher (N=27)<br>- Depression: physical activity without TARGET strategies program (N=27)<br>- Depression: stretching (N=26)	Aerobic exercise (N=26)	60min/day, 3x/wk	8 weeks	- Depressive symptoms<br>- Motivation
Chan 2011	49.3	Both	Depression	-	Depression: CBT (N=20)	DMBI (N=20)	90min/wk	4 weeks	- BDI<br>- EEG
Chan 2012	46.5	Both	MDD	IG: 9.2<br>CG (CBT): 7.2<br>CG (waitlist): 10.4	MDD: CBT (N=17)<br>MDD: waitlist (N=16)	DMBI (N=17)	90min/wk	10 weeks	- HDRS<br>- BDI<br>- Concentration / Attention
Chan 2013[6]	46.5	Both	MDD	-	- MDD: CBT (N=17)<br>- MDD: waitlist (N=16)	DMBI (N=17)	90min/wk	10 weeks	- EEG
Chou 2004	72.6	Both	Major depression or dysthemia	-	Depression: usual care (N=7)	Tai Chi (N=7)	45min/day, 3x/wk	12 weeks	- CES-D
Combs 2014[7]	52.0	Both	MDD	-	- MDD: sertraline (N=49)<br>- MDD: placebo (N=49)	- Supervised aerobic exercise (N=51)<br>- Home-based aerobic exercise (N=53)	45min/day, 3x/wk	16 weeks	- Sleep disturbance
Danielsson 2014	45.5	Both	Major depression	-	- Depression: BBAT (N=20)<br>- Depression: advice on physical activity (N=20)	Aerobic exercise (N=22)	60min/day, 2x/wk	10 weeks	- MADRS<br>- GAF<br>- BAI<br>- Body awareness
Doose 2015	48.7	Both	MDD	IG: 8.1<br>CG: 10.8	MDD: TAU (N=16)	Aerobic exercise (N=30)	60min/day, 3x/wk	8 weeks	- HDRS<br>- BDI<br>- RPE
Dunn 2005	35.9	Both	Mild to moderate MDD	-	MDD: stretching exercises (N=13)	- Low-dose treadmill training 3x/wk (N=16)<br>- High-dose treadmill training 3x/wk (N=17)<br>- Low-dose treadmill training 5x/wk (N=18)<br>- High-dose treadmill training 5x/wk (N=16)	3-5x/wk	12 weeks	- HDRS

Hoffman 2008[8]	51.7	Both	MDD	-	- MDD: sertraline (N=49)<br>- MDD: placebo (N=49)	- Supervised aerobic exercise (N=51)<br>- Home-based aerobic exercise (N=53)	45min/day, 3x/wk	16 weeks	- Cognition
Hoffman 2011[9]	51.7	Both	MDD	-	- MDD: sertraline (N=49)<br>- MDD: placebo (N=49)	- Supervised aerobic exercise (N=51)<br>- Home-based aerobic exercise (N=53)	45min/day, 3x/wk, for 16 weeks	1 year	- HDRS<br>- MDD status
Huang 2015	76.5	Both	Elderly with depressive symptoms	-	- Depression: CBT (N=18)<br>- Depression: TAU (N=20)	Physical fitness exercise (N=19)	50min/day, 3x/wk, for 12 weeks	9 months	- GDS<br>- 6MWT<br>- SF-36
Hughes 2013	17.0	Both	MDD	-	MDD: stretching exercises (N=14)	Aerobic exercise (N=16)	30-40min/day, 3x/wk, for 12 weeks	52 weeks	- CDI-revised<br>- CGI<br>- C-GAS<br>- QIDS
Kerling 2015	42.6	Both	MDD	-	MDD: TAU (N=20)	Aerobic exercise (N=22)	45min/day, 3x/wk	6 weeks	- MADRS<br>- BDI
Kerling 2017[37]	42.6	Both	MDD	-	MDD: TAU (20)	Aerobic exercise (N=22)	45min/day, 3x/wk	6 weeks	- BDNF
Kerling 2018[37]	41.0	Both	MDD	-	MDD: TAU (10)	Aerobic exercise (N=20)	45min/day, 3x/wk	6 weeks	- Muscle mass
Kerse 2010	81.1	Both	Elderly with depressive symptoms	-	Depression: social visits (N=96)	Home-based physical activity (balance, aerobic, resistance) (N=97)	30min/day, 3x/wk, for 6 months	12 months	- ADL<br>- SF-36<br>- GDS
Khatri 2001[10]	56.7	Both	MDD	-	- MDD: medication (N=42)	Aerobic exercise (N=42)	45min/day, 3x/wk	16 weeks	- HDRS<br>- BDI<br>- Cognition
Kinser 2013	43.3	Female	MDD	-	MDD: health education (N=12)	Yoga (N=15)	75min/wk	8 weeks	- PHQ-9<br>- STAI<br>- PSS-10<br>- RSS
Kinser 2014[11]	43.3	Female	MDD	-	MDD: health education (N=12)	Yoga (N=15)	75min/wk, for 8 weeks	1 year	- PHQ-9<br>- STAI<br>- PSS-10<br>- RSS<br>- SF-12
Knubben 2007	49.5	Both	MDD	-	MDD: stretching + relaxation exercises (N=18)	Endurance exercise (N=20)	30min/day	10 days	- BRMS<br>- CES-D

Krogh 2009	38.9	Both	Depression	-	Depression: relaxation exercises (N=55)	- Strength training (N=55)<br>- Aerobic training (N=55)	90min/day, 2x/wk, for 4 months	12 months	- HDRS<br>- Cognition
Krogh 2012	41.6	Both	MDD	-	MDD: stretching exercises (N=59)	Aerobic exercise (N=56)	45min/day, 3x/wk	12 weeks	- HDRS<br>- BDI<br>- Anxiety<br>- Cognition
Lavretsky 2011	70.6	Both	MDD	IG: 3.0<br>CG: 2.9	MDD: health education (N=37)	Tai Chi (N=36)	120 min/wk	10 weeks	- HDRS<br>- Cognition<br>- CGI<br>- HAS<br>- SF-36<br>- Apathy<br>- UPDRS<br>- CRP
Legrand 2015	23.0	Female	Depression	-	Depression: waitlist (N=22)	Aerobic exercise (N=22)	60min/day, 2x/wk	7 weeks	- BDI<br>- Self-esteem
Legrand 2016	45.3	Both	MDD	-	- MDD: stretching (N=11)<br>- MDD: TAU (N=10)	Aerobic exercise (N=14)	30min/day	10 days	- BDI
Luttenberger 2015	44.0	Both	Depression	-	Depression: waitlist (N=26)	Bouldering (N=25)	180min/wk	8 weeks	- BDI<br>- SCL-90<br>- Attention & concentration
Martiny 2015	47.7	Both	MDD	-	MDD: wake therapy (N=37)	Exercise (N=38)	30min/day for 8 weeks	29 weeks	- HDRS<br>- GAF<br>- Sleep
Mather 2002	65.0	Both	Depression	-	Depression: health education (N=43)	Endurance and strengthening exercises (N=43)	45min/day, 2x/wk, for 10 weeks	35 weeks	- HDRS<br>- GDS<br>- CGI
Mota-Pereira 2011	47.0	Both	MDD	-	MDD: pharmacotherapy (N=11)	Home-based walking (N=22)	30-45min/day, 5x/wk	12 weeks	- HDRS<br>- BDI<br>- CGI<br>- GAF
Murri 2015	75.0	Both	MDD	-	- MDD: sertraline (N=42)	- Progressive aerobic exercise (N=42)<br>- Non-progressive exercise (N=37)	60min/day, 3x/week	24 weeks	- HDRS<br>- CGI
Nabkasorn 2006	18.8	Female	Mild to moderate depression	-	Depression: usual care (N=31)	Jogging (N=28)	50min/day, 5x/week	8 weeks	- CES-D<br>- Urine cortisol level<br>- Urine epinephrine level

Niemi 2016	61.3	Both	Moderate depression	-	Depression: TAU (N=22)	Yoga + psycho-education (N=34)	1x/week	8 weeks	- PHQ-9
Oertel Knöchel 2014	40.0	Both	MDD	IG: 10.0<br>CG (relaxation): 10.1<br>CG (waitlist): 11.0	- MDD: relaxation + cognitive training (N=6)<br>- MDD: waitlist (N=8)	Aerobic + cognitive training (N=8)	75 min (30min cognitive training + 45 min aerobic/relaxation training), 3x/week	4 weeks	- BDI<br>- STAI<br>- SF-12<br>- Cognition
Olson 2017	21.1	Both	MDD	-	MDD: stretching (N=21)	Aerobic exercise (N=21)	45min/day, 3x/week	8 weeks	- BDI<br>- Cognition<br>- EEG<br>- Cardiorespiratory fitness
Pfaff 2014	61.0	Both	Major or minor depression		Depression: TAU (N=92)	Home-based aerobic + resistance exercises (N=108)	30min/day, 5x/week, for 12 weeks	52 weeks	- SIGMA<br>- SCID-I
Pilu 2007	40-60	Female	MDD	-	MDD: pharmacotherapy (N=20)	Strengthening exercises (N=10)	60min/day, 2x/week	8 months	- HDRS<br>- CGI<br>- GAF
Prakhinkit 2014	76.6	Both	Mild-to-moderate depression	-	Depression: usual care (N=15)	- Aerobic walking exercise (15)<br>- Buddhism walking meditation (N=15)	20-30min/day, 3x/week	12 weeks	- GDS<br>- IL-6<br>- Blood cortisol<br>- CRP
Prathikanti 2017	43.4	Both	MDD	-	MDD: education on yoga (N=12)	Yoga (N=18)	90min/day, 2x/week	8 weeks	- BDI<br>- Cognition<br>- Self-efficacy
Schuch 2014	42.7	Both	MDD	-	MDD: TAU (N=11)	Aerobic exercise (N=15)	3x/week	-	- TBARS<br>- Serum BDNF
Schuch 2015	40.3	Both	Severe MDD	-	MDD: TAU (N=25)	Aerobic exercise (N=25)	3x/week	-	- HDRS<br>- WHOQOL BREF
Shahidi 2011	66.5	Female	Depression	-	- Depression: usual care (N=24)	- Aerobic exercise (N=23)<br>- Laughter yoga (N=23)	30min/session, total 10 session	-	- GDS<br>- Life satisfaction
Sharma 2017	37.1	Both	MDD	-	MDD: waitlist (N=12)	Yoga (N=13)	Week 1: 3.5h/day, week 2-8: 90min/week + 20-25/day at home	8 weeks	- HDRS<br>- BDI<br>- BAI<br>- Suicidal ideation
Sims 2006	74.3	Both	Depression	-	Depression: advice (N=18)	PRT (N=14)	3x/week	10 weeks	- GDS<br>- CES-D<br>- HAP<br>- PGMS<br>- WHOQOL-BREF

Singh 1997a	71.0	Both	Unipolar major or minor depression or dysthemia	IG: 2.0<br>CG: 2.7	Depression: Health education (N=13)	PRT (N=15)	60min/day, 3x/wk	10 weeks	- Sleep
Singh 1997b	71.0	Both	Unipolar major or minor depression or dysthemia	IG: 2.0<br>CG: 3.0	Depression: Health education (N=15)	PRT (N=17)	50min/day, 3x/wk	10 weeks	- BDI<br>- GDS<br>- PGMS<br>- HDRS<br>- SF-36<br>- IADL
Singh 2001[12]	71.0	Both	Unipolar major or minor depression or dysthemia	-	Depression: Health education (N=14)	PRT (N=15)	45min/day, 3x/wk, for 10 weeks	26 months	- BDI<br>- PGMS
Singh 2005	69.3	Both	Unipolar major or minor depression or dysthemia	-	Depression: usual care (N=20)	- High-intensity PRT (N=20)<br>- Low-intensity PRT (N=20)	60min/day, 3x/wk	8 weeks	- GDS<br>- HDRS<br>- SF-36<br>- PSQI
Siqueira 2016	38.8	Both	MDD	-	MDD: usual care (N=28)	Aerobic exercise (N=29)	4x/week	4 weeks	- HDRS<br>- BDI
Tolahunase 2018	-	-	MDD	-	MDD: TAU (N=-)	Yoga (N=-)	-	12 weeks	- BDI<br>- BDNF
Tsang 2006	82.4	Both	Depression	-	Depression: newspaper reading group (N=34)	Qigong exercise (N=48)	30-45min/day, 3x/week, for 16 weeks	24 weeks	- GDS<br>- Self-efficacy<br>- GHQ-12<br>- PWI
Tsang 2012	80.2	Both	MDD	-	MDD: newspaper reading group (N=17)	Qigong exercise (N=21)	45min/day, 3x/week, for 12 weeks	20 weeks	- GDS<br>- HDRS<br>- Self-efficacy<br>- Muscle strength<br>- Blood serotonin level<br>- Salivary cortisol
Veale 1992	35.5	Both	Depression	-	Depression: usual care (N=35)	Aerobic exercise (N=48)	3x/week	12 weeks	- BDI<br>- Anxiety<br>- CIS
Yeung 2012	55.0	Both	MDD	-	MDD: waitlist (N=13)	Tai Chi (N=26)	60min/day, 2x/week	12 weeks	- HDRS<br>- CGI<br>- QoL
Yeung 2017	54.0	Both	MDD	-	MDD: education (N=22)<br>MDD: waitlist (N=22)	Tai Chi (N=23)	60min/day, 2x/week, for 12 weeks	24 weeks	- HDRS<br>- SF-36<br>- CGI<br>- Mindfullness

-: not available; [1] Same study as Cox 2015, but with different outcome measures reported; [2] Same study as Ohman 2016, but with results on physical function and mobility; [3] 10-month follow-up results of Blumenthal 1999; [4] Same study as Carneiro 2015 with results on blood monoamines levels; [5] Same study as Pilu 2007, but with results of QoL; [6] Same study as Chan 2012 with EEG results; [7] Same study as Blumenthal 2007 with results on sleep; [8] Same study as Blumenthal 2007 with results on cognition; [9] 1-year follow-up results of Blumenthal 2007; [10] Same study as Blumenthal 1999 with results on cognition; [11] 1-year follow-up results of Kinser 2013 and with also reporting 8-weeks outcome on PHQ-39; [12] Phase II results of Singh 1997b; [13] Same study as Ahmadi 2010a and 2010b, but with results on BDI and BAI; [14] Same study as Wens 2015b with results on inflammatory markers; [15] Follow-up study of Garrett 2012a; [16] Same study as Garrett 2012a, and Garrett 2012b, but with different MS patients; [17] Same study as Kargarfard 2012, but with different outcome measures reported; [18] Same study as Romberg 2004, but with different outcome measures reported; [19] Same study as Wens 2015b with result son serum BDNF levels; [20] Same study as Wens 2015a with results on glucose tolerance; [21] Same study as Corcos 2013, but with different outcome measures reported; [22] Same studies, but with results reported at different follow-up periods; [23] Same study as Harro 2014a, but with different outcome measures; [24] Same studies with different outcome measures reported in the two papers; [25] Secondary analyses of Ni 2015; [26] Same study as Colgrove 2012 but with different outcome measures reported; [27] Same studies, but with different outcome measures reported; [28] 12-month follow-up study of Wong-Yu 2015a; [29] Same studies, but with different outcome measures; [30] Same studies, but with different outcome measures; [31] Same studies, but with different outcome measures; [32] Same studies, but with different outcome measures; [33] Same study as Kargarfard 2012, but with larger sample size; [34] Same study as Sandroff 2016, but with different outcome measures; [35] Same study as Silva-Batista 2016, but with different outcome measures; [36] Same study as Lin 2015, but with different outcome measures; [37] Same study as Kerling 2017, but woth different outcome measures

2-MST: 2-Minute Step Test; 2MWT: 2-Minute Walk Test; 3MWT: 3-Minute Walk test; 6MWT: 6-Minute Walk Test; 8-FT: 8-Foot Up and Go Test; 9-HPT: 9Hole Peg Test; 10mWT: 10-m Walk Test; 30CST: 30-second Chair Stand Test; 50mWT: 50-meter Walk Test; 500mWT: 500-meter Walk Test; ABC: Activities-specific Balance Confidence scale; ACIF: Acute Care Index of Function; ACS: Activity Card Sort; ACT: Arm Curl Test; AD: Alzheimer's disease; ADL: Activities of Daily Living; ALDS: Academic Medical Center linear disability score; BAI: Beck Anxiety Inventory; BBAT: Basic Body Awareness Therapy; BBS: Berg Balance Scale; BDI: Beck Depression Inventory; BDNF: Brain-derived neurotrophic factor; B'DS: Brown's Disability Scale; BESTest: Balance Evaluation Systems Test; Borg RPE scale: Borg Ratings of Perceived Exertion scale; BPRS: Brief Psychiatric Rating Scale; BRMS: Bech-Rafaelsen Melancholy Scale; BSI: Brief Symptom Inventory; BST: Back Scratch Test; CAHAI-8: Chedoke Arm and Hand Activity Inventory; CBT: Cognitive Behavioral Therapy; CDI-2: Children's Depression Inventory 2; CDSS: Calgary Depression Scale for Schizophrenia; CES-D: Center for Epidemiologic Studies Depression rating scale; CG: Control Group; C-GAS: Children's Global Assessment Scale; CGI: Clinical Global Impression; CGI-C: Clinical Global Impression of Change; CGI-S: Clinical Global Impression Severity scale; CGI-SCH: Clinical Global Impression-Schizophrenia; CIMT: Constraint-Induced Movement Therapy; CIS: Clinical Interview Schedule; CIS: Clinically Isolated Syndrome (in MS); CRP: C-reactive protein; CRT: Coin Rotation Task; CSF: CerebroSpinal Fluid; CSRT: Choice stepping reaction time; CSRT: Chair Sit and Reach Test; CST: Chair Stand Test; CWS: Comfortable Walking Speed; DASS-21: Depression Anxiety Stress Scale-21; DGI: Dynamic Gait Index; DHI: Dizziness Handicap Inventory; DIP: Disability and Impact Profile; DIS: Diagnostic Interview Schedule; DMBI: Dejian Mind-Body Intervention; dTUG: Dual-task Timed Up and GO test; EDSS: Expanded Disability Status Scale; EQ-5D: EuroQol health-related quality of life; EQ-5D VAS: EuroQoL 5D Visual Analogue Scale; FAB: Fullerton Advanced Balance scale; FAMS: Functional Assessment of MS; FES: Falls Efficacy Scale; FES-I: Falls Efficacy Scale-International questionnaire; FGA: Functional Gait Assessment; FIM: Functional Independence Measure; fMRI: Functional MRI; FOG: Freezing of Gait questionnaire; FR: Functional Reach; FRT: Functional Reach test; FSMC: Fatigue Scale for Motor and Cognitive functions; FSS: Fatigue Severity Scale; FSST: Four Square Step Test; FST: Functional Stair Test; FTSTS: Five Times Sit-To-Stand; GAF: Global Assessment of Functioning scale; GDS: Geriatric Depression Scale; GFQ: Gait and Falls Questionnaire; GHQ-12: General Health Questionnaire 12; GHQ-28: General Health Questionnaire-28; GLTEQ: Godin Leisure Time Exercise Questionnaire; GNDS: Guy's Neurological Disability Scale; GWBM: Post Graduate Institute general well-being measure; HADS: Hospital Anxiety and Depression Scale; HAP:

Human Activities Profile; HARS: Hamilton Anxiety Rating Scale; HAS: Hamilton Anxiety Scale; HAQUAMS: Hamburg Quality of Life Questionnaire in MS; HD: Huntington's Disease; HDQoL: Huntington's disease Health Related Quality of Life questionnaire; HDRS: Hamilton Depression Rating Scale; HR-QoL: Health-related Quality of Life; HY-stage: Hoehn and Yahr staging; IADL: Instrumental Activities of Daily Life; IDEAS: Indian disability evaluation and assessment scale; IDS-C30: Inventory of Depression Symptomatology-Clinician Rated; IFN-γ : Interferon-gamma; IG: Intervention Group; IGF-1: Insulin-like Growth Factor 1; IIQ: Incontinence Impact Questionnaire; IL-1β: Interleukin-1 beta; IL-4: Interleukine-4; IL-6: Interleukin-6; IL-17: Interleukine-17; IPA: Impact on Participation and Autonomy; iTBS: intermittent Theta Burst Stimulation; LAPAQ: Longitudinal aging study Amsterdam physical activity questionnaire; Lehman QoL: Lehman quality of Life Short Form TL30S; LOS: Limit Of Stability test; LR: Lateral Reach; LSSI: Lipp's Stress Symptoms Inventory; LSVT BIG therapy: Lee Silverman Voice Therapy BIG; MADRS: Montgomery Asberg depression Rating Scale; MAS: Modified Ashworth Scale for spasticity; MCP: Multicomponent cognitive program; MCT: Motor Control Test; MDD: Major Depressive Disorder; MDS-UPDRS I: Movement Disorder Society-Unified Parkinson's Disease Rating Scale Non-motor experiences; MDS-UPDRS III: Movement Disorder Society-Motor subscore of the Unified Parkinson's Disease Rating Scale; MDS-UPDRS-ADL (i.e. UPDRS-II): Movement Disorder Society-Activities of Daily Living assessed with second part of UPDRS; MEP: Motor Evoked Potential; mFC: modified Fitness counts exercise program focused on stretching, balance, breathing and non-progressive strengthening; MFE: Modified Figure-of-Eight test; MFI: Multidimensional Fatigue Inventory; MFIS: Modified Fatigue Impact Scale; MHI-18: 18-item Mental Health Inventory; MMSE: Mini-Mental State Examination; MoCA: Montreal Cognitive Assessment; M-PAS: Modified Parkinson Activity Scale; mPPT: modified Physical Performance Test; MPQ: McGill Pain Questionnaire; MS: Multiple Scelrosis; MRE: Magnetic Resonance Elastography; MSFC: MS Functional Composite (includes cognition); MSIS-29: MS Impact Scale-29; MSQOL-54: MS Quality of Life-54; MSSE: MS Self-Efficacy Scale; MSSS-88: MS Spasticity Scale-88; MSWS-12: MS 12-item walking scale; NFL: Neurofilament light; Ng: Neurogranin; NGF: Nerve Growth Factor; NHPB: Nine hole peg board test; NMSS: Non-motor Symptoms Scale; NPI: Neuropsychiatric Inventory; OGTT: Oral Glucose Tolerance Test; OLS: 1-Leg Stance; PANAS: Positive and Negative Affect Schedule; PANSS: Positive and Negative Syndrome Scale; PAS: Parkinson Activity Scale; PASE: Physical Activity Scale for the Elderly; PD: Parkinson's disease; PDDS: Parkinson's Disease Disability Scale; PDQ-8: Parkinson's Disease Questionnaire-short version; PDQ-39: Parkinson's Disease Questionnaire; PDQL: Parkinson's Disease Quality of Life scale; PFS: Parkinson's Fatigue Scale; PGMS: Philadelphia Geriatric Morale Scale; Phone-FITT: household and recreational physical activity interview; PHQ-9: Patient Health Questionnaire 9; POMA: Tinetti Performance-Oriented Mobility Assessment; POMS: Profile of Mood States; PPA: Physiological Profile Assessment; PP-MS: Primary Progressive MS; PPOS: Patient Preference Outcome Scale; PRET: Progressive Resistance Exercise Training of major muscle groups; PRT: Progressive Resistance Training; PS: Postural Sway test; PSI-PD: Patient-specific index for Parkinson's disease; PSP: Personal and Social Performance Scale; PSQI: Pittsburgh Sleep Quality Index; PSS-10: Perceived Stress Scale-10; PWI: Personal Well Being Index; PWSSTL Partial-weight-supported treadmill gait training; QIDS: Quick Inventory for Depression Symptomatology; QLDS: Quality of Life in depression Scale; QoL: Quality of Life; RAGT: Robot-Assisted Gait Training; RHS: Revised Hallucination Scale; RMDQ: Roland Morris Disability Questionnaire; RMI: Rivermead Mobility Index; RPE: Rating of Perceived Exertion Scale; RR-MS: Relapse remitting MS; RSES: Rosenberg Self Esteem Scale; RSS: Ruminative Responses Scale; RST: Rapid Step-Up Test; rTMS: repetitive Transcranial Magnetic Stimulation; SAFE: Sensory-attention focused exercise; SANS: Scale for the Assessment of Negative Symptoms; SAPS: Scale for the Assessment of Positive Symptoms; SAS: Self-assessment Parkinson's Disease Disability Scale; SCID-I: Structured Clinical Interview for DSM-IV Axis I Disorders; SCL-90: Symptom Checklist-90-R; SCT: Stair Climbing Test; SEES: Subjective Exercise Experiences Scale; SF-6D: Short Form 6 dimensions utility score; SF-12: 12-item Short Form Health Survey; SF-12 MCS: Mental Health Composite Score of SF-12; SF-12 PCS: Physical Health Composite Score of SF-12; SF-36: 36-item Short Form Health Survey; SGI: Subject Global Impression; SIGMA: Structured Interview Guide for the Montgomery-Asberg depression Rating Scale; SIP-68: short version of Sickness Impact Profile for measuring health-related functional status; SLS: Single Leg Stance test; sMRI: Structural MRI; SOFS: Social and occupational Functioning Scale; SOT: Sensory organization balance test; SP-MS: Secondary Progressive MS; SPPB: Short Physical Performance Battery; SRT: Sit and Reach Test; SSEP: Somatosensory Evoked Potential; SST: Stroop stepping test; STAI: State-Trait Anxiety Inventory; STS: Sit-to-Stand test; SWLS: Satisfaction with Life scale; SZ: Schizophrenia; T25FW: Timed 25 Foot Walk; TAU: Treatment as usual; TBARS: Thiobarbituric Acid-reactive substances; TCS: Timed Chair-Stand test; TIS: Trunk Impairment Scale; TMS: Transcranial Magnetic Stimulation; TRENDS: Tool for Recognition of Emotions in Neuropsychiatric Disorders; TUG: Timed Up and Go test; UHDRS: Unified Huntington's Disease Rating Scale; UPDRS:

Unified Parkinson's Disease Rating Scale; VAS: Visual Analogue Scale; VILIP-1: Visinin-like protein 1; VPS: Vitality Plus Scale; WBV: Whole Body Vibration; WHODAS: World Health Organization Disability Assessment Schedule; WHOQOL BREF: World Health Organization Quality of Life-BREF; YKL-40: Chitinase-3-like protein 1

References

1 Aguiar P, Monteiro L, Feres A, Gomes I, Melo A. Rivastigmine transdermal patch and physical exercises for Alzheimer's disease: a randomized clinical trial. *Curr Alzheimer Res* 2014; **11**: 532–7.

2 Arcoverde C, Deslandes A, Moraes H, *et al.* Treinamento na esteira como um tratamento adicional para a doen??a de Alzheimer: Estudo piloto controlado randomizado. *Arq Neuropsiquiatr* 2014; **72**: 190–6.

3 CA C, P D, S S, *et al.* The effects of a walking/talking program on communication, ambulation, and functional status in residents with Alzheimer disease. *Alzheimer Dis Assoc Disord* 2002; **16**: 81–7.

4 Cox KL, Flicker L, Almeida OP, *et al.* Effects of a 6-month, home-based, physical activity program on the fitness and health of adults with Alzheimer's disease: The fabs ii trial. *Alzheimer's Dement J Alzheimer's Assoc* 2015; **11**: P461.

5 Friedman R, Tappen RM. The effect of planned walking on communication in Alzheimer's disease. *J Am Geriatr Soc* 1991; **39**: 650–4.

6 Hoffmann K, Sobol NA, Frederiksen KS, *et al.* Moderate-to-high intensity physical exercise in patients with Alzheimer's disease: A randomized controlled trial. *J Alzheimer's Dis* 2015; **50**: 443–53.

7 Holthoff VA, Marschner K, Scharf M, *et al.* Effects of physical activity training in patients with alzheimer's dementia: Results of a pilot RCT study. *PLoS One* 2015; **10**: 1–12.

8 Kemoun G, Thibaud M, Roumagne N, *et al.* Effects of a physical training programme on cognitive function and walking efficiency in elderly persons with dementia. *Dement Geriatr Cogn Disord* 2010; **29**: 109–14.

9 Kim M-J, Han C-W, Min K-Y, *et al.* Physical Exercise with Multicomponent Cognitive Intervention for Older Adults with Alzheimer's Disease: A 6-Month Randomized Controlled Trial. *Dement Geriatr Cogn Dis Extra* 2016; **6**: 222–32.

10 Lautenschlager NT, Cox KL, Flicker L, *et al.* A randomized controlled trial evaluating the effects of physical activity in people with Alzheimer's disease: the fitness for the ageing brain study ii (fabs II). *Alzheimer's Dement* 2015; **11**: P280–1.

11 Maci T, Pira FL, Quattrocchi G, Nuovo SD, Perciavalle V, Zappia M. Physical and Cognitive Stimulation in Alzheimer Disease. The GAIA Project: A Pilot Study. *Am J Alzheimers Dis Other Demen* 2012; **27**: 107–13.

12 McCurry SM, Pike KC, Vitiello M V., Logsdon RG, Larson EB, Teri L. Increasing walking and bright light exposure to improve sleep in community-dwelling persons with Alzheimer's disease: Results of a randomized, controlled trial. *J Am Geriatr Soc* 2011; **59**: 1393–402.

13 ??hman H, Savikko N, Strandberg TE, *et al.* Effects of Exercise on Cognition: The Finnish Alzheimer Disease Exercise Trial: A Randomized, Controlled Trial. *J Am Geriatr Soc* 2016; **64**: 731–8.

14 Öhman H, Savikko N, Strandberg T, *et al.* Effects of exercise on functional performance and fall rate in subjects with mild or advanced Alzheimer's disease: Secondary analyses of a randomized controlled study. *Dement Geriatr Cogn Disord* 2016; **41**: 233–41.

15 Padala KP, Padala PR, Malloy TR, *et al.* Wii-fit for improving gait and balance in an assisted living facility: A pilot study. *J Aging Res* 2012; **2012**. DOI:10.1155/2012/597573.

16 Perea RD, Vidoni ED, Graves RS, Burns JM, Honea RA. The effects of aerobic exercise in Alzheimer’s disease thalamo-cortical connections using diffusion tensor imaging. *Alzheimer’s Dement J Alzheimer’s Assoc* 2015; **11**: P424.
17 Pitkala KH, Poysti MM, Laakkonen ML, *et al.* Effects of the Finnish Alzheimer Disease Exercise Trial (FINALEX): A randomized controlled trial. *JAMA Intern Med* 2013; **173**: 894–901.
18 Roach KE, Tappen RM, Kirk-Sanchez N, Williams CL, Loewenstein D. A randomized controlled trial of an activity specific exercise program for individuals with Alzheimer disease in long-term care settings. *J Geriatr Phys Ther* 2011; **34**: 50–6.
19 Rolland Y, Pillard F, Klapouszczak A, *et al.* Exercise program for nursing home residents with Alzheimer’s disease: A 1-year randomized, controlled trial. *J Am Geriatr Soc* 2007; **55**: 158–65.
20 Santana-Sosa E, Barriopedro MI, López-Mojares LM, Pérez M, Lucia A. Exercise training is beneficial for Alzheimer’s patients. *Int J Sports Med* 2008; **29**: 845–50.
21 Steinberg M, Leoutsakos JS, Podewils LJ, Lyketsos CG. Evaluation of a home-based exercise program in the treatment of Alzheimer’s disease: The Maximizing Independence in Dementia (MIND) study. *Int J Geriatr Psychiatry* 2009; **24**: 680–5.
22 Suttanon P, Hill KD, Said CM, *et al.* Feasibility, safety and preliminary evidence of the effectiveness of a home-based exercise programme for older people with Alzheimer’s disease: a pilot randomized controlled trial. *Clin Rehabil* 2012; **27**: 427–38.
23 RM T, KE R, EB A, *et al.* Effect of a combined walking and conversation intervention on functional mobility of nursing home residents with Alzheimer disease. *Alzheimer Dis Assoc Disord* 2000; **14**: 196–201.
24 Teri L, Gibbons LE, McCurry SM, *et al.* Exercise plus behavioral management in patients with Alzheimer disease: a randomized controlled trial. *Jama* 2003; **290**: 2015–22.
25 van der Kleij LA, Petersen ET, Siebner HR, *et al.* The effect of physical exercise on cerebral blood flow in Alzheimer’s disease. *Neuroimage Clin* 2018; **20**: 650–54.
26 Venturelli M, Scarsini R, Schena F. Six-month walking program changes cognitive and ADL performance in patients with Alzheimer. *Am J Alzheimers Dis Other Demen* 2011; **26**: 381–8.
27 Vreugdenhil A, Cannell J, Davies A, Razay G. A community-based exercise programme to improve functional ability in people with Alzheimer’s disease: A randomized controlled trial. *Scand J Caring Sci* 2012; **26**: 12–9.
28 Wong AMK, Chiu Y-T, Wang C-W, Pei Y-C, Hsu W-C. Application of Tai Chi 6-Form Sport Apparatus in Patients with Alzheimer. 台灣老年醫學暨老年學雜誌 2014; **9**: 45.
29 Yágüez L, Shaw KN, Morris R, Matthews D. The effects on cognitive functions of a movement-based intervention in patients with Alzheimer’s type dementia: A pilot study. *Int J Geriatr Psychiatry* 2011; **26**: 173–81.
30 Yang S-Y, Shan C-L, Qing H, *et al.* The effects of aerobic exercise on cognitive function of Alzheimer’s disease patients. *CNS Neurol Disord - Drug Targets* 2015; **14**: 1292–7.
31 Zhang Y-H, Lu S-P, Xu Y-N, Fu X, Huang Q. Effect of one-year rehabilitation training in patients with Alzheimer disease. *Chinese J Clin Rehabil* 2004; **8**: 6859–61.
32 Busse M, Quinn L, Debono K, *et al.* A randomized feasibility study of a 12-week community-based exercise program for people with Huntington’s disease. *J Neurol Phys Ther* 2013; **37**: 149–58.
33 Khalil H, Quinn L, van Deursen R, *et al.* What effect does a structured home-based exercise programme have on people with Huntington’s disease? A

randomized, controlled pilot study. *Clin Rehabil* 2013; **27**: 646–58.
34 Quinn L, Debono K, Dawes H, *et al.* Task-specific training in huntington disease: a randomized controlled feasibility trial. *Phys Ther* 2014.
35 Thompson JA, Cruickshank TM, Penailillo LE, *et al.* The effects of multidisciplinary rehabilitation in patients with early-to-middle-stage Huntington's disease: A pilot study. *Eur J Neurol* 2013; **20**: 1325–9.
36 Aghaie M, Alijani E, Majdi-Nassab N, Goharpey S, Abedi-Yekta AH, Babadi M. The effect of 8-week aerobic and resistance training programme on patients with multiple sclerosis. *Mult Scler* 2010; **16**: S179.
37 Ahmadi A, Arastoo AA, Nikbakht M. The effects of a treadmill training programme on balance, speed and endurance walking, fatigue and quality of life in people with multiple sclerosis. *Int Sport J* 2010; **11**.
38 Ahmadi A, Nikbakh M, Arastoo AA, Habibi A-H. The Effects of a Yoga Intervention on Balance, Speed and Endurance of Walking, Fatigue and Quality of Life in People with Multiple Sclerosis. *J Hum Kinet* 2010; **23**: 71–8.
39 Ahmadi A, Arastoo AA, Nikbakht M, Zahednejad S, Rajabpour M. Comparison of the Effect of 8 weeks Aerobic and Yoga Training on Ambulatory Function, Fatigue and Mood Status in MS Patients. *Iran Red Crescent Med J* 2013; **15**: 449–54.
40 Arazi H, Samami N, Dehghan M, Jafari A. The effect of eight-week concurrent aerobic-resistance training on aerobic power and functional capacity on young female patients with multiple sclerosis. *J Zanjan Univ Med Sci Heal Serv* 2016; **24**: 31–42.
41 Azimzadeh E, Hosseini MA, Nourozi K, Davidson PM. Effect of Tai Chi Chuan on balance in women with multiple sclerosis. *Complement Ther Clin Pract* 2015; **21**: 57–60.
42 Barrett CL, Mann GE, Taylor PN, Strike P. A randomized trial to investigate the effects of functional electrical stimulation and therapeutic exercise on walking performance for people with multiple sclerosis. *Mult Scler* 2009.
43 Bernhardt L, Jolk C, Marziniak M. Specific exercise training in patients with multiple sclerosis. *Mult Scler* 2011; **17**: S251–2.
44 Bernhardt L, Jolk C, Alcantara R, Platen P, Marziniak M, Weßling K. The effects of resistance training and physical activities in groups in comparison for the treatment of chronic fatigue in patients with multiple sclerosis. *Mult Scler* 2012; **18**: 247.
45 Bjarnadottir OH, Konradsdottir a D, Reynisdottir K, Olafsson E. Multiple sclerosis and brief moderate exercise. A randomised study. *Mult Scler* 2007; **13**: 776–82.
46 Braendvik SM, Koret T, Helbostad JL, *et al.* Treadmill Training or Progressive Strength Training to Improve Walking in People with Multiple Sclerosis? A Randomized Parallel Group Trial. *Physiother Res Int* 2015. DOI:10.1002/pri.1636.
47 Briken S, Gold SM, Patra S, *et al.* Effects of exercise on fitness and cognition in progressive MS: a randomized, controlled pilot trial. *Mult Scler* 2014; **20**: 382–90.
48 Broekmans T, Roelants M, Alders G, Feys P, Thijs H, Eijnde BO. Exploring the effects of a 20-week whole-body vibration training programme on leg muscle performance and function in persons with multiple sclerosis. *J Rehabil Med* 2010; **42**: 866–72.
49 Broekmans T, Roelants M, Feys P, *et al.* Effects of long-term resistance training and simultaneous electro-stimulation on muscle strength and functional mobility in multiple sclerosis. *Mult Scler* 2011; **17**: 468–77.
50 Bulguroglu I, Guclu-Gunduz A, Gokhan Y, *et al.* Comparison of the effects of mat Pilates and reformer Pilates on balance, strength, mobility, fatique and quality of life in patients with multiple sclerosis. *Eur J Neurol* 2015; **22**: 672.
51 Çakt BD, Nacir B, Genç H, *et al.* Cycling progressive resistance training for people with multiple sclerosis: A randomized controlled study. *Am J Phys Med Rehabil* 2010; **89**: 446–57.
52 Carter A, Daley A, Humphreys L, *et al.* Pragmatic intervention for increasing self-directed exercise behaviour and improving important health outcomes

in people with multiple sclerosis: a randomised controlled trial. *Mult Scler* 2014; **20**: 1112–22.
53 Castro-S??nchez AM, Matar??n-Pe??arrocha GA, Lara-Palomo I, Saavedra-Hern??ndez M, Arroyo-Morales M, Moreno-Lorenzo C. Hydrotherapy for the treatment of pain in people with multiple sclerosis: A randomized controlled trial. *Evidence-based Complement Altern Med* 2012; **2012**. DOI:10.1155/2012/473963.
54 Catena L, Bacci L, Lombrano D, Morici C, Morgantini A. The effects of Pilates-fisios on pain, motor fatigue, and quality of life in patients with multiple sclerosis: A randomized controlled clinical trial. *Mult Scler* 2014; **20**: 982.
55 Cattaneo D, Jonsdottir J, Zocchi M, Regola A. Effects of balance exercises on people with multiple sclerosis: A pilot study. 2007; **21**: 771–81.
56 Claerbout M, Gebara B, Ilsbroukx S, *et al.* Effects of 3 weeks' whole body vibration training on muscle strength and functional mobility in hospitalized persons with multiple sclerosis. *Mult Scler J* 2012; **18**: 498–505.
57 Conklyn D, Stough D, Novak E, Paczak S, Chemali K, Bethoux F. A Home-Based Walking Program Using Rhythmic Auditory Stimulation Improves Gait Performance in Patients With Multiple Sclerosis: A Pilot Study. *Neurorehabil Neural Repair* 2010; **24**: 835–42.
58 Kerry Raivel PT, Raina Wilson PT MS. The effects of a twelve-week home walking program on cardiovascular parameters and fatigue perception of individuals with multiple sclerosis: a pilot study. *Cardiopulm Phys Ther J* 2009; **20**: 5.
59 Dalgas U, Stenager E, Jakobsen J, *et al.* Resistance training improves muscle strength and functional capacity in multiple sclerosis. *Neurology* 2009; **73**: 1478–84.
60 Dalgas U, Stenager E, Jakobsen J, Petersen T, Overgaard K, Ingemann-Hansen T. Muscle fiber size increases following resistance training in multiple sclerosis. *Mult Scler* 2010; **16**: 1367–76.
61 Dalgas U, Stenager E, Jakobsen J, *et al.* Fatigue, mood and quality of life improve in MS patients after progressive resistance training. *Mult Scler* 2010; **16**: 480–90.
62 Dalgas U, Stenager E, Lund C, *et al.* Neural drive increases following resistance training in patients with multiple sclerosis. *J Neurol* 2013; **260**: 1822–32.
63 N. D, I. W, A.H. N, *et al.* 12 Weeks of Combined Endurance and Resistance Training Reduces Innate Markers of Inflammation in a Randomized Controlled Clinical Trial in Patients With Multiple Sclerosis. *Mediators Inflamm* 2016; **2016**: no pagination.
64 DeBolt LS, McCubbin JA. The effects of home-based resistance exercise on balance, power, and mobility in adults with multiple sclerosis. *Arch Phys Med Rehabil* 2004; **85**: 290–7.
65 de Oliveira G, Tavares MDCCGF, de Faria Oliveira JD, Rodrigues MR, Santaella DF. Yoga Training Has Positive Effects on Postural Balance and Its Influence on Activities of Daily Living in People with Multiple Sclerosis: A Pilot Study. *Explor J Sci Heal* 2016; **12**: 325–32.
66 Dettmers C, Sulzmann M, Ruchay-Plössl A, Gütler R, Vieten M. Endurance exercise improves walking distance in MS patients with fatigue. *Acta Neurol Scand* 2009; **120**: 251–7.
67 Dodd KJ, Taylor NF, Shields N, Prasad D, McDonald E, Gillon A. Progressive resistance training did not improve walking but can improve muscle performance, quality of life and fatigue in adults with multiple sclerosis: a randomized controlled trial. *Mult Scler* 2011; **17**: 1362–74.
68 Doulatabad N, Tradit AJ, Altern C. Afr J Tradit Complement Altern Med. (2013). 2013; **10**: 49–52.
69 Najafidoulatabad S, Mohebbi Z, Nooryan K. Yoga Effects on Physical Activity and Sexual Satisfaction Among Iranian Women With Multiple Sclerosis: a Randomized Controlled Trial. *African J Tradit Complement Altern Med* 2014; **11**: 78–82.
70 Ebrahimi A, Eftekhari E, Etemadifar M. Effects of whole body vibration on hormonal & functional indices in patients with multiple sclerosis. *Indian J Med Res* 2015; **142**: 450–8.

71 Eftekhari E, Mostahfezian M, Etemadifar M, Zafari A. Resistance training and vibration improve muscle strength and functional capacity in female patients with multiple sclerosis. *Asian J Sports Med* 2012; **3**: 279–84.
72 Eftekharsadat B, Babaei-Ghazani A, Mohammadzadeh M, Talebi M, Eslamian F, Azari E. Effect of virtual reality-based balance training in multiple sclerosis. *Neurol Res* 2015; **37**: 539–44.
73 Bueno Espinosa I, Gonzalez Platas M, Reyes Chavez JJ, *et al.* Randomized and blinded study to assess the complementary therapies contribution to treat MS. In: MULTIPLE SCLEROSIS JOURNAL. SAGE PUBLICATIONS LTD 1 OLIVERS YARD, 55 CITY ROAD, LONDON EC1Y 1SP, ENGLAND, 2015: NP21-NP21.
74 Feys P, Coninx K, Kerkhofs L, *et al.* Robot-supported upper limb training in a virtual learning environment : a pilot randomized controlled trial in persons with MS. *J Neuroeng Rehabil* 2015; **12**: 60.
75 Feys P, Moumdjian L, Vanhalewyck F, *et al.* Effects of an individual 12 weeks community located running program on physical capacity, walking, cognitive function, dual tasking and brain volumes and structures in persons with Multiple Sclerosis. *Mult Scler* 2016; **22**: 73–4.
76 Fimland MS, Helgerud J, Gruber M, Leivseth G, Hoff J. Enhanced neural drive after maximal strength training in multiple sclerosis patients. *Eur J Appl Physiol* 2010; **110**: 435–43.
77 Fox EE, Hough AD, Creanor S, Gear M, Freeman JA. Effects of Pilates-Based Core Stability Training in Ambulant People With Multiple Sclerosis: Multicenter, Assessor-Blinded, Randomized Controlled Trial. *Phys Ther* 2016; **96**: 1170–8.
78 Frevel D, Maeurer M. Internet-based home training is capable to improve balance in multiple sclerosis: a randomized controlled trial. *Eur J Phys Rehabil Med* 2015; **51**: 23–30.
79 Gandolfi M, Geroin C, Picelli A, *et al.* Robot-assisted vs. sensory integration training in treating gait and balance dysfunctions in patients with multiple sclerosis: a randomized controlled trial. *Front Hum Neurosci* 2014; **8**: 318.
80 Gandolfi M, Munari D, Geroin C, *et al.* Sensory integration balance training in patients with multiple sclerosis: A randomized, controlled trial. *Mult Scler* 2015; **21**: 1453–62.
81 Garrett M, Hogan N, Larkin A, Saunders J, Jakeman P, Coote S. Exercise in the community for people with minimal gait impairment due to MS: an assessor-blind randomized controlled trial. *Mult Scler J* 2012; : 1352458512461966.
82 Garrett M, Hogan N, Larkin A, Saunders J, Jakeman P, Coote S. Exercise in the community for people with multiple sclerosis--a follow-up of people with minimal gait impairment. *Mult Scler* 2013; **19**: 790–8.
83 Gervasoni E, Cattaneo D, Jonsdottir J. Effect of treadmill training on fatigue in multiple sclerosis: a pilot study. *Int J Rehabil Res Int Zeitschrift für Rehabil Rev Int Rech réadaptation* 2014; **37**: 54–60.
84 Ghafari S, Ahmadi F, Nabavi M, Anoshirvan K, Memarian R, Rafatbakhsh M. Effectiveness of applying progressive muscle relaxation technique on quality of life of patients with multiple sclerosis. *J Clin Nurs* 2009; **18**: 2171–9.
85 Giovannelli M, Borriello G, Castri P, Prosperini L, Pozzilli C. Early physiotherapy after injection of botulinum toxin increases the beneficial effects on spasticity in patients with multiple sclerosis. *Clin Rehabil* 2007; **21**: 331–7.
86 Golzari Z, Shabkhiz F, Soudi S, Kordi MR, Hashemi SM. Combined exercise training reduces IFN-?? and IL-17 levels in the plasma and the supernatant of peripheral blood mononuclear cells in women with multiple sclerosis. *Int Immunopharmacol* 2010; **10**: 1415–9.
87 Hasanpour DA. Influence of yoga and aerobics exercise on fatigue, pain and psychosocial status in patients with multiple sclerosis: a randomized trial. *J Sports Med Phys Fitness* 2016; **56**: 1417–22.
88 Hayes H a, Gappmaier E, LaStayo PC. Effects of high-intensity resistance training on strength, mobility, balance, and fatigue in individuals with

multiple sclerosis: a randomized controlled trial. *J Neurol Phys Ther* 2011; **35**: 2–10.
89 Hebert JR, Corboy JR, Manago MM, Schenkman M. Effects of vestibular rehabilitation on multiple sclerosis-related fatigue and upright postural control: A randomized controlled trial. *137th Annu Meet Am Neurol Assoc Boston, MA United States* 2012; **72**: S34–5.
90 Heine M, Verschuren O, Hoogervorst E, *et al.* Does aerobic training alleviate fatigue and improve societal participation in patients with multiple sclerosis? The TREFAMS-A multicentre randomised trial. *J Mult Scler* 2017; **23**: 1517-26.
91 Hoang P, Schoene D, Gandevia S, Smith S, Lord SR. Effects of a home-based step training programme on balance, stepping, cognition and functional performance in people with multiple sclerosis - a randomized controlled trial. *Mult Scler* 2015; : 1–10.
92 Hogan N, Kehoe M, Larkin A, Coote S. The Effect of Community Exercise Interventions for People with MS Who Use Bilateral Support for Gait. *Mult Scler Int* 2014; **2014**: 109142.
93 Hojjatollah NB, Khosrow E, Reza RS, Monireh MN. Effects of selected combined training on muscle strength in multiple sclerosis patients. *HealthMED* 2012; **6**: 1258–64.
94 Jäckel N, Tallner A, Virsevci Ö, *et al.* Effects of internet-based exercise (e-training) on fatigue and other patient reported behavioural outcomes (PRO) in patients with relapsing-remitting multiple sclerosis (RRMS). *Mult Scler* 2015; **23**: 616.
95 Jonsdottir J, Gervasoni E, Lencioni T, Crippa A, Rovaris M, Cattaneo D. Positive effects of multi-modal intensive aerobic training on mobility and cognitive functions of persons with multiple sclerosis, with corresponding neuromodular reorganization of leg muscle synergies. *Mult Scler* 2014; **20**: 968.
96 Kalron A, Rosenblum U, Frid L, Achiron A. Pilates exercise training vs. physical therapy for improving walking and balance in people with multiple sclerosis: A randomized controlled trial. *Clin Rehabil* 2016; : 269215516637202.
97 Kargarfard M, Etemadifar M, Baker P, Mehrabi M, Hayatbakhsh R. Effect of aquatic exercise training on fatigue and health-related quality of life in patients with multiple sclerosis. *Arch Phys Med Rehabil* 2012; **93**: 1701–8.
98 Kargarfard M, Mehrabi M, Hamidi-Tehrani J, Rouzbahani R. Changes in speed, endurance and balance in women with multiple sclerosis after 4 and 8 weeks of aquatic exercise training. *J Isfahan Med Sch* 2013; **31**. http://www.embase.com/search/results?subaction=viewrecord&from=export&id=L373856345.
99 Kerling A, Keweloh K, Tegtbur U, *et al.* Effects of a short physical exercise intervention on patients with multiple sclerosis (MS). *Int J Mol Sci* 2015; **16**: 15761–75.
100 Khan F, Pallant JF, Brand C, Kilpatrick TJ. Effectiveness of rehabilitation intervention in persons with multiple sclerosis: a randomised controlled trial. *J Neurol Neurosurg Psychiatry* 2008; **79**: 1230–5.
101 Khurana SR, Beranger AG, Brooks L, Dominguez A, Moore JG. Effects of exercise and copaxone on six minute walk test in patients with multiple sclerosis: A pilot study. *PM R* 2012; **4**: S290.
102 Kjølhede T, Vissing K, de Place L, *et al.* Neuromuscular adaptations to long-term progressive resistance training translates to improved functional capacity for people with multiple sclerosis and is maintained at follow-up. *Mult Scler* 2014; : 1–13.
103 Kooshiar H, Moshtagh M, Sardar MA, Foroughipour M, Shakeri MT, Vahdatinia B. Fatigue and quality of life of women with multiple sclerosis: a randomized controlled clinical trial. *J Sports Med Phys Fitness* 2015; **55**: 668–74.
104 Küçük F, Kara B, Poyraz EÇ, İdiman E. Improvements in cognition, quality of life, and physical performance with clinical Pilates in multiple sclerosis: a randomized controlled trial. *J Phys Ther Sci* 2016; **28**: 761–8.
105 Learmonth YC, Paul L, Miller L, Mattison P, McFadyen AK. The effects of a 12-week leisure centre-based, group exercise intervention for people

moderately affected with multiple sclerosis: a randomized controlled pilot study. *Clin Rehabil* 2012; **26**: 579–93.
106 Leavitt VM, Cirnigliaro C, Cohen A, *et al.* Aerobic exercise increases hippocampal volume and improves memory in multiple sclerosis: Preliminary findings. *Neurocase* 2014; **20**: 695–7.
107 Leone C, Cimino V, Dibilio V, *et al.* Wii may improve physical abilities in multiple sclerosis: A randomized single blind study. *Mult Scler* 2014; **20**: 1005.
108 Louie J, Baquie KA, Offerman J, Bower KJ, Granger CL, Khan F. Maximising abilities, negotiating and generating exercise options (manage) program: A pilot randomised controlled trial in persons with multiple sclerosis. *Physiother (United Kingdom)* 2015; **101**: eS901-eS902.
109 Majdinasab N, Nakhostin-Mortazavi A, Bahadoram M, Pouretezad M, Afraz P. Effect of Aquatic Therapy on Functional Balance in Patients with Multiple Sclerosis: A Randomized Controlled Trial. *Persian J Med Sci* 2016; **3**.
110 McCullagh R, Fitzgerald AP, Murphy RP, Cooke G. Long-term benefits of exercising on quality of life and fatigue in multiple sclerosis patients with mild disability: a pilot study. *Clin Rehabil* 2008; **22**: 206–14.
111 Medina-Perez C, De Souza-Teixeira F, Fernandez-Gonzalo R, De Paz-Fernandez JA. Effects of a resistance training program and subsequent detraining on muscle strength and muscle power in multiple sclerosis patients. *NeuroRehabilitation* 2014; **34**: 523–30.
112 Miller L, Paul L, Mattison P, McFadyen a. Evaluation of a home-based physiotherapy programme for those with moderate to severe multiple sclerosis: a randomized controlled pilot study. *Clin Rehabil* 2011; **25**: 720–30.
113 Moghadasi M, Edalatmanesh MA, Moeini A, Nematollahzadeh Mahani MS. Effects of eight weeks resistance training on brain derived neurotrophic factor in female patients with multiple sclerosis. *Koomesh* 2015; **17**: 152–9.
114 Moradi M, Sahraian MA, Aghsaie A, *et al.* Effects of eight-week resistance training program in men with multiple sclerosis. *Asian J Sports Med* 2015; **6**.
115 Mori F, Ljoka C, Magni E, *et al.* Transcranial magnetic stimulation primes the effects of exercise therapy in multiple sclerosis. *J Neurol* 2011; **258**: 1281–7.
116 Negahban H, Rezaie S, Goharpey S. Massage therapy and exercise therapy in patients with multiple sclerosis: a randomized controlled pilot study. *Clin Rehabil* 2013; **27**: 1126–36.
117 Nilsagård YE, Forsberg AS, von Koch L. Balance exercise for persons with multiple sclerosis using Wii games: a randomised, controlled multi-centre study. *Mult Scler* 2013; **19**: 209–16.
118 O'Donnell M, Coote S. Physiotherapy intervention in persons with MS who are non-ambulatory. *Physiother (United Kingdom)* 2011; **97**: eS917.
119 Oken BS, Kishiyama S, Zajdel D, *et al.* Randomized controlled trial of yoga and exercise in multiple sclerosis. *Neurology* 2004; **62**: 2058–64.
120 Ozgen G. Is customized vestibular rehabilitation effective in patients with multiple sclerosis? A randomized controlled trial. *Eur J Phys Med Rehab* 2016; : 1–29.
121 Patti F, Ciancio MR, Reggio E, *et al.* The impact of outpatient rehabilitation on quality of life in multiple sclerosis. *J Neurol* 2002; **249**: 1027–33.
122 Patti F, Ciancio MR, Cacopardo M, *et al.* Effects of a short outpatient rehabilitation treatment on disability of multiple sclerosis patients - A randomised controlled trial. *J Neurol* 2003; **250**: 861–6.
123 Paul L, Coulter EH, Miller L, McFadyen A, Dorfman J, Mattison PGG. Web-based physiotherapy for people moderately affected with Multiple Sclerosis; quantitative and qualitative data from a randomized, controlled pilot study. *Clin Rehabil* 2014; **28**: 924–35.
124 Petajan JH, Gappmaier E, White a T, Spencer MK, Mino L, Hicks RW. Impact of aerobic training on fitness and quality of life in multiple sclerosis. *Ann Neurol* 1996; **39**: 432–41.
125 Plow M, Bethoux F, Mai K, Marcus B. A formative evaluation of customized pamphlets to promote physical activity and symptom self-management in

women with multiple sclerosis. *Health Educ Res* 2014; **29**: 883–96.
126 Prosperini L, Fortuna D, Gianni C, Leonardi L, Marchetti MR, Pozzilli C. Home-Based Balance Training Using the Wii Balance Board: A Randomized, Crossover Pilot Study in Multiple Sclerosis. *Neurorehabil Neural Repair* 2013; **27**: 516–25.
127 Rahnama N, Namazizadeh M, Etemadifar M, Bambaeichi E, Arbabzadeh S, Sadeghipour HR. Effects of yoga on depression in women with multiple sclerosis. *J Isfahan Med Sch* 2011; **29**. http://www.embase.com/search/results?subaction=viewrecord&from=export&id=L362030815.
128 Rampello A, Franceschini M, Piepoli M, Antenucci R. Effect of aerobic training on walking capacity and maximal exercise tolerance in patients with multiple sclerosis: a randomized crossover controlled study. *Phys Ther* 2007; **87**: 545.
129 Razazian N, Yavari Z, Farnia V, *et al.* Exercising impacts on fatigue, depression, and paresthesia in female patients with multiple sclerosis. *Med Sci Sports Exerc* 2016; **48**: 796–803.
130 Rietberg MB, Van Wegen EEH, Eyssen ICJM, Kwakkel G. Effects of multidisciplinary rehabilitation on chronic fatigue in multiple sclerosis: A randomized controlled trial. *PLoS One* 2014; **9**. DOI:10.1371/journal.pone.0107710.
131 Robinson J, Dixon J, Macsween A, van Schaik P, Martin D. The effects of exergaming on balance, gait, technology acceptance and flow experience in people with multiple sclerosis: a randomized controlled trial. *BMC Sports Sci Med Rehabil* 2015; **7**: 8.
132 Romberg A. Effects of a 6-month exercise program on patients with multiple sclerosis : A randomized study Effects of a 6-month exercise program on patients with multiple sclerosis A randomized study. 2005. DOI:10.1212/01.WNL.0000145761.38400.65.
133 Romberg A, Virtanen A, Ruutiainen J. Long-term exercise improves functional impairment but not quality of life in multiple sclerosis. *J Neurol* 2005; **252**: 839–45.
134 Ruiz J, Labas MP, Triche EW, Lo AC. Combination of Robot-Assisted and Conventional Body-Weight–Supported Treadmill Training Improves Gait in Persons With Multiple Sclerosis. *J Neurol Phys Ther* 2013; **37**: 187–93.
135 Sabapathy NM, Minahan CL, Turner GT, Broadley SA. Comparing endurance- and resistance-exercise training in people with multiple sclerosis: a randomized pilot study. *Clin Rehabil* 2011; **25**: 14–24.
136 Salhofer-Polanyi S, Windt J, Sumper H, *et al.* Benefits of inpatient multidisciplinary rehabilitation in multiple sclerosis. *NeuroRehabilitation* 2013; **33**: 285–92.
137 Sandroff BM, Balto JM, Klaren RE, Sommer SK, DeLuca J, Motl RW. Systematically developed pilot randomized controlled trial of exercise and cognition in persons with multiple sclerosis. *Neurocase* 2016; **0**: 1–8.
138 Sangelaji B, Nabavi SM, Estebsari F, *et al.* Effect of combination exercise therapy on walking distance, postural balance, fatigue and quality of life in multiple sclerosis patients: a clinical trial study. *Iran Red Crescent Med J* 2014; **16**: e17173.
139 Solari A, Filippini G, Gasco P, *et al.* Physical rehabilitation has a positive effect on disability in multiple sclerosis patients. *Neurology* 1999; **52**: 57–62.
140 Sosnoff JJ, Finlayson M, McAuley E, Morrison S, Motl RW. Home-based exercise program and fall-risk reduction in older adults with multiple sclerosis: phase 1 randomized controlled trial. *Clin Rehabil* 2014; **28**: 254–63.
141 Sosnoff JJ, Moon Y, Wajda D a, *et al.* Fall risk and incidence reduction in high risk individuals with multiple sclerosis: a pilot randomized control trial. *Clin Rehabil* 2015; **29**: 952–60.
142 Storr LK, Sorensen PS, Ravnborg M. The efficacy of multidisciplinary rehabilitation in stable multiple sclerosis patients. *Mult Scler* 2006; **12**: 235–42.
143 Straudi S, Martinuzzi C, Pavarelli C, *et al.* A task-oriented circuit training in multiple sclerosis: a feasibility study. *BMC Neurol* 2014; **14**: 124.
144 Straudi S, Fanciullacci C, Martinuzzi C, *et al.* The effects of robot-assisted gait training in progressive multiple sclerosis: A randomized controlled trial. *Mult Scler* 2016; **22**: 373–84.

145 Surakka J, Romberg A, Ruutiainen J, *et al.* Effects of aerobic and strength exercise on motor fatigue in men and women with multiple sclerosis: a randomized controlled trial. *Clin Rehabil* 2004; **18**: 737–46.
146 Sutherland G, Andersen MB, Stoové MA. Can aerobic exercise training affect health-related quality of life for people with multiple sclerosis? *J Sport Exerc Psychol* 2001; **23**: 122–35.
147 Tallner A, Maeurer M, Pfeifer K. An internet-based at home training protocol enhances muscle strength and lung function in multiple sclerosis patients (#133). *Mult Scler* 2012; **18**: S23–4.
148 Tarakci E, Yeldan I, Huseyinsinoglu BE, Zenginler Y, Eraksoy M. Group exercise training for balance, functional status, spasticity, fatigue and quality of life in multiple sclerosis: a randomized controlled trial. *Clin Rehabil* 2013; **27**: 813–22.
149 van den Berg M, Dawes H, Wade DT, *et al.* Treadmill training for individuals with multiple sclerosis: a pilot randomised trial. *J Neurol Neurosurg Psychiatry* 2006; **77**: 531–3.
150 van Kessel K, Moss-Morris R, Willoughby E, Chalder T, Johnson MH, Robinson E. A randomized controlled trial of cognitive behavior therapy for multiple sclerosis fatigue. *Psychosom Med* 2008; **70**: 205–13.
151 Velikonja O, ??uri?? K, O??ura A, Jazbec S ??ega. Influence of sports climbing and yoga on spasticity, cognitive function, mood and fatigue in patients with multiple sclerosis. *Clin Neurol Neurosurg* 2010; **112**: 597–601.
152 Wens I, Dalgas U, Vandenabeele F, *et al.* High intensity exercise in multiple sclerosis: Effects on muscle contractile characteristics and exercise capacity, a randomised controlled trial. *PLoS One* 2015; **10**: 1–14.
153 Wens I, Hansen D, Verboven K, *et al.* Impact of 24 Weeks of Resistance and Endurance Exercise on Glucose Tolerance in Persons with Multiple Sclerosis. *Am J Phys Med Rehabil* 2015; **94**: 838–47.
154 Wens I, Keytsman C, Deckx N, Cools N, Dalgas U, Eijnde BO. Brain derived neurotrophic factor in multiple sclerosis: Effect of 24 weeks endurance and resistance training. *Eur J Neurol* 2016; **23**: 1028–35.
155 Wens I, Dalgas U, Vandenabeele F, *et al.* High Intensity Aerobic and Resistance Exercise Can Improve Glucose Tolerance in Persons With Multiple Sclerosis. *Am J Phys Med Rehabil* 2016; : 1.
156 Wiles CM, Newcombe RG, Fuller KJ, *et al.* Controlled randomised crossover trial of the effects of physiotherapy on mobility in chronic multiple sclerosis. *J Neurol Neurosurg Psychiatry* 2001; **70**: 174–9.
157 Zenginler Y, Tarakci E, Kurtuncu M, Razak Ozdincler A. The impact of Nintendo Wii fit games on the balance and functionality of multiple sclerosis patients: A randomized controlled study. *Mult Scler* 2016; **22**: 826.
158 Allen NE, Canning CG, Sherrington C, *et al.* The effects of an exercise program on fall risk factors in people with Parkinson's disease: A randomized controlled trial. *Mov Disord* 2010; **25**: 1217–25.
159 Ashburn A, Fazakarley L, Ballinger C, Pickering R, McLellan LD, Fitton C. A randomised controlled trial of a home based exercise programme to reduce the risk of falling among people with Parkinson's disease. *J Neurol Neurosurg Psychiatry* 2007; **78**: 678–84.
160 Bega D, Corcos D, Stein J, *et al.* Yoga versus resistance training in Parkinson's disease: A 12-week pilot feasibility study. *Mov Disord* 2015; **30**: S69–70.
161 Belton A. The effect of a balance exercise class on activity limitations in people with parkinson's disease. 2014.
162 Burini D, Farabollini B, Iacucci S, *et al.* A randomised controlled cross-over trial of aerobic training versus Qigong in advanced Parkinson's disease. *Eura Medicophys* 2006; **42**: 231–8.
163 Cakit BD, Saracoglu M, Genc H, Erdem HR, Inan L. The effects of incremental speed-dependent treadmill training on postural instability and fear of

falling in Parkinson's disease. *Clin Rehabil* 2007; **21**: 698–705.
164 Canning CG, Allen NE, Dean CM, Goh L, Fung VS. Home-based treadmill training for individuals with Parkinson's disease: a randomized controlled pilot trial. *Clin Rehabil* 2012; **26**: 817–26.
165 Canning CG, Sherrington C, Lord SR, *et al.* Exercise for falls prevention in Parkinson disease A randomized controlled trial. *Am Acad Neurol* 2014; **84**: 304–12.
166 Capecci M, Serpicelli C, Fiorentini L, *et al.* Postural rehabilitation and kinesio taping for axial postural disorders in Parkinson's disease. *Arch Phys Med Rehabil* 2014; **95**: 1067–75.
167 Carvalho A, Dannyel B, Araujo N, *et al.* Comparison of strength training , aerobic training , and additional physical therapy as supplementary treatments for Parkinson ' s disease : pilot study. *Clin Interv Aging* 2015; **10**: 183–91.
168 Catalan M, Mezzarobba S, Pellegrini L, *et al.* Neurocognitive rehabilitation by improving motor planning versus treadmill training for freezing of gait in Parkinson's disease: A clinical and transcranial magnetic stimulation (TMS) study. *J Neurol* 2013; **260**: S98.
169 Choi H-J, Garber CE, Jun T-W, Jin Y-S, Chung S-J, Kang H-J. Therapeutic effects of Tai Chi in patients with Parkinson's disease. *ISRN Neurol* 2013; **1**. DOI:10.1155/2013/548240.
170 Cholewa J, Boczarska-Jedynak M, Opala G. Wp??yw systematycznie prowadzonej fizjoterapii na nasilenie objaw??w ruchowych oraz jako???? ??ycia u os??b z choroba{ogonek} Parkinsona. *Neurol Neurochir Pol* 2013; **47**: 256–62.
171 Clarke CE, Patel S, Ives N, *et al.* Physiotherapy and Occupational Therapy vs No Therapy in Mild to Moderate Parkinson Disease: A Randomized Clinical Trial. *JAMA Neurol* 2016; **73**: 291–9.
172 Yvonne Searls Colgrove*, Neena Sharma, Patricia Kluding, Debra Potter, Kayce Imming, Jessica VandeHoef, Jill Stanhope K, Hoffman and KW. Effect of Yoga on Motor Function in People with Parkinson's Disease: A Randomized, Controlled Pilot Study. *J Yoga Phys Ther* 2012; **2**: pp 1-11.
173 Combs SA, Diehl MD, Chrzastowski C, *et al.* Community-based group exercise for persons with Parkinson disease: A randomized controlled trial. *NeuroRehabilitation* 2013; **32**: 117–24.
174 Comelia CL, Stebbins GT, Brown-Toms N, Goetz CG. Physical therapy and Parkinson's disease A controlled clinical trial. *Neurology* 1994; **44**: 376.
175 Conradsson D, Löfgren N, Nero H, *et al.* The Effects of Highly Challenging Balance Training in Elderly With Parkinson's Disease: A Randomized Controlled Trial. *Neurorehabil Neural Repair* 2015; **29**: 827–36.
176 Corcos DM, Robichaud JA, David FJ, *et al.* A two-year randomized controlled trial of progressive resistance exercise for Parkinson's disease. *Mov Disord* 2013; **28**: 1230–40.
177 Cugusi L, Solla P, Serpe R, *et al.* Effects of a Nordic Walking program on motor and non-motor symptoms, functional performance and body composition in patients with Parkinson's disease. *NeuroRehabilitation* 2015; **37**: 245–54.
178 Dashtipour K, Johnson E, Kani C, *et al.* Effect of exercise on motor and nonmotor symptoms of Parkinson's disease. *Parkinsons Dis* 2015; **2015**. DOI:10.1155/2015/586378.
179 David FJ, Robichaud JA, Leurgans SE, *et al.* Exercise improves cognition in Parkinson's disease: The PRET-PD randomized, clinical trial. *Mov Disord* 2015; **30**: 1657–63.
180 de Bruin N, Doan JB, Turnbull G, *et al.* Walking with music is a safe and viable tool for gait training in Parkinson's disease: the effect of a 13-week feasibility study on single and dual task walking. *Parkinsons Dis* 2010; **2010**: 483530.
181 de Oliveira RT, Felippe LA, Bucken Gobbi LT, Barbieri FA, Christofoletti G. Benefits of Exercise on the Executive Functions in People with Parkinson Disease. *Am J Phys Med Rehabil* 2016; **0**: 1.

182 Di Biagio L, Andrenelli E, Sordoni E, Monsù AM, Ceravolo MG, Capecci M. Gait and balance training in advanced Parkinson's disease: Comparative study of three methods. *Ann Phys Rehabil Med* 2014; **57**: e362.
183 DiFrancisco-Donoghue J, Lamberg EM, Rabin E, Elokda A, Fazzini E, Werner WG. Effects of exercise and B vitamins on homocysteine and glutathione in Parkinson's disease: A randomized trial. *Neurodegener Dis* 2012; **10**: 127–34.
184 Duncan RP, Earhart GM. Randomized Controlled Trial of Community-Based Dancing to Modify Disease Progression in Parkinson Disease. *Neurorehabil Neural Repair* 2012; **26**: 132–43.
185 Duncan RP, Earhart GM. Are the Effects of Community-Based Dance on Parkinson Disease Severity, Balance, and functional mobility reduced with time? A 2-year prospective pilot study. *J Altern Complement Med* 2014; **20**: 757–63.
186 Ebersbach G, Edler D, Kaufhold O, Wissel J. Whole Body Vibration Versus Conventional Physiotherapy to Improve Balance and Gait in Parkinson's Disease. *Arch Phys Med Rehabil* 2008; **89**: 399–403.
187 Ebersbach G, Ebersbach A, Edler D, *et al.* Comparing exercise in Parkinson's disease - The Berlin LSVT®BIG study. *Mov Disord* 2010; **25**: 1902–8.
188 Ellis T, De Goede CJ, Feldman RG, Wolters EC, Kwakkel G, Wagenaar RC. Efficacy of a physical therapy program in patients with Parkinson's disease: A randomized controlled trial. *Arch Phys Med Rehabil* 2005; **86**: 626–32.
189 Fisher BE, Wu AD, Salem GJ, *et al.* The Effect of Exercise Training in Improving Motor Performance and Corticomotor Excitability in People With Early Parkinson's Disease. *Arch Phys Med Rehabil* 2008; **89**: 1221–9.
190 Fitton C, Kunkel D, Hulbert S, *et al.* Dancing with parkinson's disease: Feasibility randomised controlled trial. *Physiother (United Kingdom)* 2015; **101**: eS384-eS385.
191 Foster ER, Golden L, Duncan RP, Earhart GM. Community-based argentine tango dance program is associated with increased activity participation among individuals with parkinson's disease. *Arch Phys Med Rehabil* 2013; **94**: 240–9.
192 Frazzitta G, Bertotti G, Riboldazzi G, *et al.* Effectiveness of intensive inpatient rehabilitation treatment on disease progression in parkinsonian patients: a randomized controlled trial with 1-year follow-up. *Neurorehabil Neural Repair* 2012; **26**: 144–50.
193 Frazzitta G, Maestri R, Ghilardi MF, *et al.* Intensive Rehabilitation Increases BDNF Serum Levels in Parkinsonian Patients: A Randomized Study. *Neurorehabil Neural Repair* 2014; **28**: 163–8.
194 Frazzitta G, Maestri R, Bertotti G, *et al.* Intensive Rehabilitation Treatment in Early Parkinson's Disease A Randomized Pilot Study With a 2-Year Follow-up. *Neurorehabil Neural Repair* 2015; **29**: 123–31.
195 Ganesan M, Sathyaprabha TN. Effect of Partial WeighteSupported Treadmill Gait Training on Balance in Patients With Parkinson Disease. *Pm R* 2013; **6**: 1–12.
196 Ganesan M, Sathyaprabha TN, Pal PK, Gupta A. Partial Body Weight-Supported Treadmill Training in Patients with Parkinson Disease: Impact on Gait and Clinical Manifestation. *Arch Phys Med Rehabil* 2015; **96**: 1557–65.
197 Gao Q, Leung A, Yang Y, *et al.* Effects of Tai Chi on balance and fall prevention in Parkinson's disease: a randomized controlled trial. *Clin Rehabil* 2014; **28**: 748–53.
198 Gobbi LT, Teixeira-Arroyo C, Lirani-Silva E, Vitório R, Barbieri FA, Pereira MP. Effect of different exercise programs on the psychological and cognitive functions of people with Parkinson's disease. 2013; **19**: 597–604.
199 Goodwin VA, Richards SH, Henley W, Ewings P, Taylor AH, Campbell JL. An exercise intervention to prevent falls in people with Parkinson's disease: a pragmatic randomised controlled trial. *J Neurol Neurosurg Psychiatry* 2011; **82**: 1232–8.
200 Gu S, Song Z, Fan X, Chen R, Zheng W, Yan W. Effect of PD-WEBB training on balance impairment and falls in people with Parkinson's disease.

Zhong Nan Da Xue Xue Bao Yi Xue Ban 2013; **38**: 1172–6.
201 Hackney ME, Earhart GM. Tai Chi improves balance and mobility in people with Parkinson disease. *Gait Posture* 2008; **28**: 456–60.
202 Hackney ME, Earhart GM. Effects of dance on movement control in Parkinson's disease: A comparison of Argentine tango and American ballroom. *J Rehabil Med* 2009; **41**: 475–81.
203 Harro CC, Shoemaker MJ, Frey O, *et al.* The effects of speed-dependent treadmill training and rhythmic auditory-cued overground walking on balance function, fall incidence, and quality of life in individuals with idiopathic Parkinson's disease: A randomized controlled trial. *NeuroRehabilitation* 2014; **34**: 541–56.
204 Harro CC, Shoemaker MJ, Frey OJ, *et al.* The effects of speed-dependent treadmill training and rhythmic auditory-cued overground walking on gait function and fall risk in individuals with idiopathic Parkinson's disease: A randomized controlled trial. *NeuroRehabilitation* 2014; **34**: 557–72.
205 Hass CJ, Buckley TA, Pitsikoulis C, Barthelemy EJ. Progressive resistance training improves gait initiation in individuals with Parkinson's disease. *Gait Posture* 2012; **35**: 669–73.
206 Keus SHJ, Bloem BR, van Hilten JJ, Ashburn A, Munneke M. Effectiveness of physiotherapy in Parkinson's disease: The feasibility of a randomised controlled trial. *Park Relat Disord* 2007; **13**: 115–21.
207 Kurtais Y, Kutlay S, Tur BS, Gok H. Does Treadmill Training Improve Lower-Extremity Tasks in Parkinson Disease ? A Randomized Controlled Trial. 2008; **18**: 289–91.
208 Laupheimer M, Härtel S, Schmidt S, Bös K. Exercise training - Effects of MOTOmed® exercise on typical motor dysfunction in Parkinson s disease. *Neurol und Rehabil* 2011; **17**: 239–46.
209 Lee N-Y, Lee D-K, Song H-S. Effect of virtual reality dance exercise on the balance, activities of daily living, and depressive disorder status of Parkinson's disease patients. *J Phys Ther Sci* 2015; **27**: 145–7.
210 Fuzhong L, Harmer P, FitzGerald K, *et al.* Tai Chi and Postural Stability in Patients with Parkinson's Disease. *N Engl J Med* 2012; : 511–9.
211 Li F, Harmer P, Liu Y, *et al.* A randomized controlled trial of patient-reported outcomes with tai chi exercise in Parkinson's disease. *Mov Disord* 2014; **29**: 539–45.
212 Liao Y-Y, Yang Y-R, Cheng S-J, Wu Y-R, Fuh J-L, Wang R-Y. Virtual Reality-Based Training to Improve Obstacle-Crossing Performance and Dynamic Balance in Patients With Parkinson's Disease. *Neurorehabil Neural Repair* 2015; **29**: 658–67.
213 Liao Y-Y, Yang Y-R, Wu Y-R, Wang R-Y. Virtual Reality-Based Wii Fit Training in Improving Muscle Strength, Sensory Integration Ability, and Walking Abilities in Patients with Parkinson's Disease: A Randomized Control Trial. *Int J Gerontol* 2015; **9**: 190–5.
214 Mak MKY, Hui-Chan CWY. Cued task-specific training is better than exercise in improving sit-to-stand in patients with Parkinson's disease: A randomized controlled trial. *Mov Disord* 2008; **23**: 501–9.
215 McCamish J, Samson A, Vrongistinos K, Jung T. The effects of cardiovascular exercise on cognitive function in individuals with Parkinson's disease (PD). *Mov Disord* 2013; **28**: S200–1.
216 Mezzarobba S, Pellegrini L, Giulia S, *et al.* Neurocognitive rehabilitation with motor imagery vs treadmill training for freezing of gait in Parkinson's disease: A randomized controlled study. *J Parkinsons Dis* 2013; **3**: 152.
217 Mirabella G, De Vita P, Rampelli S, *et al.* Theatre is a valid complementary therapeutic intervention for emotional rehabilitation in parkinson's disease patients. *Park Relat Disord* 2016; **22**: e74–5.
218 Miyai I, Fujimoto Y, Yamamoto H, *et al.* Long-term effect of body weight-supported treadmill training in Parkinson's disease: A randomized controlled trial. *Arch Phys Med Rehabil* 2002; **83**: 1370–3.

219 Monticone M, Ambrosini E, Laurini A, Rocca B, Foti C. In-patient multidisciplinary rehabilitation for Parkinson's disease: A randomized controlled trial. *Mov Disord* 2015; **30**: 1050–8.
220 Morris ME, Iansek R, Kirkwood B. A randomized controlled trial of movement strategies compared with exercise for people with Parkinson's disease. *Mov Disord* 2009; **24**: 64–71.
221 Morris ME, Menz HB, McGinley JL, *et al.* A Randomized Controlled Trial to Reduce Falls in People With Parkinson's Disease. *Neurorehabil Neural Repair* 2015; **29**: 777–85.
222 Morrone M, Miccinilli S, Bravi M, *et al.* Perceptive rehabilitation and trunk posture alignment in patients with Parkinson disease: a single blind randomized controlled trial. *Eur J Phys Rehabil Med* 2016; **52**: 799–809.
223 Nadeau A, Pourcher E, Corbeil P. Effects of 24 wk of treadmill training on gait performance in parkinson's disease. *Med Sci Sports Exerc* 2014; **46**: 645–55.
224 Ni M, Mooney K, Signorile JF. Controlled pilot study of the effects of power yoga in Parkinson's disease. *Complement Ther Med* 2016; **25**: 126–31.
225 Ni M, Signorile JF, Balachandran A, Potiaumpai M. Power training induced change in bradykinesia and muscle power in Parkinson's disease. *Park Relat Disord* 2016; **23**: 37–44.
226 Ni M, Signorile JF, Mooney K, *et al.* Comparative effect of power training and high-speed yoga on motor function in older patients with parkinson disease. *Arch Phys Med Rehabil* 2016; **97**: 345–354.e15.
227 Park A, Zid D, Russell J, *et al.* Effects of a formal exercise program on Parkinson's disease: A pilot study using a delayed start design. *Park Relat Disord* 2014; **20**: 106–11.
228 Park Y. Effects of communal exercise with visual and auditory feedback provided by a smart application on gait ability and fear of falling in Parkinson ' s disease patients. 2014; **10**: 286–90.
229 Picelli A, Melotti C, Origano F, Waldner A, Gimigliano R, Smania N. Parkinsonism and Related Disorders Does robotic gait training improve balance in Parkinson ' s disease ? A randomized controlled trial. *Park Relat Disord* 2012; **18**: 990–3.
230 Picelli A, Melotti C, Origano F, *et al.* Robot-assisted gait training is not superior to balance training for improving postural instability in patients with mild to moderate Parkinson ' s disease : a single-blind randomized controlled trial. 2015. DOI:10.1177/0269215514544041.
231 Picelli A, Varalta V, Melotti C, *et al.* Effects of treadmill training on cognitive and motor features of patients with mild to moderate Parkinson's disease: A pilot, single-blind, randomized controlled trial. *Funct Neurol* 2016; **31**: 25–31.
232 Pikel MR, Costa ALD, Nogueira LM, Okamoto E, Piemonte MEP. Mental practice may improve the gait stability in patients with Parkinson's disease: a single-blind, randomised clinical trial. *Physiotherapy* 2015; **101**: e1208–9.
233 Poliakoff E, Galpin AJ, Mcdonald K, *et al.* The effect of gym training on multiple outcomes in Parkinson's disease: A pilot randomised waiting-list controlled trial. *NeuroRehabilitation* 2013; **32**: 125–34.
234 Prodoehl J, Rafferty MR, David FJ, *et al.* Two-Year Exercise Program Improves Physical Function in Parkinson ' s Disease : The PRET-PD Randomized Clinical Trial. 2016. DOI:10.1177/1545968314539732.
235 Protas EJ, Mitchell K, Williams A, *et al.* Gait and step training to reduce falls in Parkinson ' s disease. 2005; **20**: 183–90.
236 Qutubuddin A, Reis T, Alramadhani R, Cifu DX, Towne A, Carne W. Parkinson's disease and forced exercise: a preliminary study. *Rehabil Res Pract* 2013; **2013**: 375267.
237 Reuter I, Mehnert S, Leone P, Kaps M, Oechsner M, Engelhardt M. Effects of a Flexibility and Relaxation Programme , Walking , and Nordic Walking on Parkinson ' s Disease. 2011; **2011**. DOI:10.4061/2011/232473.

238 Reuter I, Mehnert S, Sammer G, Oechsner M, Engelhardt M. Efficacy of a Multimodal Cognitive Rehabilitation Including Psychomotor and Endurance Training in Parkinson ' s Disease. 2012; **2012**. DOI:10.1155/2012/235765.
239 Ridgel AL, Fickes-Ryan EJ, Wilson KA. Effects of active-assisted cycling on motor function and balance in Parkinson's disease. *J Neurol Sci* 2013; **333**: e91.
240 Romenets SR, Anang J, Fereshtehnejad S-M, Pelletier A, Postuma R. Tango for treatment of motor and non-motor manifestations in Parkinson's disease: a randomized control study. *Complement Ther Med* 2015; **23**: 175–84.
241 Sacheli MA, Almeida QJ. The addition of aerobic or resistance training to sensory attention focused exercise: An enhanced treatment for Parkinson's disease? *Mov Disord* 2012; **27**: S136–7.
242 Sage MD, Almeida QJ. Symptom and Gait Changes After Sensory Attention Focused Exercise vs Aerobic Training in Parkinson ' s Disease. 2009; **24**: 1132–8.
243 Schenkman M, Cutson TM, Kuchibhatla M, *et al.* Exercise to improve spinal flexibility and function for people with Parkinson's disease: A randomized, controlled trial. *J Am Geriatr Soc* 1998; **46**: 1207–16.
244 Schenkman M, Hall DA, Baron AE, *et al.* Exercise for people in early- or mid-stage Parkinson disease: a 16-month randomized controlled trial. *Phys Ther* 2012; **92**: 1395–410.
245 Schilling BK, Pfeiffer RF, Ledoux MS, Karlage RE, Bloomer RJ, Falvo MJ. Effects of moderate-volume, high-load lower-body resistance training on strength and function in persons with Parkinson's disease: a pilot study. *Parkinsons Dis* 2010; **2010**: 824734.
246 Schlenstedt C, Paschen S, Kruse A, Raethjen J, Weisser B, Deuschl G. Resistance versus balance training to improve postural control in Parkinson's disease: A randomized rater blinded controlled study. *PLoS One* 2015; **10**: 1–18.
247 Schmitz-Hübsch T, Pyfer D, Kielwein K, Fimmers R, Klockgether T, Wüllner U. Qigong exercise for the symptoms of Parkinson's disease: a randomized, controlled pilot study. *Mov Disord* 2006; **21**: 543–8.
248 Sedaghati P, Daneshmandi H, Karimi N, Barati AH. A selective corrective exercise to decrease falling and improve functional balance in idiopathic Parkinson's Disease. *Trauma Mon* 2016; **21**: 1–6.
249 Sharma NK, Robbins K, Wagner K, Colgrove YM. A randomized controlled pilot study of the therapeutic effects of yoga in people with Parkinson's disease. *Int J Yoga* 2015; **8**: 74.
250 Shen X, Mak MKY. Repetitive step training with preparatory signals improves stability limits in patients with Parkinson's disease. *J Rehabil Med* 2012; **44**: 944–9.
251 Shen X, Mak MKY. Balance and Gait Training With Augmented Feedback Improves Balance Confidence in People With Parkinson's Disease: A Randomized Controlled Trial. *Neurorehabil Neural Repair* 2014; **28**: 524–35.
252 Shen X, Mak MKY. Technology-assisted balance and gait training reduces falls in patients with Parkinson's disease: a randomized controlled trial with 12-month follow-up. *Neurorehabil Neural Repair* 2015; **29**: 103–11.
253 Shulman LM, Katzel LI, Ivey FM, *et al.* Randomized clinical trial of 3 types of physical exercise for patients with Parkinson disease. *JAMA Neurol* 2013; **70**: 183–90.
254 Silva-Batista C, Corcos DM, Roschel H, *et al.* Resistance Training with Instability for Patients with Parkinson's Disease. *Med Sci Sports Exerc* 2016; **48**: 1678–87.
255 Smania N, Corato E, Tinazzi M, *et al.* Effect of balance training on postural instability in patients with idiopathic Parkinson's disease. *Neurorehabil Neural Repair* 2010; **24**: 826–34.

256 Sparrow D, DeAngelis TR, Hendron K, Thomas C a, Saint-Hilaire M, Ellis T. Highly Challenging Balance Program Reduces Fall Rate in Parkinson Disease. *J Neurol Phys Ther* 2016; **40**: 24–30.
257 Stack E, Roberts H, Ashburn A. The PIT: SToPP trial- A feasibility randomised controlled trial of home-based physiotherapy for people with Parkinson's disease using video-based measures to preserve assessor blinding. *Parkinsons Dis* 2012; **2012**. DOI:10.1155/2012/360231.
258 Stożek J, Rudzińska M, Pustułka-Piwnik U, Szczudlik A. The effect of the rehabilitation program on balance, gait, physical performance and trunk rotation in Parkinson's disease. *Aging Clin Exp Res* 2015; : 1–9.
259 Taheri H, Pejhan A, Taherzadeh J, Seyedahmadi M, Keavanloo F. Effect of a physical therapy program based on balance and gait in patients with Parkinson. *J Isfahan Med Sch* 2011; **29**: 1183–91.
260 Tickle-Degnen L, Ellis T, Saint-Hilaire MH, Thomas CA, Wagenaar RC. Self-management rehabilitation and health-related quality of life in Parkinson's disease: A randomized controlled trial. *Mov Disord* 2010; **25**: 194–204.
261 Peppe A, Bonníbio S, Giacomo K, Martino Cinnera A, Tramontano M, Caltagirone C. Blindfolded balance training in patients with Parkinson's disease: A sensory-motor strategy to improve the gait. *Mov Disord* 2016; **31**: S674.
262 Wade DT, Gage H, Owen C, Trend P, Grossmith C, Kaye J. Multidisciplinary rehabilitation for people with Parkinson's disease: a randomised controlled study. *J Neurol Neurosurg Psychiatry* 2003; **74**: 158–62.
263 Wong-Yu IS, Mak MK. Task- and Context-Specific Balance Training Program Enhances Dynamic Balance and Functional Performance in Parkinsonian Nonfallers: A Randomized Controlled Trial with Six-Month Follow-Up. *Arch Phys Med Rehabil* 2015; **96**: 2103–11.
264 Wong-Yu ISK, Mak MKY. Multi-dimensional balance training programme improves balance and gait performance in people with Parkinson's disease: A pragmatic randomized controlled trial with 12-month follow-up. *Parkinsonism Relat Disord* 2015; **21**: 615–21.
265 Xiao C-M, Zhuang Y-C. Effect of health Baduanjin Qigong for mild to moderate Parkinson's disease. *Geriatr Gerontol Int* 2016; **16**: 911–9.
266 Yang Y-R, Lee Y-Y, Cheng S-J, Wang R-Y. Downhill walking training in individuals with Parkinson's disease: a randomized controlled trial. *Am J Phys Med Rehabil* 2010; **89**: 706–14.
267 Yen C-Y, Lin K-H, Hu M-H, Wu R-M, Lu T-W, Lin C-H. Effects of virtual reality-augmented balance training on sensory organization and attentional demand for postural control in people with Parkinson disease: a randomized controlled trial. *Phys Ther* 2011; **91**: 862.
268 Zhang T-Y, Hu Y, Nie Z-Y, *et al.* Effects of Tai Chi and Multimodal Exercise Training on Movement and Balance Function in Mild to Moderate Idiopathic Parkinson Disease. *Am J Phys Med Rehabil* 2015; **94**: 921–9.
269 Battaglia G, Alesi M, Inguglia M, *et al.* Soccer practice as an add-on treatment in the management of individuals with a diagnosis of schizophrenia. *Neuropsychiatr Dis Treat* 2013; **9**: 595–603.
270 Beebe LH, Tian L, Morris N, Goodwin A, Allen SS, Kuldau J. Effects of exercise on mental and physical health parameters of persons with schizophrenia. *Issues Ment Health Nurs* 2005; **26**: 661–76.
271 Behere R V., Arasappa R, Jagannathan a., *et al.* Effect of yoga therapy on facial emotion recognition deficits, symptoms and functioning in patients with schizophrenia. *Acta Psychiatr Scand* 2011; **123**: 147–53.
272 Duraiswamy G, Thirthalli J, Nagendra HR, Gangadhar BN. Yoga therapy as an add-on treatment in the management of patients with schizophrenia - A randomized controlled trial. *Acta Psychiatr Scand* 2007; **116**: 226–32.
273 Falkai P, Malchow B, Wobrock T, *et al.* The effect of aerobic exercise on cortical architecture in patients with chronic schizophrenia: A randomized controlled MRI study. *Eur Arch Psychiatry Clin Neurosci* 2013; **263**: 469–73.
274 Gholipour a., Abolghasemi SH, Gholinia K, Taheri S. Token reinforcement therapeutic approach is more effective than exercise for controlling

negative symptoms of schizophrenic patients: A randomized controlled trial. *Int J Prev Med* 2012; **3**: 466–70.
275 Ho RTH, Fong TCT, Wan AHY, *et al.* A randomized controlled trial on the psychophysiological effects of physical exercise and Tai-chi in patients with chronic schizophrenia. *Schizophr Res* 2016; **171**: 42–9.
276 Q.-L. H, L.-H. X, X.-H. G. Effect of music-sport therapy on the insight and behavioral disturbance in patients with schizophrenia. *Chinese J Clin Rehabil* 2004; **8**: 1626–7.
277 Ikai S, Uchida H, Suzuki T, Tsunoda K, Mimura M, Fujii Y. Effects of yoga therapy on postural stability in patients with schizophrenia-spectrum disorders: A single-blind randomized controlled trial. *J Psychiatr Res* 2013; **47**: 1744–50.
278 Kaltsatou a., Kouidi E, Fountoulakis K, *et al.* Effects of exercise training with traditional dancing on functional capacity and quality of life in patients with schizophrenia: A randomized controlled study. *Clin Rehabil* 2014. DOI:10.1177/0269215514564085.
279 Kim H, Song B, So B, Lee O, Song W, Kim Y. Increase of circulating BDNF levels and its relation to improvement of physical fitness following 12 weeks of combined exercise in chronic patients with schizophrenia: A pilot study. *Psychiatry Res* 2014; **220**: 792–6.
280 Kimhy D, Vakhrusheva J, Bartels MN, *et al.* The Impact of Aerobic Exercise on Brain-Derived Neurotrophic Factor and Neurocognition in Individuals With Schizophrenia: A Single-Blind, Randomized Clinical Trial. *Schizophr Bull* 2015; **41**: 859–68.
281 Lin J, Chan SKW, Lee EHM, *et al.* Aerobic exercise and yoga improve neurocognitive function in women with early psychosis. *Nat Publ Gr* 2015; : 1–7.
282 Loh SY, Abdullah A, Abu Bakar AK, Thambu M, Nik Jaafar NR. Structured Walking and Chronic Institutionalized Schizophrenia Inmates: A pilot RCT Study on Quality of Life. *Glob J Health Sci* 2016; **8**: 238–48.
283 Manjunath RB, Varambally S, Thirthalli J, Basavaraddi I V, Gangadhar BN. Efficacy of yoga as an add-on treatment for in-patients with functional psychotic disorder. *Indian J Psychiatry* 2013; **55**: S374-8.
284 Martin LAL, Koch SC, Hirjak D, Fuchs T. Overcoming disembodiment: The effect of movement therapy on negative symptoms in schizophrenia-a multicenter randomized controlled trial. *Front Psychol* 2016; **7**: 1–14.
285 Marzolini S, Jensen B, Melville P. Feasibility and effects of a group-based resistance and aerobic exercise program for individuals with severe schizophrenia: A multidisciplinary approach. *Ment Health Phys Act* 2009; **2**: 29–36.
286 Oertel-Knöchel V, Mehler P, Thiel C, *et al.* Effects of aerobic exercise on cognitive performance and individual psychopathology in depressive and schizophrenia patients. *Eur Arch Psychiatry Clin Neurosci* 2014; **264**: 589–604.
287 Paikkatt B, Singh AR, Singh PK, Jahan M. Efficacy of yoga therapy on subjective well-being and basic living skills of patients having chronic schizophrenia. *Ind Psychiatry J* 2012; **21**: 109–14.
288 Pajonk F-G, Wobrock T, Gruber O, *et al.* Hippocampal plasticity in response to exercise in schizophrenia. *Arch Gen Psychiatry* 2010; **67**: 133–43.
289 Scheewe TW, van Haren NEM, Sarkisyan G, *et al.* Exercise therapy, cardiorespiratory fitness and their effect on brain volumes: A randomised controlled trial in patients with schizophrenia and healthy controls. *Eur Neuropsychopharmacol* 2012; **c**: 675–85.
290 Scheewe TW, Backx FJG, Takken T, *et al.* Exercise therapy improves mental and physical health in schizophrenia: A randomised controlled trial. *Acta Psychiatr Scand* 2013; **127**: 464–73.
291 Svatkova A, Mandl RCW, Scheewe TW, Cahn W, Kahn RS, Hulshoff Pol HE. Physical Exercise Keeps the Brain Connected: Biking Increases White Matter Integrity in Patients With Schizophrenia and Healthy Controls. *Schizophr Bull* 2015; **41**: 869–78.
292 R.E. U, R. A, P. C, *et al.* Efficacy of the guidelines for diagnosis and treatment of adolescents with schizophrenia: A comparative study vs usual treatment. *Schizophr Bull* 2013; **39**: S355.
293 Varambally S, Thirthalli J, Venkatasubramanian G, *et al.* Therapeutic efficacy of add-on yogasana intervention in stabilized outpatient schizophrenia:

Randomized controlled comparison with exercise and waitlist. *Indian J Psychiatry* 2012; **54**: 227.
294 Visceglia E, Lewis S. Yoga therapy as an adjunctive treatment for schizophrenia: a randomized, controlled pilot study. *J Altern Complement Med* 2011; **17**: 601–7.
295 Zwick S, Brunnauer a., Laux G. P03-155 - Effects of aerobic endurance training on neurocognitive functions in schizophrenic inpatients. *Eur Psychiatry* 2010; **25**: 1136.
296 Babyak M, Blumenthal JA, Herman S, *et al.* Exercise Treatment for Major Depression: Maintenance of Therapeutic Benefit at 10 Months. *Psychosom Med* 2000; **62**: 633–8.
297 Blumenthal J, Babyak M, Moore K. Effects of exercise training on older patients with major depression. *Arch Intern Med* 1999; **159**: 2349–56.
298 Blumenthal JA, Babyak MA, Doraiswamy M, *et al.* Exercise and Pharmacotherapy in the Treatment of Major Depressive Disorder. *Psychosom Med* 2007; **69**: 587–96.
299 Brenes GA, Williamson JD, Messier SP, *et al.* Treatment of minor depression in older adults: a pilot study comparing sertraline and exercise. *Aging Ment Health* 2007; **11**: 61–8.
300 Carneiro LSF, Fonseca AM, Vieira-Coelho MA, Mota MP, Vasconcelos-Raposo J. Effects of structured exercise and pharmacotherapy vs. pharmacotherapy for adults with depressive symptoms: A randomized clinical trial. *J Psychiatr Res* 2015; **71**: 48–55.
301 Carneiro LSF, Mota MP, Vieira-Coelho MA, Alves RC, Fonseca AM, Vasconcelos-Raposo J. Monoamines and cortisol as potential mediators of the relationship between exercise and depressive symptoms. *Eur Arch Psychiatry Clin Neurosci* 2016; : 1–5.
302 Carta MG, Hardoy MC, Pilu A, *et al.* Improving physical quality of life with group physical activity in the adjunctive treatment of major depressive disorder. *Clin Pract Epidemiol Ment Heal* 2008; **4**: 1.
303 Carter T, Guo B, Turner D, *et al.* Preferred intensity exercise for adolescents receiving treatment for depression: A pragmatic randomised controlled trial. *BMC Psychiatry* 2015; **15**: 1–12.
304 Cecchini-Estrada J-A, Méndez-Giménez A, Cecchini C, Moulton M, Rodríguez C. Exercise and Epstein's TARGET for treatment of depressive symptoms: A randomized study. *Int J Clin Heal Psychol* 2015. DOI:10.1016/j.ijchp.2015.05.001.
305 Chan AS, Cheung MC, Tsui WJ, Sze SL, Shi D. Dejian mind-body intervention on depressive mood of community-dwelling adults: A randomized controlled trial. *Evidence-based Complement Altern Med* 2011; **2011**. DOI:10.1093/ecam/nep043.
306 Chan AS, Wong QY, Sze SL, Kwong PPK, Han YMY, Cheung MC. A Chinese Chan-based mind-body intervention for patients with depression. *J Affect Disord* 2012; **142**: 283–9.
307 Chan AS, Han YMY, Sze SL, Wong QY, Cheung M. A Randomized Controlled Neurophysiological Study of a Chinese Chan-Based Mind-Body Intervention in Patients with Major Depressive Disorder. *Evidence-Based Complement Altern Med* 2013; **2013**. DOI:10.1155/2013/812096.
308 Chou KL, Lee PWH, Yu ECS, *et al.* Effect of Tai Chi on depressive symptoms amongst Chinese older patients with depressive disorders: A randomized clinical trial. *Int J Geriatr Psychiatry* 2004; **19**: 1105–7.
309 Combs K, Smith PJ, Sherwood A, *et al.* Impact of sleep complaints and depression outcomes among participants in the standard medical intervention and long-term exercise study of exercise and pharmacotherapy for depression. *J Nerv Ment Dis* 2014; **202**: 167–71.
310 Danielsson L, Papoulias I, Petersson EL, Carlsson J, Waern M. Exercise or basic body awareness therapy as add-on treatment for major depression: A controlled study. *J Affect Disord* 2014; **168**: 98–106.
311 Doose M, Ziegenbein M, Hoos O, *et al.* Self-selected intensity exercise in the treatment of major depression: A pragmatic RCT. *Int J Psychiatry Clin Pract* 2015; **19**: 266–76.

312 Dunn AL, Trivedi MH, Kampert JB, Clark CG, Chambliss HO. Exercise treatment for depression: Efficacy and dose response. *Am J Prev Med* 2005; **28**: 1–8.
313 Hoffman BM, Blumenthal JA, Babyak MA, *et al.* Exercise fails to improve neurocognition in depressed middle-aged and older adults. *Med Sci Sports Exerc* 2008; **40**: 1344–52.
314 Hoffman BM, Babyak MA, Craighead WE, *et al.* Exercise and pharmacotherapy in patients with major depression: one-year follow-up of the SMILE study. *Psychosom Med* 2011; **73**: 127–33.
315 Huang T-T, Liu C-B, Tsai Y-H, Chin Y-F, Wong C-H. Physical fitness exercise vs. cognitive behavior therapy on reducing the depressive symptoms among community-dwelling elderly adults: A randomized controlled trial. *Int J Nurs Stud* 2015; **52**: 1542–52.
316 Hughes CW, Barnes S, Barnes C, Defina LF, Nakonezny P, Emslie GJ. Depressed Adolescents Treated with Exercise (DATE): A pilot randomized controlled trial to test feasibility and establish preliminary effect sizes. *Ment Health Phys Act* 2013; **6**: 119–31.
317 Kerling A, Tegtbur U, G??tzlaff E, *et al.* Effects of adjunctive exercise on physiological and psychological parameters in depression: A randomized pilot trial. *J Affect Disord* 2015; **177**: 1–6.
318 Kerse N, Hayman KJ, Moyes SA, *et al.* Home-based activity program for older people with depressive symptoms: DeLLITE–a randomized controlled trial. *Ann Fam Med* 2010; **8**: 214–23.
319 Khatri P, Blumenthal JA, Babyak MA, *et al.* Effects of Exercise Training on Cognitive Functioning Among Depressed Older Men and Women. J. Aging Phys. Act. 2001; **9**: 43–57.
320 Kinser PA, Bourguignon C, Whaley D, Hauenstein E, Taylor AG. Feasibility, Acceptability, and Effects of Gentle Hatha Yoga for Women With Major Depression: Findings From a Randomized Controlled Mixed-Methods Study. *Arch Psychiatr Nurs* 2013; **27**: 137–47.
321 Kinser PA, Elswick RK, Kornstein S. Potential Long-Term Effects of a Mind-Body Intervention for Women With Major Depressive Disorder: Sustained Mental Health Improvements With a Pilot Yoga Intervention. *Arch Psychiatr Nurs* 2014; **28**: 377–83.
322 Knubben K, Reischies FM, Adli M, Schlattmann P, Bauer M, Dimeo F. A randomised, controlled study on the effects of a short-term endurance training programme in patients with major depression. *Br J Sports Med* 2007; **41**: 29–33.
323 Krogh J, Saltin B, Gluud C, Nordentoft M. The DEMO trial: A randomized, parallel-group, observer-blinded clinical trial of strength versus aerobic versus relaxation training for patients with mild to moderate depression. *J Clin Psychiatry* 2009; **70**: 790–800.
324 Krogh J, Videbech P, Thomsen C, Gluud C, Nordentoft M. DEMO-II Trial. Aerobic Exercise versus Stretching Exercise in Patients with Major Depression-A Randomised Clinical Trial. *PLoS One* 2012; **7**. DOI:10.1371/journal.pone.0048316.
325 Lavretsky H, Alstein LL, Olmstead RE, *et al.* Complementary Use of Tai Chi Chih Augments Escitalopram Treatment of Geriatric Depression: A Randomized Controlled Trial. *Am J Geriatr Psychiatry* 2011; **19**: 839–50.
326 Legrand FD. Effects of Exercise on Physical Self-Concept , Global Self-Esteem , and Depression in Women of Low Socioeconomic Status With Elevated Depressive Symptoms Effects of Exercise on Physical Self-Concept , Global Self-Esteem , and Depression in Women of Low So. 2015; : 357–65.
327 Legrand FD, Neff EM. Efficacy of exercise as an adjunct treatment for clinically depressed inpatients during the initial stages of antidepressant pharmacotherapy: An open randomized controlled trial. *J Affect Disord* 2016; **191**: 139–44.
328 Luttenberger K, Stelzer E-M, Först S, Schopper M, Kornhuber J, Book S. Indoor rock climbing (bouldering) as a new treatment for depression: study design of a waitlist-controlled randomized group pilot study and the first results. *BMC Psychiatry* 2015; **15**: 201.
329 Martiny K, Refsgaard E, Lund V, *et al.* Maintained superiority of chronotherapeutics vs. exercise in a 20-week randomized follow-up trial in major

depression. *Acta Psychiatr Scand* 2015; **131**: 446–57.
330 Mather AS, Rodriguez C, Guthrie MF, McHarg AM, Reid IC, McMurdo MET. Effects of exercise on depressive symptoms in older adults with poorly responsive depressive disorder: Randomised controlled trial. *Br J Psychiatry* 2002; **180**: 411–5.
331 Mota-Pereira J, Silverio J, Carvalho S, Ribeiro JC, Fonte D, Ramos J. Moderate exercise improves depression parameters in treatment-resistant patients with major depressive disorder. *J Psychiatr Res* 2011; **45**: 1005–11.
332 Belvederi Murri M, Amore M, Menchetti M, *et al.* Physical exercise for late-life major depression. *Br J Psychiatry* 2015; **207**: 235–42.
333 Nabkasorn C, Miyai N, Sootmongkol A, *et al.* Effects of physical exercise on depression, neuroendocrine stress hormones and physiological fitness in adolescent females with depressive symptoms. *Eur J Public Health* 2006; **16**: 179–84.
334 Niemi M, Kiel S, Allebeck P, Hoan LT. Community-based intervention for depression management at the primary care level in Ha Nam Province, Vietnam: A cluster-randomised controlled trial. *Trop Med Int Heal* 2016; **21**: 654–61.
335 Pfaff JJ, Alfonso H, Newton RU, Sim M, Flicker L, Almeida OP. ACTIVEDEP: a randomised, controlled trial of a home-based exercise intervention to alleviate depression in middle-aged and older adults. *Br J Sports Med* 2014; **48**: 226–32.
336 Pilu A, Sorba M, Hardoy MC, *et al.* Efficacy of physical activity in the adjunctive treatment of major depressive disorders: preliminary results. *Clin Pract Epidemiol Ment Heal* 2007; **3**: 1.
337 Prakhinkit S, Suppapitiporn S, Tanaka H, Suksom D. Effects of Buddhism Walking Meditation on Depression, Functional Fitness, and Endothelium-Dependent Vasodilation in Depressed Elderly. *J Altern Complement Med* 2014; **20**: 411–6.
338 Schuch FB arreto, Vasconcelos-Moreno MP aiva, Borowsky C, *et al.* The effects of exercise on oxidative stress (TBARS) and BDNF in severely depressed inpatients. *Eur Arch Psychiatry Clin Neurosci* 2014; **264**: 605–13.
339 Schuch FB, Vasconcelos-Moreno MP, Borowsky C, Zimmermann AB, Rocha NS, Fleck MP. Exercise and severe major depression: Effect on symptom severity and quality of life at discharge in an inpatient cohort. *J Psychiatr Res* 2015; **61**: 25–32.
340 Shahidi M, Mojtahed A, Modabbernia A, *et al.* Laughter yoga versus group exercise program in elderly depressed women: A randomized controlled trial. *Int J Geriatr Psychiatry* 2011; **26**: 322–7.
341 Sims J, Hill K, Davidson S, Gunn J, Huang N. Exploring the feasibility of a community-based strength training program for older people with depressive symptoms and its impact on depressive symptoms. *BMC Geriatr* 2006; **6**: 18.
342 Singh NA, Clements KM, Fiatarone MA. Sleep, sleep deprivation, and daytime activities - A randomized controlled trial of the effect of exercise on sleep. *Sleep* 1997; **20**: 95–101.
343 Singh NA, Clements KM, Fiatarone MA. A randomized controlled trial of progressive resistance training in depressed elders. *Journals Gerontol Ser A-Biological Sci Med Sci* 1997; **52**: M27-35.
344 Singh N a, Clements KM, Singh M a. The efficacy of exercise as a long-term antidepressant in elderly subjects: a randomized, controlled trial. *journals Gerontol* 2001; **56**: M497–504.
345 Singh NA, Stavrinos TA, Scarbek Y, Galambos G, Liber C, Singh MAF. A randomized controlled trial of high versus low intensity weight training versus general practitioner care for clinical depression in older adults. *Journals Gerontol Ser A-Biological Sci Med Sci* 2005; **60**: 768–76.
346 Siqueira CC, Valiengo LL, Carvalho AF, *et al.* Antidepressant efficacy of adjunctive aerobic activity and associated biomarkers in major depression: A 4-week, randomized, single-blind, controlled clinical trial. *PLoS One* 2016; **11**: 1–12.
347 Tsang HWH, Fung KMT, Chan ASM, Lee G, Chan F. Effect of a qigong exercise programme on elderly with depression. *Int J Geriatr Psychiatry* 2006; **21**: 890–7.

348 Tsang HWH, Tsang WWN, Jones AYM, *et al.* Psycho-physical and neurophysiological effects of qigong on depressed elders with chronic illness. *Aging Ment Health* 2012; **17**: 1–13.
349 Veale D, Le Fevre K, Pantelis C, de Souza V, Mann A, Sargeant A. Aerobic exercise in the adjunctive treatment of depression: a randomized controlled trial. *J R Soc Med* 1992; **85**: 541–4.
350 Yeung A, Lepoutre V, Wayne P, *et al.* Tai chi treatment for depression in Chinese Americans: a pilot study. *Am J Phys Med Rehabil* 2012; **91**: 863–70.
351 Jensen CS, Portelius E, Siersma V, *et al.* Cerebrospinal fluid amyloid beta and tau concentrations are not modulated by 16 weeks of moderate- to high-intensity physical exercise in patients with Alzheimer disease. *Dement Geriatr Cogn Disord* 2016; **42**: 146–58.
352 Jensen CS, Portelius E, Høgh P, *et al.* Effect of physical exercise on markers of neuronal dysfunction in cerebrospinal fluid in patients with Alzheimer's disease. *Alzheimer's Dement Transl Res Clin Interv* 2017; **3**: 284–90.
353 Morris JK, Vidoni ED, Johnson DK, *et al.* Aerobic exercise for Alzheimer's disease: A randomized controlled pilot trial. *PLoS One* 2017; **12**: 1–14.
354 Vidoni ED, Perales J, Alshehri M, Giles A-M, Siengsukon CF, Burns JM. Aerobic Exercise Sustains Performance of Instrumental Activities of Daily Living in Early-Stage Alzheimer Disease. *J Geriatr Phys Ther* 2017; : 1.
355 Sopina E, Sørensen J, Beyer N, Hasselbalch SG, Waldemar G. Cost-effectiveness of a randomised trial of physical activity in Alzheimer's disease: A secondary analysis exploring patient and proxy-reported health-related quality of life measures in Denmark. *BMJ Open* 2017; **7**: 1–11.
356 Lanza G, Centonze SS, Destro G, *et al.* Shiatsu as an adjuvant therapy for depression in patients with Alzheimer's disease: A pilot study. *Complement Ther Med* 2018; **38**: 74–8.
357 Pedrinolla A, Venturelli M, Fonte C, *et al.* Exercise Training on Locomotion in Patients with Alzheimer's Disease: A Feasibility Study. *J Alzheimers Dis* 2018; **61**: 1599–609.
358 Venturelli M, Sollima A, Ce E, *et al.* Effectiveness of Exercise- and Cognitive-Based Treatments on Salivary Cortisol Levels and Sundowning Syndrome Symptoms in Patients with Alzheimer's Disease. *J Alzheimers Dis* 2016; **53**: 1631–40.
359 Busse M, Quinn L, Drew C, *et al.* Physical Activity Self-Management and Coaching Compared to Social Interaction in Huntington Disease: Results From the ENGAGE-HD Randomized, Controlled Pilot Feasibility Trial. *Phys Ther* 2017; **97**: 625–39.
360 Quinn L, Hamana K, Kelson M, *et al.* A randomized, controlled trial of a multi-modal exercise intervention in Huntington's disease. *Park Relat Disord* 2016; **31**: 46–52.
361 Afrasiabifar A, Karami F, Najafi Doulatabad S. Comparing the effect of Cawthorne–Cooksey and Frenkel exercises on balance in patients with multiple sclerosis: a randomized controlled trial. *Clin Rehabil* 2018; **32**: 57–65.
362 Amiri, B, Sahebozamani, M, Sedighi, B. The effects of 10-week core stability training on balance in women with Multiple Sclerosis according to Expanded Disability Status Scale: a single-blinded randomized controlled trial. *Eur J Phys Rehabil Med* 2018; **02**: 02.
363 Barghi A, Allendorfer JB, Taub E, *et al.* Phase II Randomized Controlled Trial of Constraint-Induced Movement Therapy in Multiple Sclerosis. Part 2: Effect on White Matter Integrity. *Neurorehabil Neural Repair* 2018; **32**: 233–41.
364 Briken S, Rosenkranz SC, Keminer O, *et al.* Effects of exercise on Irisin, BDNF and IL-6 serum levels in patients with progressive multiple sclerosis. *J Neuroimmunol* 2016; **299**: 53–8.
365 Carling A, Forsberg A, Gunnarsson M, Nilsagård Y. CoDuSe group exercise programme improves balance and reduces falls in people with multiple sclerosis: A multi-centre, randomized, controlled pilot study. *Mult Scler* 2017; **23**: 1394–404.
366 Coghe, G, Corona, F, Marongiu, E, *et al.* Fatigue, as measured using the Modified Fatigue Impact Scale, is a predictor of processing speed improvement induced by exercise in patients with multiple sclerosis: data from a randomized controlled trial. *J Neurol* 2018; : 1–6.

367 Duff WRD, Andrushko JW, Renshaw DW, *et al.* Impact of pilates exercise in multiple sclerosis: A randomized controlled trial. *Int J MS Care* 2018; **20**: 92–100.
368 Forsberg A, von Koch L, Nilsagard Y. Effects on Balance and Walking with the CoDuSe Balance Exercise Program in People with Multiple Sclerosis: A Multicenter Randomized Controlled Trial. *Mult Scler Int* 2016; **2016**: 7076265.
369 Heine M, Verschuren O, Hoogervorst E, *et al.* Does aerobic training alleviate fatigue and improve societal participation in patients with multiple sclerosis? The TREFAMS-A multicentre randomised trial. *Mult Scler* 2016; **22**: 396–7.
370 Kargarfard M, Shariat A, Ingle L, Cleland JA, Kargarfard M. Randomized Controlled Trial to Examine the Impact of Aquatic Exercise Training on Functional Capacity, Balance, and Perceptions of Fatigue in Female Patients With Multiple Sclerosis. *Arch Phys Med Rehabil* 2018; **99**: 234–41.
371 Learmonth YC, Adamson BC, Kinnett-Hopkins D, Bohri M, Motl RW. Results of a feasibility randomised controlled study of the guidelines for exercise in multiple sclerosis project. *Contemp Clin Trials* 2017; **54**: 84–97.
372 Ortiz-Rubio A, Cabrera-Martos I, Rodríguez-Torres J, Fajardo-Contreras W, Díaz-Pelegrina A, Valenza MC. Effects of a Home-Based Upper Limb Training Program in Patients With Multiple Sclerosis: A Randomized Controlled Trial. *Arch Phys Med Rehabil* 2016; **97**: 2027–33.
373 Pau M, Corona F, Coghe G, *et al.* Quantitative assessment of the effects of 6 months of adapted physical activity on gait in people with multiple sclerosis: a randomized controlled trial. *Disabil Rehabil* 2018; **40**: 144–51.
374 Sandroff BM, Johnson CL, Motl RW. Exercise training effects on memory and hippocampal viscoelasticity in multiple sclerosis: a novel application of magnetic resonance elastography. *Neuroradiology* 2017; **59**: 61–7.
375 Sandroff BM, Bollaert RE, Pilutti LA, *et al.* Multimodal exercise training in multiple sclerosis: A randomized controlled trial in persons with substantial mobility disability. *Contemp Clin Trials* 2017; **61**: 39–47.
376 Vermöhlen V, Schiller P, Schickendantz S, *et al.* Hippotherapy for patients with multiple sclerosis: A multicenter randomized controlled trial (MS-HIPPO). *Mult Scler J* 2017; : 1375–82.
377 Carroll LM, Volpe D, Morris ME, Saunders J, Clifford AM. Aquatic Exercise Therapy for People With Parkinson Disease: A Randomized Controlled Trial. *Arch Phys Med Rehabil* 2017; **98**: 631–8.
378 Cheng F-Y, Yang Y-R, Chen L-M, Wu Y-R, Cheng S-J, Wang R-Y. Positive Effects of Specific Exercise and Novel Turning-based Treadmill Training on Turning Performance in Individuals with Parkinson's disease: A Randomized Controlled Trial. *Sci Rep* 2016; **6**: 33242.
379 Hubble RP, Naughton G, Silburn PA, Cole MH. Trunk Exercises Improve Gait Symmetry in Parkinson Disease: A Blind Phase II Randomized Controlled Trial. *Am J Phys Med Rehabil* 2018; **97**: 151–9.
380 Kanegusuku H, Silva-Batista C, Peçanha T, *et al.* Effects of Progressive Resistance Training on Cardiovascular Autonomic Regulation in Patients With Parkinson Disease: A Randomized Controlled Trial. *Arch Phys Med Rehabil* 2017; **98**: 2134–41.
381 Lee HJ, Kim SY, Chae Y, *et al.* Turo (Qi Dance) Program for Parkinson's Disease Patients: Randomized, Assessor Blind, Waiting-List Control, Partial Crossover Study. *Explore* 2018; **14**: 216–23.
382 Liu XL, Chen S, Wang Y. Effects of health qigong exercises on relieving symptoms of Parkinson's disease. *Evidence-based Complement Altern Med* 2016; **2016**. DOI:10.1155/2016/5935782.
383 Mollinedo I, Cancela JM, Vila Suarez MH. Effect of a Mat Pilates Program with TheraBand® on Dynamic Balance in Patients with Parkinson's Disease. Feasibility Study and Randomized Controlled Trial. *Rejuvenation Res* 2017; **XX**: rej.2017.2007.
384 Paolucci T, Zangrando F, Piccinini G, *et al.* Impact of Mézières Rehabilitative Method in Patients with Parkinson's Disease: A Randomized Controlled Trial. *Park Dis* 2017; **2017**: 11.

385 Suhaila M. SANTOS , Rubens A. da SILVA MBT, Isabela A. ALMEIDA , Lúcio B. de MELO HBF. Balance versus resistance training on postural control in patients with Parkinson's disease: a randomized controlled trial. *Eur J Phys Rehabil Med* 2017; **5353**: 173–83.
386 Santos L, Fernandez-Rio J, Winge K, *et al.* Effects of progressive resistance exercise in akinetic-rigid Parkinson's disease patients: a randomized controlled trial. *Eur J Phys Rehabil Med* 2017; **53**: 651–63.
387 Silva-Batista C, de Brito LC, Corcos DM, *et al.* Resistance Training Improves Sleep Quality in Subjects With Moderate Parkinson's Disease. *J strength Cond Res* 2017; **31**: 2270–7.
388 Vergara-Diaz G, Osypiuk K, Hausdorff JM, *et al.* Tai Chi for Reducing Dual-task Gait Variability, a Potential Mediator of Fall Risk in Parkinson's Disease: A Pilot Randomized Controlled Trial. *Glob Adv Heal Med* 2018; **7**: 216495611877538.
389 Ortiz-Rubio A, Cabrera-Martos I, Torres-Sanchez I, Casilda-Lopez J, Lopez-Lopez L, Valenza MC. Effects of a resistance training program on balance and fatigue perception in patients with Parkinson's disease: A randomized controlled trial. *Med Clin (Barc)* 2018; **150**: 460–4.
390 Bhatia T, Mazumdar S, Wood J, *et al.* A randomised controlled trial of adjunctive yoga and adjunctive physical exercise training for cognitive dysfunction in schizophrenia. *Acta Neuropsychiatr* 2017; **29**: 102–14.
391 Lin J, Geng X, Lee EH, *et al.* Yoga reduces the brain's amplitude of low-frequency fluctuations in patients with early psychosis results of a randomized controlled trial. *Schizophr Res* 2017; **184**: 141–2.
392 Buschert V, Prochazka D, Bartl H, *et al.* Effects of physical activity on cognitive performance: a controlled clinical study in depressive patients. *Eur Arch Psychiatry Clin Neurosci* 2018; published online June. DOI:10.1007/s00406-018-0916-0.
393 Kerling A, Kück M, Tegtbur U, *et al.* Exercise increases serum brain-derived neurotrophic factor in patients with major depressive disorder. *J Affect Disord* 2017; **215**: 152–5.
394 Kerling A, Hartung D, Stubbs B, *et al.* Impact of aerobic exercise on muscle mass in patients with major depressive disorder: a randomized controlled trial. *Neuropsychiatr Dis Treat* 2018; **14**: 1969–74.
395 Olson RL, Brush CJ, Ehmann PJ, Alderman BL. Clinical Neurophysiology A randomized trial of aerobic exercise on cognitive control in major depression. *Clin Neurophysiol* 2017; **128**: 903–13.
396 Prathikanti S, Rivera R, Cochran A, Tungol JG, Fayazmanesh N, Weinmann E. Treating major depression with yoga: A prospective, randomized, controlled pilot trial. *PLoS One* 2017; **12**: e0173869.
397 Sharma A, Barrett MS, Cucchiara AJ, Gooneratne NS, Thase ME. A Breathing-Based Meditation Intervention for Patients With Major Depressive Disorder Following Inadequate Response to Antidepressants: A Randomized Pilot Study. *J Clin Psychiatry* 2017; **78**: e59–63.
398 Yeung AS, Feng R, Kim DJH, *et al.* A Pilot, Randomized Controlled Study of Tai Chi With Passive and Active Controls in the Treatment of Depressed Chinese Americans. *J Clin Psychiatry* 2017; **78**: e522–8.
399 Tolahunase MR, Sagar R, Faiq M, Dada R. Yoga- and meditation-based lifestyle intervention increases neuroplasticity and reduces severity of major depressive disorder: A randomized controlled trial. *Restor Neurol Neurosci* 2018; **36**: 423–42.
400 Lamb SE, Sheehan B, Atherton N, *et al.* Dementia And Physical Activity (DAPA) trial of moderate to high intensity exercise training for people with dementia: randomised controlled trial. *BMJ* 2018; **361**: k1675.
