# Supplementary material for: Physical exercise improves quality of life, depressive symptoms, and cognition across chronic brain disorders: a transdiagnostic systematic review and meta-analysis of randomized controlled trials
Source: J Neurol. 2019 Aug 14;268(4):1222–46. doi: 10.1007/s00415-019-09493-9 (PMC7990819; doi:10.1007/s00415-019-09493-9)
Supplement: Supplementary file 5 — Supplementary file5 (PDF 329 kb) [file 415_2019_9493_MOESM5_ESM.pdf]

Physical exercise improves quality of life, depressive symptoms, and cognition across chronic brain disorders: a transdiagnostic systematic review and meta-analysis of randomized controlled trials

Meenakshi Dauwan*, Marieke JH Begemann, Margot IE Slot, Edwin HM Lee, Philip Scheltens, Iris EC Sommer

*** Corresponding author:**
Meenakshi Dauwan, M.D.
Neuroimaging Center, University Medical Center Groningen
Department of Clinical Neurophysiology and MEG Center, Amsterdam UMC, Vrije Universiteit Amsterdam
Department of Psychiatry, University Medical Center Utrecht
Neuroimaging Center 3111
Antonius Deusinglaan 2
9713 AW Groningen, The Netherlands
Tel: +31 88 75 57468
E-mail: m.dauwan@umcg.nl; m.dauwan-3@umcutrecht.nl

Table S4: Risk of bias assessment of studies eligible for meta-analysis

	Random sequence generation (selection bias)	Allocation concealment (selection bias)	Baseline imbalance	Blinding of outcome assessment (detection bias)	Incomplete outcome data (attrition bias)	Intention to treat (ITT) analysis (attrition bias)	Selective reporting (reporting bias)
**Alzheimer's disease**							
Aguiar 2014	+	+	+	+	+	-	+

Arcoverde 2014	?	?	+	+	+	?	+
Hoffmann 2015	+	?	+	+	+	+	+
Holthoff 2015	?	?	+	+	+	+	+
Kemoun 2010	+	?	+	?	+	?	+
Lautenschlager 2015	?	?	?	?	+	+	-
Maci 2012	+	?	+	+	+	+	+
Ohman 2016a	+	?	+	-	-	?	-
Roach 2011	?	?	+	+	-	?	-
Rolland 2007	+	?	+	+	+	-	+
Steinberg 2009	?	?	-	+	?	+	+
Suttanon 2012	+	+	?	+	+	+	+
Teri 2003	+	?	+	+	+	+	-
Venturelli 2011	+	?	+	+	+	?	-
Vreugdenhil 2012	?	?	+	+	?	+	+
Yaguez 2011	+	?	+	-	+	-	+
Yang 2015	?	?	?	?	?	?	?
Zhang 2004	?	?	?	?	?	?	?
**Huntington's disease**							
Busse 2013	+	?	?	+	+	-	+
Busse 2017	+	?	+	+	+	-	+
Khalil 2013	+	+	?	-	+	-	+
Quinn 2014	+	+	?	+	+	-	+
Quinn 2016	+	+	?	+	+	+	+
Thompson 2013	?	?	+	+	+	?	+
**Multiple Sclerosis**							
Ahmadi 2010a	?	?	+	?	+	+	+
Ahmadi 2010b	?	?	+	?	+	+	+
Ahmadi 2013	?	?	+	?	+	+	+
Bernhardt 2012	?	?	?	?	?	?	?
Bjarnadottir 2007	?	+	+	+	?	-	+
Briken 2014	+	?	?	-	+	-	+
Bulguroglu 2015	?	?	?	+	?	?	?
Cakit 2010	+	?	+	+	-	-	+
Carter 2014	+	+	+	+	+	+	+
Coghe 2018	?	?	+	+	+	?	+
Dalgas 2010b	?	?	+	-	-	-	+
Dodd 2011	+	+	+	+	+	+	+
Doulatabad 2013	?	?	?	?	?	?	+
Duff 2018	+	+	+	+	+	+	+
Ebrahimi 2015	-	-	+	+	?	-	+
Feys 2016	?	?	?	?	?	?	?

Garrett 2012a	+	+	+	+	+	-	+
Hebert 2012	?	?	+	+	+	+	+
Hoang 2015	+	+	+	+	-	-	+
Hogan 2014	-	-	-	+	-	-	+
Jäckel 2015	?	?	?	?	?	?	?
Kargarfard 2012	?	?	+	+	-	?	+
Khan 2008	+	?	+	?	?	-	+
Kooshiar 2015	?	?	?	?	?	?	?
Learmonth 2012	+	?	+	+	-	?	+
Learmonth 2017	?	+	+	?	+	-	+
Louie 2015	?	?	?	+	?	?	?
McCullagh 2008	-	?	+	-	-	-	+
Miller 2011	?	?	+	+	?	?	+
Negahban 2013	+	?	+	+	+	+	+
Nilsagard 2013	+	+	+	+	+	-	+
O'Donnell 2011	?	?	?	+	?	?	?
Oken 2004	+	?	+	+	-	-	+
Ozgen 2016	?	?	+	+	+	+	+
Paul 2014	+	?	+	?	+	-	+
Petajan 1996	?	?	+	?	?	-	+
Plow 2014	+	+	+	-	+	+	+
Prosperini 2013	+	?	+	+	-	-	+
Rahnama 2011	?	?	?	?	?	?	?
Razazian 2016	+	?	+	+	?	?	+
Rietberg 2014	+	+	+	+	+	-	+
Romberg 2005	?	?	+	-	+	+	+
Salhofer-Polanyi 2013	?	?	+	+	?	?	+
Sandroff 2016	+	?	+	+	+	+	?
Sandroff 2017a	?	?	?	+	+	+	+
Sangelaji 2014	+	?	?	?	-	-	+
Storr 2006	-	-	?	+	-	-	+
Straudi 2014	+	+	+	+	+	+	+
Sutherland 2001	?	?	+	?	?	+	+
Tallner 2012	?	?	?	?	?	?	?
Tarakci 2013	+	?	+	+	-	-	+
Vermöhlen 2018	+	+	+	+	+	+	+
**Parkinson's disease**							
Allen 2010	+	+	?	+	-	-	+
Ashburn 2007	+	+	+	+	-	-	+
Belton 2014	+	+	+	+	+	+	+
Canning 2012	?	?	?	+	-	-	+

Canning 2014	+	+	+	+	-	-	+
Carroll 2017	?	+	+	?	+	?	+
Cholewa 2013	?	?	?	?	?	?	+
Clarke 2016	+	+	+	?	+	+	+
Comelia 1994	?	?	?	+	?	-	+
Conradsson 2015	+	+	+	-	+	+	+
Cugusi 2015	?	?	+	?	+	+	+
De Oliveira 2016	?	?	+	+	+	-	+
Duncan 2014	?	?	+	+	+	+	+
Foster 2013	?	?	+	+	?	+	+
Goodwin 2011	+	+	?	-	+	-	+
Keus 2007	+	?	+	+	+	+	+
Laupheimer 2011	?	?	+	?	?	-	+
Liao 2015a	?	?	+	+	+	+	+
Ni 2016a	+	?	+	-	-	-	+
Ni 2016b	+	?	+	-	-	-	+
Park 2014a	?	?	+	+	+	-	+
Picelli 2016	?	+	+	+	+	+	+
Poliakoff 2013	+	+	?	?	-	-	+
Qutubuddin 2013	?	?	+	+	?	?	+
Romenets 2015	-	?	-	-	-	+	+
Santos 2017b	?	?	+	?	+	+	+
Schmitz-Hübsch 2006	?	?	?	?	?	+	+
Sharma 2015	+	?	+	+	+	+	-
Silva-Batista 2016	?	?	+	+	+	+	+
Stack 2012	?	?	+	+	?	-	+
Tickle-Degnen 2010	+	?	+	-	+	+	+
Vergara-Diaz-2018	+	?	?	+	-	-	+
Wade 2003	+	?	+	+	-	-	+
**Schizophrenia**							
Battaglia 2013	+	?	+	+	?	?	+
Bhatia 2017	+	?	+	+	+	+	+
Ho 2016	?	?	+	-	+	+	+
Ikai 2013	+	+	+	+	+	+	+
Kaltsatou 2014	+	?	+	+	+	+	+
Kimhy 2015	?	?	+	+	?	-	+
Lin 2015	+	?	+	+	-	-	+
Loh 2016	+	+	-	?	?	+	+
Marzolini 2009	+	?	+	?	?	?	+
Visceglia 2011	+	?	+	+	?	?	+
**Unipolar Depression**							

Blumenthal 1999	?	?	+	+	+	+	+
Blumenthal 2007	+	?	+	+	+	+	+
Brenes 2007	+	?	+	+	+	+	+
Carneiro 2015	?	+	+	-	-	-	+
Carta 2008	?	?	?	-	+	+	+
Carter 2015	+	+	+	+	?	+	+
Chan 2012	?	?	+	+	?	-	+
Chou 2004	?	?	+	+	+	+	+
Danielsson 2014	+	+	+	+	+	+	+
Doose 2015	?	?	+	-	+	+	+
Hoffman 2008	?	?	+	+	+	+	+
Huang 2015	+	?	+	+	+	+	+
Kerling 2015	?	?	+	?	+	+	+
Kerse 2010	+	?	?	+	?	?	+
Khatri 2001	?	?	+	+	?	+	+
Kinser 2014	+	?	+	?	-	-	+
Lavretsky 2011	+	+	+	+	-	+	+
Legrand 2015	+	?	?	?	-	-	+
Legrand 2016	+	?	+	?	+	+	+
Luttenberger 2015	-	?	+	?	+	-	+
Mather 2002	+	+	-	+	+	+	+
Mota-Pereira 2011	?	?	-	+	+	-	+
Murri 2015	+	+	?	+	+	+	-
Nabkasorn 2006	?	?	?	+	-	-	+
Niemi 2016	+	?	+	?	+	+	+
Oertel Knöchel 2014	+	?	+	+	?	?	+
Pfaff 2014	+	?	+	+	+	+	-
Pilu 2007	?	?	?	?	?	+	+
Prakhinkit 2014	+	?	?	?	+	-	+
Prathikanti 2017	+	+	+	+	+	+	+
Schuch 2015	?	?	+	+	+	+	+
Shahidi 2011	?	?	?	?	?	-	+
Sharma 2017	+	?	+	+	+	+	-
Sims 2006	+	?	+	+	?	+	+
Singh 1997b	+	?	+	+	+	+	+
Singh 2001	+	+	+	+	+	?	+
Singh 2005	+	+	+	+	+	-	+
Siqueira 2016	+	+	-	+	-	-	+
Tolahunase 2018	?	?	?	?	?	?	?
Tsang 2006	?	?	-	+	-	-	+
Tsang 2012	+	?	+	+	+	+	+
Veale 1992	?	?	?	+	?	-	-

Yeung 2012	+	+	+	+	+	+	+
Yeung 2017	+	?	+	+	-	?	+

Risk of bias was assessed according to the *Cochrane Handbook of Systematic Reviews of Interventions*

+ = Low risk of bias

? = Unclear risk of bias

- = High risk of bias

Risk of bias assessment

Quality of life

Selection bias (random sequence generation & allocation concealment)

Forty-two (65.6%) of the 64 studies used an acceptable method of random sequence generation (most often computer-generated sequence or using a random number table) and were classified as having low risk of bias. Of this, 18 (42.9%) studies utilized acceptable methods of allocation concealment, whereas 24 (57.1%) studies did not specify the allocation concealment methods used. Eighteen (28.1%) studies did not provide sufficient information to judge the randomization and/or allocation method. Four (6.3%) studies (Ebrahimi et al., 2015; Hogan et al., 2014; Romenets et al., 2015; Storr et al., 2006) were judged as having an improper method of random sequence generation and concealment and were rated as high risk of bias.

Baseline imbalance

In general, there was good evidence of balance in baseline characteristics between groups in 50 (78.1%) of 64 studies. Twelve (18.8%) studies were classified as having unclear risk of bias because of lack of information or no full text available to judge. Two (3.1%) studies (Hogan et al., 2014; Romenets et al., 2015) reported imbalance in baseline characteristics and were rated as high risk.

Detection Bias (blinding of outcome assessment)

Blinding of personnel was achieved in 42 (65.6%) of the 64 studies. Twelve (18.8%) studies did not provide information on blinding and were classified as having unclear risk of bias. Ten (16.9%) studies (Carta et al., 2008; Dalgas et al., 2010; Goodwin et al., 2011; Khalil et al., 2013; Ni et al., 2016b, 2016a; Plow et al., 2014; Romberg, 2005; Romenets et al., 2015; Tickle-Degnen et al., 2010) stated unblinded outcome assessment and were rated as high risk.

Attrition bias (Incomplete outcome data & Intention-to-treat analysis)

Thirty-six (56.3%) studies reported complete outcome data (i.e. no drop out or in case of drop out balance in number and/or reasons for missing outcome data between intervention and control group) and 29 (45.3%) studies analyzed data using intention-to-treat (ITT) method. Ten (15.6%) studies reported insufficient information to judge incomplete outcome data whereas ten (15.6%) studies did not provide information on ITT analysis. Eighteen (28.1%) studies were classified as having high risk of bias because of high numbers of drop out and/or imbalance in number and reasons for missing outcome data. Seven studies reported that they analyzed data according to the ITT principle, but data flow chart and tables showed results for the completed groups. Twenty-five studies (39.1%) did not perform ITT analyses. These studies were rated as high risk.

Reporting bias (selective reporting)

In 62 (96.9%) of 64 studies, it was clear that published studies included all measured outcomes and were classified as having low risk of bias. We did not have full text of one study (Zhang et al., 2004) and therefore could not judge reporting bias. One (1.6%) study (Teri et al., 2003) was rated as having high risk of bias because of dissimilarity in the outcomes listed in the method section (physical health and function, and secondary outcomes on performance and caregiver reports) while no results were reported in the results section.

Depressive symptoms

Selection bias (random sequence generation & allocation concealment)

Thirty-four (56.7%) of the 60 studies used adequate randomization methods and were rated as low risk. Of this, nine (26.5%) studies applied acceptable allocation concealment. Twenty-four (40.0%) studies did not provide sufficient information to assess randomization and allocation concealment was not specified in 48 (80.0%) studies. These studies were classified as having unclear risk of bias. Two (3.5%) studies (Luttenberger et al., 2015; Romenets et al., 2015) were rated as high risk of bias because of improper method of random sequence generation.

Baseline imbalance

Forty-nine (81.7%) of 57 studies reported no imbalance in baseline characteristics and were classified as having low risk of bias. Seven (11.7%) studies were rated as unclear risk of bias. Four (6.7%) studies (Mota-Pereira et al., 2011; Romenets et al., 2015; Siqueira et al., 2016; Tsang et al., 2006) reported baseline imbalance and were judged as high risk of bias.

Detection Bias (blinding of outcome assessment)

Outcome assessment was blinded in 38 (63.3%) of 60 studies and thus these studies were judged as low risk of bias. Sixteen (26.7%) studies did not state whether outcome assessors were blinded and were rated as unclear risk of bias. In six (10.0%) studies (Briken et al., 2014; Carneiro et al., 2015; Dalgas et al., 2010; Doose et al., 2015; Romberg, 2005; Romenets et al., 2015) outcome assessment was not blinded. These studies were classified as having high risk of bias.

Attrition bias (Incomplete outcome data & Intention-to-treat analysis)

Thirty-five (58.3%) of the 60 studies reported complete outcome data (i.e. no drop out or in case of drop out balance in number and/or reasons for missing outcome data between intervention and control group) and 31 (51.7%) studies analyzed data according to the ITT principle. Thirteen (21.7%) studies did not provide sufficient information to assess incomplete outcome data. Seven (11.7%) studies did not specify the analysis method used. Twelve (20.0%) studies reported high numbers of drop out and/or imbalance in number and reasons for missing data between groups and were classified as having high risk of bias. Five studies stated that data-analyses were performed using ITT method, but this could not be confirmed with the flow chart and results of the study (e.g. different sample sizes at pre- and post-assessments within groups). Twenty-two (36.7%) studies did not analyzed data using ITT method. These studies were classified as having high risk of bias.

Reporting bias (selective reporting)

All (95.0%) studies but three (Belvederi Murri et al., 2015; Sharma et al., 2017; Teri et al., 2003) reported all mentioned outcome measures and were judged as low risk of bias. These studies were classified as having high risk of bias.

Cognition

Selection bias (random sequence generation & allocation concealment)

Twenty (55.6%) of 36 studies applied adequate random sequence generation methods and were classified as having low risk of bias. Eight (22.2%) studies applied acceptable allocation concealment. Fifteen (41.7%) studies did not specify the randomization method used whereas 28 (77.8%) studies did not provide information on the allocation method used. These studies were rates as unclear risk of bias. One (2.8%) study (Romenets et al., 2015) applied an improper method of randomization and was classified as having high risk of bias.

Baseline imbalance

In thirty (83.3%) of 36 studies baseline characteristics were balanced between the intervention and control group. These studies were rated as low risk of bias. Five (13.9%) studies were judged as unclear risk of bias, because they did not provide sufficient information on baseline data. One (2.8%) study (Romenets et al., 2015) reported baseline imbalance and was rated as high risk of bias.

Detection Bias (blinding of outcome assessment)

Twenty-seven (75.0%) of 36 studies reported blinded outcome assessment and were rated as low risk of bias. Two (5.6%) studies did not state whether outcome assessors were blinded and were judged as having unclear risk of bias. In seven (19.4%) studies (Briken et al., 2014; Conradsson et al., 2015; Ho et al., 2016; Öhman et al., 2016; Romberg, 2005; Romenets et al., 2015; Yágüez et al., 2011) outcome assessors were not masked. These studies were classified as having high risk of bias.

Attrition bias (Incomplete outcome data & Intention-to-treat analysis)

In twenty-five (69.4%) of 36 studies outcome data was reported completely (i.e. no drop out or in case of drop out balance in number and/or reasons for missing outcome data between intervention and control group). Seventeen (47.2%) studies analyzed data according to the ITT principle. These studies were classified as having low risk of bias. Six (16.7%) studies did not provide sufficient data to judge availability of outcome data whereas 8 (22.2%) studies did not specify the analysis method used. These studies were rated as unclear risk of bias. Five (13.9%) studies (Hoang et al., 2015; Lin et al., 2015; Öhman et al., 2016; Oken et al., 2004; Romenets et al., 2015) were rated as having high risk of bias because of high numbers of drop out and/or imbalance in number and/or reasons for missing data between the intervention and control group. Eleven (30.6%) studies did not conduct ITT analyses and were judged as high risk of bias.

Reporting bias (selective reporting)

Thirty-two (88.9%) studies showed results for all the mentioned outcome measures and were classified as having low risk of bias. In one (2.8%) study (Sandroff et al., 2016), it was unclear whether part of the outcome measures was assessed at both baseline and post-treatment or only at baseline. Selective reporting could not be assessed in one study because of no full text. Therefore, these studies were rated as unclear risk of bias. Two (5.6%) studies (Öhman et al., 2016; Venturelli et al., 2011) did not report results for all the mentioned outcome measures and were judged as high risk of bias.
