# Supplementary material for: Physical exercise improves quality of life, depressive symptoms, and cognition across chronic brain disorders: a transdiagnostic systematic review and meta-analysis of randomized controlled trials
Source: J Neurol. 2019 Aug 14;268(4):1222–46. doi: 10.1007/s00415-019-09493-9 (PMC7990819; doi:10.1007/s00415-019-09493-9)
Supplement: Supplementary file 6 — Supplementary file6 (PDF 112 kb) [file 415_2019_9493_MOESM6_ESM.pdf]

Physical exercise improves quality of life, depressive symptoms, and cognition across chronic brain disorders: a transdiagnostic systematic review and meta-analysis of randomized controlled trials

Meenakshi Dauwan*, Marieke JH Begemann, Margot IE Slot, Edwin HM Lee, Philip Scheltens, Iris EC Sommer

*** Corresponding author:**
Meenakshi Dauwan, M.D.
Neuroimaging Center, University Medical Center Groningen
Department of Clinical Neurophysiology and MEG Center, Amsterdam UMC, Vrije Universiteit Amsterdam
Department of Psychiatry, University Medical Center Utrecht
Neuroimaging Center 3111
Antonius Deusinglaan 2
9713 AW Groningen, The Netherlands
Tel: +31 88 75 57468
E-mail: m.dauwan@umcg.nl; m.dauwan-3@umcutrecht.nl

Table S5: Results for all outcome measures based on studies that performed intention-to-treat (ITT) analysis

Outcome measure	Studies (N)	Patients (IG/CG)	Hedges' g	95% CI	p-value	Q-statistic (df)	I^2 (%)	Egger's test	N_R
**QoL**	38	1277/1228	**0.56**	**0.37 to 0.74**	**<.0001**	**Q(37)=182.89, p<.0001**	**80**	**t(36)=4.29, p<.0001**	**1132**
Without outlier	33	1222/1179	**0.44**	**0.27 to 0.61**	**<.0001**	**Q(32)=127.91, p<.0001**	**75**	**t(31)=3.18, p=.003**	**636**
**Depressive symptoms**	37	1027/808	**0.90**	**0.62 to 1.18**	**<.0001**	**Q(36)=268.64, p<.0001**	**87**	**t(35)=4.76, p<.0001**	**1808**
Without outlier	26	873/682	**0.62**	**0.38 to 0.86**	**<.0001**	**Q(25)=119.83, p<.0001**	**79**	**t(24)=3.66, p=.001**	**617**
**Cognition**									
**Attention & Working memory**	14	586/372	0.21	-0.03 to 0.46	.089	Q(13)=32.54, p=.002	60	t(12)=1.94, p=.076	
Without outlier	8	533/321	0.12	-0.20 to 0.43	.462	Q(7)=26.78, p=<.0001	74	t(6)=0.98, p=.366	
**Executive functioning**	9	441/289	0.12	-0.04 to 0.28	.152	Q(8)=9.54, p=.299	16	t(7)=0.24, p=.814	
Without outlier	5	410/259	0.13	-0.02 to 0.27	.086	Q(4)=2.06, p=.724	0	t(3)=1.78, p=.173	
**Memory**	8	477/310	0.09	-0.04 to 0.21	.191	Q(7)=4.21, p=.755	0	t(6)=0.48, p=.649	
Without outlier	6	461/292	0.08	-0.06 to 0.21	.265	Q(5)=3.73, p=.589	0	t(4)=1.56, p=.194	
**Psychomotor speed**	12	369/303	**0.24**	**0.05 to 0.43**	**.015**	Q(11)=15.60, p=.157	29	**t(10)=2.28, p=.046**	**22**
Without outlier	6	314/248	0.10	-0.05 to 0.25	.196	Q(5)=4.46, p=.485	0	t(4)=0.62, p=.570	
**Verbal fluency**	5	292/227	0.20	-0.13 to 0.53	.229	Q(4)=12.89, p=.012	69	t(3)=2.67, p=.076	
Without outliers	4	277/212	0.02	-0.17 to 0.21	.825	Q(3)=3.77, p=.287	20	t(2)=1.47, p=.280	
**Global cognition**	12	335/306	0.33	-0.07 to 0.73	.108	Q(11)=60.23, p<.0001	82	t(10)=0.19, p=.855	
Without outliers	8	290/266	**0.60**	**0.19 to 1.00**	**.004**	**Q(7)=34.55, p<.0001**	**80**	t(6)=2.25, p=.065	

CG=Control group; df=degrees of freedom; IG=Intervention group; N_R =Rosenthal's fail-safe number
