# Supplementary material for: Physical exercise improves quality of life, depressive symptoms, and cognition across chronic brain disorders: a transdiagnostic systematic review and meta-analysis of randomized controlled trials
Source: J Neurol. 2019 Aug 14;268(4):1222–46. doi: 10.1007/s00415-019-09493-9 (PMC7990819; doi:10.1007/s00415-019-09493-9)
Supplement: Supplementary file 7 — Supplementary file7 (PDF 98 kb) [file 415_2019_9493_MOESM7_ESM.pdf]

Physical exercise improves quality of life, depressive symptoms, and cognition across chronic brain disorders: a transdiagnostic systematic review and meta-analysis of randomized controlled trials

Meenakshi Dauwan*, Marieke JH Begemann, Margot IE Slot, Edwin HM Lee, Philip Scheltens, Iris EC Sommer

*** Corresponding author:**
Meenakshi Dauwan, M.D.
Neuroimaging Center, University Medical Center Groningen
Department of Clinical Neurophysiology and MEG Center, Amsterdam UMC, Vrije Universiteit Amsterdam
Department of Psychiatry, University Medical Center Utrecht
Neuroimaging Center 3111
Antonius Deusinglaan 2
9713 AW Groningen, The Netherlands
Tel: +31 88 75 57468
E-mail: m.dauwan@umcg.nl; m.dauwan-3@umcutrecht.nl

Table S6: Results of meta-regression analysis

Outcome measure	Meta-regression variable	Beta	95% CI	p-value
**QoL**	Weekly amount of exercise (min/week)	-0.000	-0.002 to 0.002	.973
	Length of intervention (weeks)	-0.009	-0.025 to 0.008	.311
	Mean age	-0.007	-0.015 to 0.002	.144
**Depressive symptoms**	Weekly amount of exercise (min/week)	**0.007**	**0.002 to 0.013**	**.012**
	Length of intervention (weeks)	-0.016	-0.039 to 0.008	.190
	Mean age	0.001	-0.014 to 0.017	.873
**Cognition**				
**Attention & Working Memory**	Weekly amount of exercise (min/week)	0.002	-0.002 to 0.005	.331
	Length of intervention (weeks)	0.007	-0.006 to 0.020	.299
	Mean age	0.005	-0.007 to 0.016	.425
**Executive Functioning**	Weekly amount of exercise (min/week)	-0.000	-0.001 to 0.001	.532
	Length of intervention (weeks)	0.001	-0.007 to 0.009	.793
	Mean age	-0.001	-0.009 to 0.006	.726
**Memory**	Weekly amount of exercise (min/week)	-0.000	-0.001 to 0.001	.761
	Length of intervention (weeks)	-0.009	-0.025 to 0.007	.281
	Mean age	-0.002	-0.010 to 0.005	.527
**Psychomotor Speed**	Weekly amount of exercise (min/week)	-0.001	-0.005 to 0.002	.504
	Length of intervention (weeks)	-0.018	-0.039 to 0.003	.088
	Mean age	-0.007	-0.016 to 0.002	.116
**Global Cognition**	Weekly amount of exercise (min/week)	-0.001	-0.002 to 0.000	.158
	Length of intervention (weeks)	0.001	-0.014 to 0.015	.931
	Mean age	-0.001	-0.031 to 0.028	.939
