# Supplementary material for: Physical exercise improves quality of life, depressive symptoms, and cognition across chronic brain disorders: a transdiagnostic systematic review and meta-analysis of randomized controlled trials
Source: J Neurol. 2019 Aug 14;268(4):1222–46. doi: 10.1007/s00415-019-09493-9 (PMC7990819; doi:10.1007/s00415-019-09493-9)
Supplement: Supplementary file 12 — Supplementary file12 (PDF 148 kb) [file 415_2019_9493_MOESM12_ESM.pdf]

Physical exercise improves quality of life, depressive symptoms, and cognition across chronic brain disorders: a transdiagnostic systematic review and meta-analysis of randomized controlled trials

Meenakshi Dauwan*, Marieke JH Begemann, Margot IE Slot, Edwin HM Lee, Philip Scheltens, Iris EC Sommer

*** Corresponding author:**
Meenakshi Dauwan, M.D.
Neuroimaging Center, University Medical Center Groningen
Department of Clinical Neurophysiology and MEG Center, Amsterdam UMC, Vrije Universiteit Amsterdam
Department of Psychiatry, University Medical Center Utrecht
Neuroimaging Center 3111
Antonius Deusinglaan 2
9713 AW Groningen, The Netherlands
Tel: +31 88 75 57468
E-mail: m.dauwan@umcg.nl; m.dauwan-3@umcutrecht.nl

Supplementary figure 2: results of quality of life

Study	Hedges' g	p-value	Intervention (N)	Control (N)	95% CI per study	Std residual	p-value
Aguiar 2014	0.569	0.096	17	17	-0.101 to 1.239	0.32	0.75
Ahmadi 2010a	0.696	0.075	10	10	-0.071 to 1.463	0.53	0.59
Ahmadi 2010b	0.714	0.057	11	10	-0.022 to 1.450	0.58	0.56
Allen 2010	0.195	0.507	21	24	-0.382 to 0.772	-0.41	0.68
Ashburn 2007	-0.372	0.033	67	66	-0.712 to -0.031	-1.75	0.08
Battaglia 2013	2.319	0.000	10	8	1.310 to 3.328	2.94	0.00
Belton 2014	-0.186	0.639	12	12	-0.960 to 0.589	-1.03	0.30
Brenes 2007	-0.595	0.082	14	12	-1.265 to 0.075	-1.88	0.06
Busse 2013	0.923	0.027	9	9	0.103 to 1.743	0.91	0.36
Cakit 2010	0.190	0.575	24	9	-0.475 to 0.855	-0.39	0.69
Canning 2012	-0.088	0.844	9	9	-0.969 to 0.792	-0.81	0.42
Canning 2014	-0.111	0.311	104	115	-0.326 to 0.104	-1.22	0.22
Carroll 2017	0.604	0.192	10	8	-0.303 to 1.511	0.34	0.74
Carta 2008	0.379	0.253	10	20	-0.271 to 1.029	-0.04	0.97
Carter 2014	0.425	0.008	60	60	0.109 to 0.741	0.06	0.95
Carter 2015	0.175	0.412	44	43	-0.243 to 0.592	-0.49	0.62
Cholewa 2013	0.711	0.004	40	30	0.229 to 1.194	0.67	0.51
Clarke 2016	-0.048	0.535	348	347	-0.200 to 0.104	-1.09	0.28
Dalgas 2010b	0.603	0.052	15	16	-0.004 to 1.210	0.41	0.69
Dodd 2011	0.348	0.141	36	35	-0.116 to 0.812	-0.11	0.92
Doulatabad 2013	1.262	0.000	30	30	0.713 to 1.810	1.76	0.08
Duff 2018	0.108	0.726	15	15	-0.496 to 0.712	-0.57	0.57
Ebrahimi 2015	0.295	0.344	16	14	-0.315 to 0.905	-0.20	0.84
Garett 2012a	0.358	0.008	193	49	0.095 to 0.621	-0.09	0.93
Goodwin 2011	-0.101	0.575	61	62	-0.452 to 0.251	-1.13	0.26
Hoffmann 2015	-0.139	0.338	102	88	-0.423 to 0.145	-1.25	0.21
Hogan 2014	0.198	0.182	97	15	-0.093 to 0.489	-0.46	0.64
Huang 2015	1.026	0.000	19	20	0.458 to 1.594	1.27	0.20

Ikai 2013	-0.128	0.649	25	24	-0.680 to 0.424	-1.07	0.28
Kaltsatou 2014	0.979	0.008	16	15	0.251 to 1.707	1.06	0.29
Kargarfard 2012	3.368	0.000	10	11	2.234 to 4.502	4.22	0.00
Keus 2007	-0.449	0.235	14	13	-1.191 to 0.292	-1.53	0.13
Khalil 2013	-0.076	0.835	11	10	-0.791 to 0.639	-0.87	0.38
Khan 2008	0.031	0.862	46	46	-0.320 to 0.382	-0.83	0.41
Learmonth 2012	0.081	0.821	20	12	-0.617 to 0.779	-0.59	0.56
Learmonth 2017	0.076	0.770	29	28	-0.436 to 0.589	-0.67	0.50
Liao 2015a	1.853	0.000	12	12	0.919 to 2.787	2.34	0.02
Lin 2015	0.525	0.007	69	33	0.143 to 0.907	0.29	0.77
Maci 2012	1.862	0.002	7	7	0.663 to 3.061	2.00	0.05
Miller 2011	0.815	0.028	15	15	0.089 to 1.541	0.76	0.44
Negahban 2013	0.558	0.109	12	12	-0.124 to 1.240	0.30	0.76
Ni 2016a	1.062	0.015	13	10	0.210 to 1.914	1.12	0.26
Ni 2016b	0.777	0.061	14	10	-0.036 to 1.591	0.66	0.51
Oken 2004	-0.013	0.960	37	19	-0.517 to 0.491	-0.86	0.39
Paul 2014	0.225	0.535	15	14	-0.485 to 0.935	-0.32	0.75
Plow 2014	0.417	0.247	14	16	-0.289 to 1.123	0.04	0.97
Quinn 2014	0.177	0.631	15	13	-0.546 to 0.900	-0.40	0.69
Qutubuddin 2013	0.287	0.482	13	10	-0.512 to 1.086	-0.19	0.85
Romberg 2005	0.051	0.772	47	48	-0.294 to 0.396	-0.79	0.43
Romenets 2015	0.053	0.876	18	15	-0.615 to 0.722	-0.65	0.51
Sangelaji 2014	0.821	0.001	39	22	0.357 to 1.285	0.91	0.36
Santos 2017	1.556	0.000	13	15	0.728 to 2.384	1.99	0.05
Schuch 2015	0.872	0.001	25	25	0.376 to 1.368	1.00	0.32
Silva-Batista 2016	1.121	0.002	26	13	0.414 to 1.828	1.34	0.18
Singh 1997b	0.366	0.225	17	15	-0.225 to 0.957	-0.06	0.95
Storr 2006	-0.306	0.154	37	50	-0.732 to 0.115	-1.55	0.12
Tarakci 2013	0.238	0.234	51	48	-0.154 to 0.631	-0.35	0.72
Teri 2003	0.668	0.000	68	72	0.329 to 1.007	0.62	0.54
Tickle-Degnen 2010	0.902	0.000	39	40	0.443 to 1.361	1.08	0.28
Vermohlen 2018	0.868	0.000	30	37	0.413 to 1.323	1.01	0.31
Visceglia 2011	1.211	0.005	10	8	0.370 to 2.052	1.38	0.17
Wade 2003	-0.199	0.243	53	41	-0.533 to 0.135	-1.37	0.17
Yeung 2012	0.000	1.000	25	13	-0.656 to 0.656	-0.76	0.45
Zhang 2004	-0.119	0.592	40	40	-0.553 to 0.316	-1.12	0.26
	**0.397**	**0.000**	**2349**	**1985**	0.273 to 0.521		
