# Supplementary material for: Physical exercise improves quality of life, depressive symptoms, and cognition across chronic brain disorders: a transdiagnostic systematic review and meta-analysis of randomized controlled trials
Source: J Neurol. 2019 Aug 14;268(4):1222–46. doi: 10.1007/s00415-019-09493-9 (PMC7990819; doi:10.1007/s00415-019-09493-9)
Supplement: Supplementary file 13 — Supplementary file13 (PDF 147 kb) [file 415_2019_9493_MOESM13_ESM.pdf]

Physical exercise improves quality of life, depressive symptoms, and cognition across chronic brain disorders: a transdiagnostic systematic review and meta-analysis of randomized controlled trials

Meenakshi Dauwan*, Marieke JH Begemann, Margot IE Slot, Edwin HM Lee, Philip Scheltens, Iris EC Sommer

*** Corresponding author:**
Meenakshi Dauwan, M.D.
Neuroimaging Center, University Medical Center Groningen
Department of Clinical Neurophysiology and MEG Center, Amsterdam UMC, Vrije Universiteit Amsterdam
Department of Psychiatry, University Medical Center Utrecht
Neuroimaging Center 3111
Antonius Deusinglaan 2
9713 AW Groningen, The Netherlands
Tel: +31 88 75 57468
E-mail: m.dauwan@umcg.nl; m.dauwan-3@umcutrecht.nl

Supplementary figure 3: results of depressive symptoms

Study	Hedges' g	p-value	Intervention (N)	Control (N)	95% CI per study	Std residual	p-value
Ahmadi 2013	0.580	0.122	21	10	-0.156 to 1.316	-0.26	0.80
Blumenthal 2007	0.153	0.377	104	49	-0.186 to 0.492	-0.89	0.38
Brenes 2007	1.026	0.012	14	12	0.229 to 1.823	0.30	0.76
Briken 2014	0.768	0.078	11	10	-0.086 to 1.622	-0.02	0.98
Cakit 2010	0.024	0.949	24	9	-0.717 to 0.765	-0.96	0.33
Carneiro 2015	0.942	0.043	9	10	0.032 to 1.853	0.19	0.85
Carter 2015	0.245	0.251	44	43	-0.173 to 0.663	-0.74	0.46
Chan 2012	0.795	0.025	17	16	0.102 to 1.488	0.01	0.99
Chou 2004	2.218	0.001	7	7	0.939 to 3.497	1.51	0.13
Cugusi 2015	0.549	0.209	10	10	-0.308 to 1.405	-0.29	0.77
Dalgas 2010b	0.703	0.052	15	16	-0.005 to 1.411	-0.10	0.92
Danielsson 2014	2.043	0.000	42	20	1.361 to 2.725	1.63	0.10
Doose 2015	1.614	0.000	30	16	0.932 to 2.295	1.07	0.28
Foster 2013	-0.601	0.032	26	26	-1.148 to -0.053	-1.86	0.06
Hebert 2012	0.328	0.329	25	13	-0.331 to 0.987	-0.59	0.55
Hoffmann 2015	0.028	0.848	102	88	-0.256 to 0.312	-1.07	0.28
Huang 2015	0.925	0.005	19	20	0.277 to 1.574	0.18	0.85
Kerling 2015	0.355	0.245	22	20	-0.244 to 0.954	-0.57	0.57
Khatri 2001	0.212	0.328	42	42	-0.213 to 0.637	-0.79	0.43
Kinser 2014	0.418	0.386	12	6	-0.526 to 1.361	-0.43	0.66
Learmonth 2012	0.348	0.332	20	12	-0.355 to 1.051	-0.56	0.58
Learmonth 2017	0.158	0.546	29	28	-0.355 to 0.671	-0.85	0.40
Legrand 2015	1.619	0.000	15	12	0.766 to 2.472	1.02	0.31
Legrand 2016	1.121	0.009	14	10	0.276 to 1.966	0.41	0.68
Lin 2015	0.615	0.037	42	15	0.037 to 1.193	-0.22	0.82
Luttenberger 2015	0.752	0.012	22	25	0.169 to 1.336	-0.04	0.97
Maci 2012	2.247	0.001	7	7	0.961 to 3.533	1.54	0.12
Marzolini 2009	1.287	0.048	4	6	0.013 to 2.561	0.53	0.60

Miller 2011	0.075	0.832	15	15	-0.621 to 0.772	-0.91	0.36
Mota-Pereira 2011	5.470	0.000	19	10	3.878 to 7.062	4.39	0.00
Murri 2015	0.659	0.001	79	42	0.277 to 1.041	-0.17	0.86
Niemi 2016	2.349	0.000	28	18	1.594 to 3.103	1.98	0.05
Oertel-Knöchel 2014 (Dep)	0.496	0.302	8	8	-0.446 to 1.438	-0.34	0.73
Oken 2004	0.166	0.547	37	20	-0.374 to 0.706	-0.83	0.41
Paul 2014	0.069	0.848	15	14	-0.639 to 0.778	-0.92	0.36
Petajan 1996	0.400	0.174	21	25	-0.176 to 0.976	-0.51	0.61
Picelli 2016	0.579	0.220	9	8	-0.346 to 1.504	-0.24	0.81
Pilu 2007	1.184	0.004	10	20	0.387 to 1.981	0.50	0.62
Prakhinkit 2014	2.395	0.000	27	13	1.497 to 3.293	1.94	0.05
Prathikanti 2017	-0.931	0.025	15	10	-1.747 to -0.116	-2.13	0.03
Quinn 2014	-0.157	0.670	15	13	-0.879 to 0.565	-1.20	0.23
Razazian 2016	2.752	0.000	36	18	1.970 to 3.534	2.47	0.01
Rolland 2007	-0.131	0.447	67	67	-0.468 to 0.206	-1.28	0.20
Romberg 2005	-0.148	0.469	47	48	-0.547 to 0.252	-1.29	0.20
Romenets 2015	0.032	0.925	18	15	-0.637 to 0.701	-0.98	0.33
Schuch 2015	0.718	0.013	25	25	0.154 to 1.281	-0.09	0.93
Shahidi 2011	0.786	0.005	40	20	0.239 to 1.333	0.00	1.00
Sharma 2017	5.267	0.000	13	12	3.621 to 6.912	4.12	0.00
Singh 1997b	3.374	0.000	17	15	2.306 to 4.443	2.94	0.00
Singh 2001	1.691	0.000	15	14	0.860 to 2.522	1.12	0.26
Singh 2005	0.500	0.081	35	19	-0.061 to 1.061	-0.38	0.70
Siqueira 2016	0.008	0.979	20	28	-0.557 to 0.572	-1.04	0.30
Sutherland 2001	0.588	0.161	11	11	-0.235 to 1.410	-0.24	0.81
Teri 2003	0.239	0.157	68	72	-0.092 to 0.570	-0.77	0.44
Thompson 2013	1.026	0.026	9	11	0.124 to 1.928	0.29	0.77
Tsang 2006	0.861	0.000	48	34	0.406 to 1.315	0.11	0.92
Tsang 2012	0.497	0.125	21	17	-0.139 to 1.133	-0.38	0.71
Vreugdenhil 2012	2.691	0.000	20	20	1.844 to 3.538	2.34	0.02
Wade 2003	0.091	0.658	53	41	-0.313 to 0.496	-0.96	0.34
Yeung 2012	-0.156	0.641	25	13	-0.813 to 0.501	-1.22	0.22
	**0.784**	**0.000**	**1635**	**1274**	0.592 to 1.011		
