# Supplementary material for: Physical exercise improves quality of life, depressive symptoms, and cognition across chronic brain disorders: a transdiagnostic systematic review and meta-analysis of randomized controlled trials
Source: J Neurol. 2019 Aug 14;268(4):1222–46. doi: 10.1007/s00415-019-09493-9 (PMC7990819; doi:10.1007/s00415-019-09493-9)
Supplement: Supplementary file 14 — Supplementary file14 (PDF 182 kb) [file 415_2019_9493_MOESM14_ESM.pdf]

Physical exercise improves quality of life, depressive symptoms, and cognition across chronic brain disorders: a transdiagnostic systematic review and meta-analysis of randomized controlled trials

Meenakshi Dauwan*, Marieke JH Begemann, Margot IE Slot, Edwin HM Lee, Philip Scheltens, Iris EC Sommer

*** Corresponding author:**
Meenakshi Dauwan, M.D.
Neuroimaging Center, University Medical Center Groningen
Department of Clinical Neurophysiology and MEG Center, Amsterdam UMC, Vrije Universiteit Amsterdam
Department of Psychiatry, University Medical Center Utrecht
Neuroimaging Center 3111
Antonius Deusinglaan 2
9713 AW Groningen, The Netherlands
Tel: +31 88 75 57468
E-mail: m.dauwan@umcg.nl; m.dauwan-3@umcutrecht.nl

Supplementary figure 4: results of cognition

Attention & Working Memory

**Study**	**Hedges' g**	**p-value**	**Intervention (N)**	**Control (N)**	**95% CI per study**	**Std residual**	**p-value**
Arcoverde 2014	-0.294	0.495	10	10	-1.138 to 0.551	-1.05	0.29
Belton 2014	0.029	0.942	12	12	-0.744 to 0.801	-0.44	0.66
Bhatia 2017	-0.478	0.004	194	92	-0.806 to -0.150	-2.29	0.02
Briken 2014	0.838	0.028	11	10	0.092 to 1.584	1.30	0.19
Chan 2012	0.866	0.015	17	16	0.168 to 1.564	1.43	0.15
Conradsson 2015	0.599	0.006	45	42	0.173 to 1.026	1.07	0.28
Duncan 2014	1.099	0.077	5	5	-0.120 to 2.318	1.28	0.20
Ho 2016	0.141	0.416	102	49	-0.198 to 0.480	-0.30	0.77
Hoang 2015	0.115	0.699	23	21	-0.467 to 0.696	-0.30	0.76
Hoffman 2008	0.119	0.491	104	49	-0.219 to 0.457	-0.37	0.71
Khatri 2001	-0.288	0.186	42	42	-0.714 to 0.138	-1.53	0.13
Lin 2015	0.498	0.019	69	33	0.082 to 0.914	0.78	0.44
Nilsagard 2013	0.316	0.156	41	39	-0.120 to 0.753	0.24	0.81
Oertel Knöchel 2014 (Dep)	0.559	0.247	8	8	-0.388 to 1.505	0.59	0.56
Oertel Knöchel 2014 (Sz)	0.220	0.627	8	10	-0.668 to 1.108	-0.03	0.98
Oken 2004	-0.041	0.862	37	20	-0.505 to 0.423	-0.78	0.44
Picelli 2016	0.541	0.250	9	8	-0.381 to 1.463	0.57	0.57
Romenets 2015	0.418	0.225	18	15	-0.258 to 1.095	0.42	0.67
Sandroff 2016	0.202	0.724	5	5	-0.921 to 1.325	-0.05	0.96
Suttanon 2012	0.461	0.143	19	21	-0.156 to 1.077	0.55	0.58
Yaguez 2011	0.414	0.170	15	12	-0.177 to 1.005	0.45	0.65
	**0.234**	**0.000**	**794**	**519**	**0.058 to 0.411**		

Executive functioning

Study	Hedges' g	p-value	Intervention (N)	Control (N)	95% CI per study	Std residual	p-value
Arcoverde 2014	-0.596	0.098	10	10	-1.303 to 0.111	-2.10	0.04
Bhatia 2017	0.000	1.000	194	92	-0.248 to 0.248	-1.36	0.17
Briken 2014	0.496	0.245	11	10	-0.340 to 1.332	0.82	0.41
De Oliveira 2016	0.078	0.827	15	8	-0.620 to 0.776	-0.21	0.83
Hoang 2015	0.053	0.858	23	21	-0.528 to 0.634	-0.34	0.73
Hoffman 2008	0.185	0.191	104	49	-0.092 to 0.462	-0.26	0.79
Khatri 2001	0.232	0.188	42	42	-0.113 to 0.577	0.49	0.63
Lin 2015	0.394	0.043	69	33	0.012 to 0.776	1.31	0.19
Maci 2012	0.759	0.145	7	7	-0.261 to 1.779	1.18	0.24
Ohman 2016a	0.118	0.505	61	65	-0.229 to 0.466	-0.20	0.84
Oken 2004	0.231	0.361	37	20	-0.265 to 0.727	0.32	0.75
Picelli 2016	0.532	0.192	9	8	-0.267 to 1.331	0.94	0.34
Sandroff 2016	-0.382	0.445	5	5	-1.362 to 0.598	-1.07	0.28
Thompson 2013	0.405	0.289	9	11	-0.344 to 1.154	0.67	0.50
	**0.151**	**0.013**	**596**	**381**	**0.032 to 0.270**		

Memory

Study	Hedges' g	p-value	Intervention (N)	Control (N)	95% CI per study	Std residual	p-value
Arcoverde 2014	-0.273	0.527	10	10	-1.116 to 0.571	-0.93	0.35
Bhatia 2017	0.060	0.635	194	92	-0.188 to 0.308	-0.56	0.58
Briken 2014	1.029	0.008	11	10	0.265 to 1.793	2.35	0.02
Hoffman 2008	0.090	0.525	104	49	-0.187 to 0.367	-0.25	0.80
Hoffmann 2015	0.063	0.618	102	88	-0.185 to 0.311	-0.53	0.59
Khatri 2001	0.273	0.115	42	42	-0.066 to 0.612	0.92	0.36
Lin 2015	0.264	0.170	69	33	-0.113 to 0.641	0.77	0.44
Oertel Knöchel 2014 (Dep)	0.300	0.467	8	8	-0.508 to 1.108	0.43	0.66
Oertel Knöchel 2014 (Sz)	0.254	0.519	8	10	-0.518 to 1.026	0.34	0.74
Oken 2004	0.000	1.000	37	20	-0.540 to 0.540	-0.46	0.65
Thompson 2013	-0.378	0.271	9	11	-1.051 to 0.295	-1.48	0.14
Yaguez 2011	0.232	0.594	15	12	-0.620 to 1.084	0.25	0.80
	**0.123**	**0.038**	**609**	**385**	**0.007 to 0.238**		

Psychomotor speed

Study	Hedges' g	p-value	Intervention (N)	Control (N)	95% CI per study	Std residual	p-value
Arcoverde 2014	0.402	0.353	10	10	-0.446 to 1.251	0.39	0.69
Briken 2014	0.464	0.275	11	10	-0.370 to 1.298	0.54	0.59
Hoang 2015	0.288	0.335	23	21	-0.297 to 0.872	0.19	0.84
Hoffman 2008	0.229	0.186	104	49	-0.110 to 0.568	0.01	0.99
Hoffmann 2015	-0.034	0.788	102	88	-0.282 to 0.214	-1.55	0.12
Holthoff 2015	1.257	0.001	15	15	0.492 to 2.023	2.54	0.01
Khatri 2001	0.060	0.725	42	42	-0.274 to 0.394	-0.82	0.41
Lin 2015	0.436	0.040	69	33	0.020 to 0.852	0.87	0.38
Oertel Knöchel 2014 (Dep)	0.732	0.135	8	8	-0.229 to 1.692	1.00	0.31
Oertel Knöchel 2014 (Sz)	0.508	0.269	8	10	-0.393 to 1.409	0.59	0.55
Oken 2004	-0.121	0.632	37	20	-0.617 to 0.375	-1.26	0.20

Picelli 2016	0.192	0.677	9	8	-0.714 to 1.099	-0.07	0.94
Romberg 2005	0.355	0.083	47	48	-0.047 to 0.758	0.55	0.58
Salhofer-Polanyi 2013	0.178	0.687	10	9	-0.685 to 1.040	-0.11	0.91
Sandroff 2016	0.674	0.254	5	5	-0.484 to 1.832	0.74	0.45
Thompson 2013	-0.211	0.573	9	11	-0.944 to 0.522	-1.12	0.26
	**0.227**	**0.003**	**509**	**387**	**0.075 to 0.379**		

Verbal fluency

Study	Hedges' g	p-value	Intervention (N)	Control (N)	95% CI per study	Std residual	p-value
Arcoverde 2014	0.570	0.193	10	10	-0.288 to 1.428	0.66	0.51
Briken 2014	0.594	0.166	11	10	-0.247 to 1.436	0.72	0.47
Hoffman 2008	0.168	0.268	104	49	-0.129 to 0.465	-0.25	0.81
Hoffmann 2015	-0.111	0.380	102	88	-0.359 to 0.137	-1.27	0.20
Holthoff 2015	1.210	0.002	15	15	0.449 to 1.971	2.12	0.03
Ohman 2016a	-0.055	0.757	61	65	-0.402 to 0.292	-0.97	0.33
	**0.238**	**0.134**	**303**	**237**	**-0.074 to 0.551**		

Global cognition

Study	Hedges' g	p-value	Intervention (N)	Control (N)	95% CI per study	Std residual	p-value
Aguiar 2014	0.184	0.584	17	17	-0.474 to 0.842	-0.19	0.85
Arcoverde 2014	1.681	0.000	10	10	0.820 to 2.542	2.03	0.04
Busse 2013	0.260	0.534	9	13	-0.561 to 1.082	-0.06	0.95
Hoffmann 2015	0.166	0.253	102	88	-0.118 to 0.451	-0.25	0.81
Holthoff 2015	-0.284	0.427	15	15	-0.984 to 0.416	-0.92	0.36
Kemoun 2010	1.054	0.005	16	15	0.320 to 1.789	1.18	0.24
Maci 2012	-0.311	0.538	7	7	-1.298 to 0.677	-0.84	0.40
Ohman 2016a	-0.034	0.847	61	65	-0.382 to 0.313	-0.61	0.54
Picelli 2016	0.285	0.539	9	8	-0.624 to 1.194	-0.02	0.98
Quinn 2014	0.047	0.899	15	13	-0.674 to 0.768	-0.40	0.69
Romenets 2015	0.368	0.285	18	15	-0.307 to 1.042	0.11	0.91
Silva-Batista 2016	1.450	0.000	26	13	0.693 to 2.207	1.78	0.08
Venturelli 2011	-2.400	0.000	11	10	-3.497 to -1.303	-3.53	0.00
Vreugdenhil 2012	0.505	0.065	20	20	-0.032 to 1.042	0.35	0.73
Zhang 2004	0.815	0.000	40	40	0.363 to 1.267	0.91	0.36
	**0.299**	**0.076**	**376**	**349**	**-0.031 to 0.629**		
